# Genome-wide analysis of glyoxalase-like gene families in grape (*Vitis vinifera* L.) and their expression profiling in response to downy mildew infection

**DOI:** 10.1186/s12864-019-5733-y

**Published:** 2019-05-09

**Authors:** Tiemei Li, Xin Cheng, Yuting Wang, Xiao Yin, Zhiqian Li, Ruiqi Liu, Guotian Liu, Yuejin Wang, Yan Xu

**Affiliations:** 1 College of Horticulture, Northwest A&F University, Yangling, Shaanxi People’s Republic of China; 2 State Key Laboratory of Crop Stress Biology for Arid Areas, Northwest A&F University, Yangling, Shaanxi People’s Republic of China; 3 Key Laboratory of Horticultural Plant Biology and Germplasm Innovation in Northwest China, Ministry of Agriculture, Yangling, Shaanxi People’s Republic of China

**Keywords:** Glyoxalase, Methylglyoxal, Grapevine, Downy mildew, qRT-PCR

## Abstract

**Background:**

The glyoxalase system usually comprises two enzymes, glyoxalase I (GLYI) and glyoxalase II (GLYII). This system converts cytotoxic methylglyoxal (MG) into non-toxic D-lactate in the presence of reduced glutathione (GSH) in two enzymatic steps. Recently, a novel type of glyoxalase III (GLYIII) activity has observed in *Escherichia coli* that can detoxify MG into D-lactate directly, in one step, without a cofactor. Investigation of the glyoxalase enzymes of a number of plant species shows the importance of their roles in response both to abiotic and to biotic stresses. Until now, glyoxalase gene families have been identified in the genomes of four plants, *Arabidopsis*, *Oryza sativa*, *Glycine max* and *Medicago truncatula* but no similar study has been done with the grapevine *Vitis vinifera* L.

**Results:**

In this study, four *GLYI-like*, two *GLYII-like* and three *GLYIII-like* genes are identified from the genome database of grape. All these genes were analysed in detail, including their chromosomal locations, phylogenetic relationships, exon-intron distributions, protein domain organisations and the presence of conserved binding sites. Using quantitative real-time PCR analysis (qRT-PCR), the expression profiles of these genes were analysed in different tissues of grape*,* and also when under infection stress from downy mildew (*Plasmopara viticola*). The study reveals that most *VvGLY-like* genes had higher expressions in stem, leaf, tendril and ovule but lower expressions in the flower. In addition, most of the *VvGLY-like* gene members were *P. viticola* responsive with high expressions 6-12 h and 96-120 h after inoculation. However, *VvGLYI-like1* was highly expressed 48 h after inoculation, similar to *VvPR1* and *VvNPR1* which are involved in the defence response.

**Conclusions:**

This study identified the *GLYI-like*, *GLYII-like* and *GLYIII-like* full gene families of the grapevine*.* Based on a phylogenetic analysis and the presence of conserved binding sites, we speculate that these *glyoxalase-like* genes in grape encode active glyoxalases. Moreover, our study provides a basis for discussing the roles of *VvGLYI-like*, *VvGLYII-like* and *VvGLYIII-like* genes in grape’s response to downy mildew infection. Our results shed light on the selection of candidate genes for downy mildew tolerance in grape and lay the foundation for further functional investigations of these glyoxalase genes.

**Electronic supplementary material:**

The online version of this article (10.1186/s12864-019-5733-y) contains supplementary material, which is available to authorized users.

## Background

The glyoxalase system is an almost ubiquitous pathway for the detoxification of methylglyoxal (MG) in living systems. The typical glyoxalase system can detoxify the highly cytotoxic compound, MG to the nontoxic D-lactate through the sequential activity of two enzymes - glyoxalase І (GLYI; lactoylglutathione lyase) and glyoxalase II (GLYII; hydroxyacylglutathione hydrolase). First, MG is converted into hemithioacetal (HTA) in the presence of reduced glutathione (GSH), then GLYI catalyses the HTA isomerisation into S-D-lactoyl-glutathione (SLG). Subsequently, SLG is hydrolysed to D-lactate by GLYII, recycling GSH to the system [1, 2]. Recently, a one-step detoxification of MG has been proposed for a few organisms. This is mediated by glyoxalase III (GLYIII) enzyme without any cofactor [3–7].

Glyoxalase family identification has so far been carried out in four plant genomes *Arabidopsis*, *Oryza sativa*, *Glycine max* and *Medicago truncatula* [8–10]. These glyoxalase gene identifications have been based on sequence analysis. In the genome wide analysis, eleven *GLYI* genes have been described in *Arabidopsis thaliana* and in rice [8]. However, further study revealed that only three *Arabidopsis thaliana* genes (*AtGLYI2*, *AtGLYI3* and *AtGLYI6*) and two rice genes (*OsGLYI8* and *OsGLYI11.2*) were functional and contained all the binding sites required for glyoxalase activity. In addition, the activity of AtGLYI2 was dependent on Zn^2+^, while the activities of AtGLYI3, AtGLYI6 and OsGLYI11.2 were dependent on Ni^2+^ [11–13]. Specifically, OsGLYI-8 did not show any strict requirement for a metal ion for its activity [14]. Similarly, two out of five GLYII proteins from *Arabidopsis thaliana* (AtGLYII2 and AtGLYII5) and two out of three GLYII proteins from rice (OsGLYII2 and OsGLYII3) showed GLYII enzyme activity [15–20]. Glyoxalase II of all species, including human, yeast and *Arabidopsis*, contains a highly conserved metal binding motif (TH*X*H*X*DH) that is also present in the family of metallo-β-lactamases, which are known to require Zn(II) [21–23]. However, the glyoxalase III enzyme does not require any cofactor and is reported to be a member of the DJ-1/Pfp1 (PARK7/*Pyrococcus furiosus* protease I) superfamily with a conserved catalytic triad Glu-Cys-His in its active site. Mutagenesis studies of Hsp31 revealed that the Cys-185 and Glu-77 were essential for catalysis, whereas His-186 was less crucial for enzymatic function, although it participates in the catalytic process [4, 24]. Site-directed mutagenesis of the conserved cysteine in the N-terminal domain of OsDJ-1C, which can utilise MG as substrate to produce D-lactate in a glutathione-independent manner in rice, resulted in the loss of GLYIII activity. This confirms it is a functional enzyme and that cysteine is necessary for its activity [7].

The glyoxalase system has been well studied in the animal kingdom and shown to play numerous roles in cell division and proliferation, microtubule assembly, embryogenesis, maturation and cell death [2]. An active plant glyoxalase enzyme was first reported in Douglas fir needles [25]. Thereafter, several studies have reported the presence of glyoxalase activity in both monocotyledons and dicotyledons [26–31]. It was found that over-expressed GLYI and/or GLYII in plants significantly increased their tolerance to various abiotic stresses such as salinity, drought, extreme temperature and heavy-metal toxicity [32–36]. Thus, glyoxalases have been suggested as biomarkers for plant stress resistance [31, 37]. In addition to abiotic stress responses, glyoxalase genes have also been reported to be regulated by biotic stresses. A rice *GLYI* gene was found to be down-regulated after infection by *Xanthomonas oryzae* pv. *oryzae* or by *Pyricularia grisea* [38]. Proteomic comparison analysis of maize kernel embryos showed significant up-regulation of GLYI protein in the resistant embryos in response to *Aspergillus flavus* infection [39]. Similarly, induction of GLYI activity was observed in rice in response to attack by brown planthopper (*Nilaparvata lugens*) and in *Brassica* after infection by *Sclerotinia sclerotiorum*. After fungal infection, intercellular MG levels were significantly increased in susceptible genotypes, so high GLYI activity was crucial for the detoxification of MG [39, 40].

The grapevine (*Vitis vinifera* L.) is a worldwide cultivated crop of high economic value. Sequencing of the highly homozygous grapevine PN40024 genome provided the opportunity to analyse the grapevine genome and to identify gene families [41]. Grape downy mildew, caused by the oomycete *Plasmopara viticola*, occurs in most parts of the world, especially in those where it is wet during the vegetative growth period. A major outbreak of the disease can cause severe losses in both yield and berry quality [42]. Genetic and gene expression profiling analyses show that *Rpv1*, NPR1 homologs, and PR protein encoding genes contribute to the function of downy mildew resistance in grapevine [43–45]. Moreover, overexpression of VpPR10.1 can enhance the transgenic grape plants resistance to downy mildew [46, 47]. It is important to find more crucial genes for grape downy mildew tolerance. In this study, we identify the gene families of the glyoxalase system in *V. vinifera* and discuss their roles in response to downy mildew. The existence of glyoxalases as multigene families in grape suggests there could be a number of undiscovered roles for these genes.

## Results

### Identification and detailed analysis of glyoxalase families in grape

To identify all the glyoxalase proteins in grape, lactoylglutathione lyase domain (PF00903), metallo-beta-lactamase domain (PF00753) and DJ-1/PfpI (PF01965) were downloaded from Pfam and used as queries to search the Grape Genome Database using HMMER with default E-values (< 1.0) [48]. The proteins were then manually confirmed using Pfam and BLASTP along with the previously reported GLYI proteins of *Arabidopsis*, rice, soybean and *M. truncatula* [8–10]. Proteins were identified that contained the glyoxalase domain (PF00903) and that contained all four conserved binding sites required for glyoxalase activity- i.e. (a) the active site, (b) the metal binding site, (c) the GSH binding site and (d) the dimer interface. These proteins were classified as VvGLYI-like (Table 1 and Additional file 1: Table S1) and those without all the four conserved binding sites were identified as non-glyoxalases (Additional file 2: Table S2) [8, 11]. Proteins that contained the metallo-beta-lactamase domain (PF00753) along with conserved metal binding sites (THXHXDH/H/D/H), an active site (C/GHT) and seven conserved GSH binding sites (C/K(R)/F(Y)/Y/N/R/K) and that had a putative hydroxyacyl glutathione hydrolase function were classified as VvGLYII-like (Table 1, Additional file 3: Table S3 and Additional file 4: Table S4) [8–10, 49–51]. Likewise, proteins that contained the DJ-1/PfpI (PARK7/*Pyrococcus furiosus* protease I) domain (PF01965) and Glu-Cys-His/Tyr catalytic triad were classified as VvGLYIII-like [6, 10]. A total of four GLYI-like, two GLYII-like and three GLYIII-like proteins were identified in the grape genome. These were named VvGLYI-like1 to VvGLYI-like4, VvGLYII-like1 to VvGLYII-like2 and VvGLYIII-like1 to VvGLYIII-like3 according to their location order on the grape chromosomes [8]. Meanwhile, all the grape *glyoxalase-like* genes identified were analysed in detail (Table 1). They were found to be located on eight different chromosomes. The CDS length of the *VvGLY-like* members varied from 708 bp to 1344 bp. The largest protein was VvGLYIII-like1 with a length of 447 aa, while the smallest was VvGLYI-like2 with a length of 235 aa (Table 1). Detailed information on the glyoxalase genes in grape are listed in Table 1, including their accession numbers, protein lengths, locations and similarities to the *Arabidopsis*’s orthologues.

**Table 1:** Detailed information for the GLYI, GLYII and GLYIII genes identified in *Vitis vinifera* L

Name	Accession No.	CRIBI ID	Locus ID	Chromosomal localization	Gene length (bp)	Aminoacid length (aa)	At ortholog locus	At locus description	E-value
VvGLYI-1	XM_002283932	VIT_04s0008g03560	GSVIVT01035641001	chr4:2911585 − 2,917,396	1095	364	AT1G67280	AtGLYI6	0
VvGLYI-2	XM_002276240	VIT_06s0061g00460	GSVIVT01031499001	chr6:17922434 − 17,932,956	708	235	AT1G08110	AtGLYI2	2.00E-113
VvGLYI-3	XM_002273310	VIT_10s0116g01660	GSVIVT01012714001	chr10:899745 − 919,915	879	292	AT1G11840	AtGLYI3	2.00E-117
VvGLYI-4	XM_003633073	VIT_11s0016g03440	GSVIVT01015339001	chr11:2805701 −2,814,139	1089	362	AT1G67280	AtGLYI6	0
VvGLYII-1	XM_002271759	VIT_05s0102g01180	GSVIVT01010845001	chr5:23317628 −23,328,496	990	329	AT2G43430	AtGLYII–4	6.00E-180
VvGLYII-2	XM_002267435	VIT_13s0067g00180	GSVIVT01032864001	chr13:122713 −126,313	777	258	AT3G10850	AtGLYII–5	3.00E-152
VvGLYIII-1	XM_002282219	VIT_03s0063g00300	GSVIVT01031729001	chr3:3882126 −3,887,012	1344	447	AT4G34020	DJ1C	0
VvGLYIII-2	XM_010650611	VIT_04s0079g00800	GSVIVT01035277001	chr4:11866126 −11,879,208	1161	386	AT3G02720	DJ1D	0
VvGLYIII-3	XM_010645827	VIT_19s0014g01520	GSVIVT01014220001	chr19:1617113 −1,624,250	1290	429	AT1G53280	DJ1B	0

bp, base pair; aa, amino acid; Accession No is from the NCBI database; CRIBI ID is from the UniProt database; Locus ID is from grape genoscope (http://www.genoscope.cns.fr/externe/GenomeBrowser/Vitis/); The genes from *Arabidopsis thaliana* was previously reported in reference [6, 8]

### Protein motif identification and gene structure analysis of glyoxalase-like families of grape

**Fig. 1:**
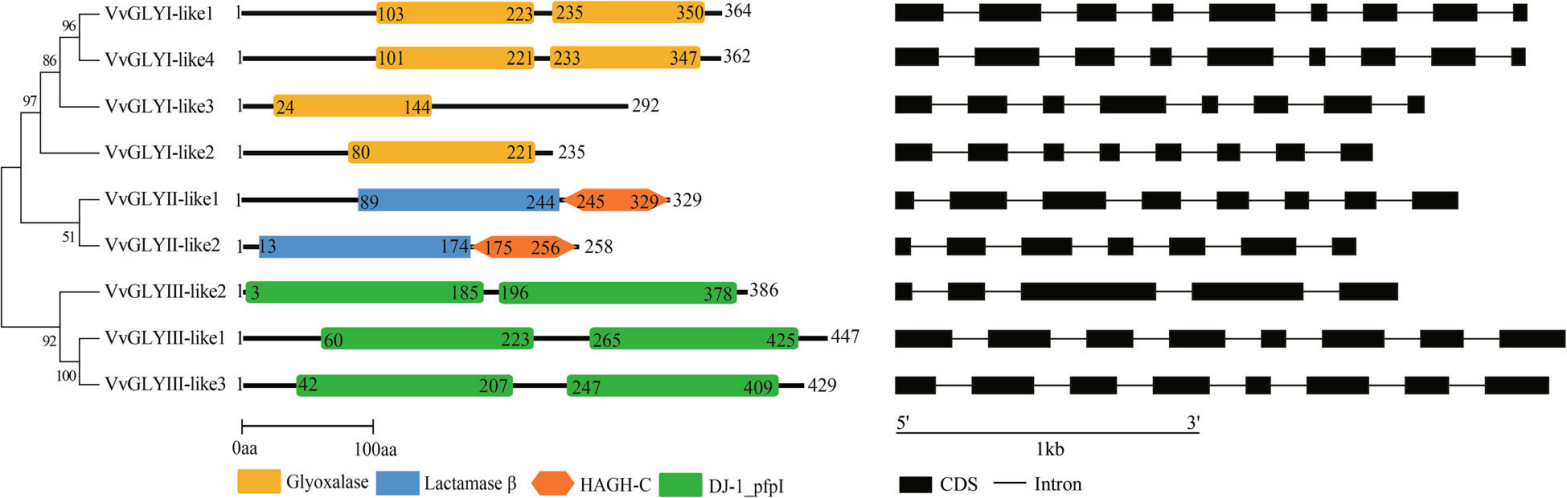
Gene structures of grape glyoxalase genes and domain architectures of grape glyoxalase proteins. The exons are represented by black boxes while the introns are represented by black lines. Only the exons are drawn to scale because some introns are too long. The glyoxalase domain (PF00903) of VvGLYI-like is represented by an orange box. For VvGLYII-like proteins, β-lactamase domain is represented by a blue box and the hydroxyacylglutathione hydrolase C-terminus (HAGH-C) domain is represented by an orange hexagon. The DJ/Pfpl domain (PF01965) of VvGLYIII-like protein is represented by a dark green box. The domain position is indicated by the number inside the box or hexagon. The full length of protein is indicated by the numbers at both ends

The exon-intron structure of the *VvGLYI-like*, *VvGLYII-like* and *VvGLYIII-like* genes was varied (Fig. 1). There were eight exons in *VvGLYI-like3* and *VvGLYI-like2* genes (Fig. 1), while *VvGLYI-like1* and *VvGLYI-like4* both had nine exons. The numbers of exons in *VvGLYII-like1* and *VvGLYII-like2* were eight and seven respectively (Fig. 1). *VvGLYIII-like1* and *VvGLYIII-like3* each had eight exons, while *VvGLYIII-like2* had five exons (Fig. 1). Genes with longer coding sequences are significantly more important and evolve more slowly than genes with shorter CDSs and they contain more functional domains within the gene [52].

All predicted VvGLYI-like proteins were analysed using Pfam to identify the glyoxalase domain (PF00903). Results indicate that VvGLYI-like1 and VvGLYI-like4 contain two glyoxalase domains while the other proteins had only a single PF00903 domain (Fig. 1). Analysis of the VvGLYII-like proteins showed that they both had a metallo-beta-lactamase domain along with a hydroxyacylglutathione hydrolase C-terminus domain (HAGH-C) (PF16123) (Fig. 1). The HAGH-C domain was a substrate binding site usually found in the C-terminus of GLYII proteins along with the catalytic domain (PF00753) [53]. The previously reported DJ-1 proteins of rice and *Arabidopsis* all contain two DJ-1/PfpI domains, while the DJ-1 proteins of *E. coli*, *Drosophila, Caenorhabditis elegans* and human contain only one [5, 7]. Structural analysis of the three grape GLYIII-like proteins show they all have two DJ-1/PfpI domains (Fig. 1). Unlike human and other homologs, the plant glyoxalases seem to have higher efficiency or better regulatory properties. Thus, plants seem to have greater adaptability to their ever-changing environments from which they are unable to escape.

### Conserved binding sites and phylogenetic analysis of operative GLYI proteins

GLYI is a metalloenzyme with conserved metal binding sites of H/QEH/QE, requiring divalent metal ions (Ni^2+^/Co^2+^ or Zn^2+^) for activation. It has been reported that GLYIs dependent on Zn^2+^ have longer domains (more than 140 aa) than Ni^2+^/Co^2+^ dependent ones (around 120 aa) and have unique regions in their sequences [11, 12, 54]. To determine metal ion dependency, the N-terminal lactoylglutathione lyasedomains of the operative GLYI proteins were aligned using ClustalW along with the human GlyI domain [55] (Additional file 5: Figure S1). Among the four VvGLYI proteins, VvGLYI-like1, VvGLYI-like3 and VvGLYI-like4 were predicted to be Ni^2+^-dependent because their domains were around 120 aa and specific Zn^2+^-dependent regions were absent. Meanwhile, VvGLYI-like2 was predicted to be Zn^2+^-dependent because of its domain length of 151 aa and because it had conserved regions for Zn^2+^-dependence (Additional file 1: Table S1 and Additional file 5: Figure S1).

**Fig. 2:**
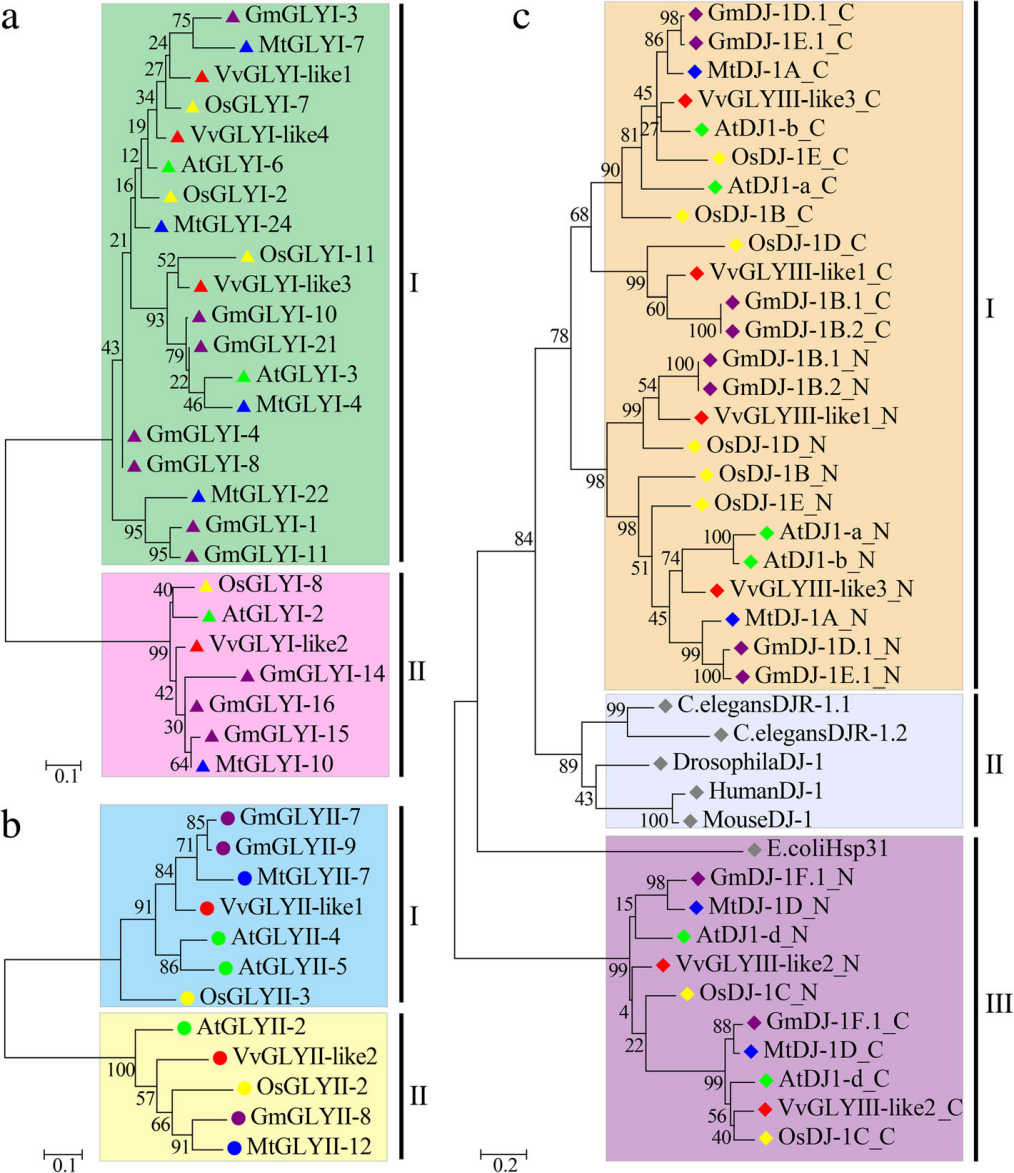
Phylogenetic relationships of GLY-like proteins from various plant species. Unrooted trees were produced using MEGA5.1 software with the Maximum Likelihood method and 1000 bootstrap replicates using the N-term domain of GLYIs (**a**), full length amino acid sequences of GLYIIs (**b**) and the two domains of GLYIIIs respectively. Glyoxalase protein sequences are provided as Additional files 13, 14 and 15. Members of the glyoxalase gene families from grape, *Arabidopsis*, rice, soybean and *Medicago truncatula* are marked by red, green, yellow, purple and blue respectively. The domains of human, mouse, *Drosophila*, *Caenorhabditis elegans* and *Escherichia coli* are marked with a white rhombus. The bootstrap values are shown near the nodes. Scale bar represents 0.1 or 0.2 amino acid substitution per site

To gain a broader understanding of the phylogeny of GLYIs, an unrooted phylogenetic tree was constructed using the Maximum Likelihood (ML) method, and based on the multiple sequence alignment of the lactoylglutathione lyase domains of the proteins listed in Additional file 1: Table S1 (Fig. 2a). According to the phylogenetic tree, the putative GLYI proteins can be divided into two subfamilies (I and II) (Fig. 2a). Subfamily I contains all the putative Ni^2+^-dependent GLYI proteins while the putative Zn^2+^-dependent GLYI proteins were in subfamily II. This provides a basis for our future research on gene functions.

### Conserved sites and phylogenetic analysis of putative GLYII proteins

The full length of the VvGLYII-like proteins and of the previously reported GLYII proteins of *Arabidopsis*, rice, soybean and *M. truncatula* [8–10] were manually analyzed using BLASTP and multiple sequence alignment to analyse for the presence of conserved binding sites required for GLYII activity (Additional file 3: Table S3, Additional file 4: Table S4 and Additional file 6: Figure S2). The putative GLYII-like proteins in which all binding sites are present are listed in Additional file 3: Table S3. We speculate that these proteins should be studied further with regard to their roles as active GLYII proteins.

We constructed another tree using the ML method, based on multiple sequence alignment of the full length of the putative GLYII proteins listed in Additional file 3: Table S3 from grape, *Arabidopsis*, rice, soybean and *M. truncatula* [8–10] (Fig. 2b). This tree was divided into two subgroups. VvGLYII-like1 is in the branch I, along with AtGLYII-5 which has been reported to be an active GLYII protein located in the mitochondria and to have a predominant metal center of Fe(III)Zn(II) but does not seem specifically to bind manganese [18]. VvGLYII-like2 is in branch II, along with AtGLYII-2 which is reported to be an active GLYII protein located in the cytosol and that has been shown to bind a mixture of Zn, Fe or Mn [21]. We speculate that these proteins in the two branches may be located in different places in the cell and have different metal dependences (Fig. 2b).

### Phylogenetic analysis of putative GLYIII proteins

It has been reported that both DJ-1and Hsp31 proteins contain the Glu-Cys-His catalytic triad. Among these, the glutamate and cysteine residues in the same locations in the two enzymes directly involved in catalysis are essential for GLYIII enzymatic activity. In contrast, histidine residues at different sequence and structural positions, are less important to enzyme activity [4, 5]. Previously reported DJ-1 protein sequences from *Arabidopsis*, rice, soybean and *M. truncatula* along with VvGLYIII-like proteins were aligned using ClustalW [9, 10, 56]. Incomplete sequences and proteins not containing the highly conserved glutamate and cysteine residues either in the N-terminal or C-terminal were discarded (Additional file 7: Figure S3). The remaining sequences were used for phylogenetic analysis. Using the MEGA 5.1 tool, a ML tree of the N-terminal and C-terminal DJ-1/PfpI domains from grape, *Arabidopsis*, rice soybean and *M. truncatula* along with the well characterised DJ-1/Hsp31 proteins from human, mouse, *Drosophila*, *C. elegans* and *E. coli* (Fig. 2c). This tree showed three major clades, I to III. Clade II contained all the DJ-1 proteins from the animal species. In Clade III, heat-inducible molecular chaperones (Hsp31) from *E. coli*, AtDJ-1D and OsDJ-1C have been shown in previous studies to be a primary GLYIII enzyme [4, 6, 7]. Thus VvGLYIII-like2 in this clade is speculated to be an important GLYIII enzyme worthy for further study. In Clade I, AtDJ-1a and AtDJ-1b exhibited weak specificity towards MG but higher specificity towards glyoxal and AtDJ-1a was involved in the oxidative stress response [23, 57]. Thus, we speculate that VvGLYIII-lik1 and VvGLYIII-lik3 in Clade I may exhibit weak glyoxalase activities or have different functions from glyoxalase.

### Expression profiles of grape glyoxalase genes in different tissues and under downy mildew stress

**Fig. 3:**
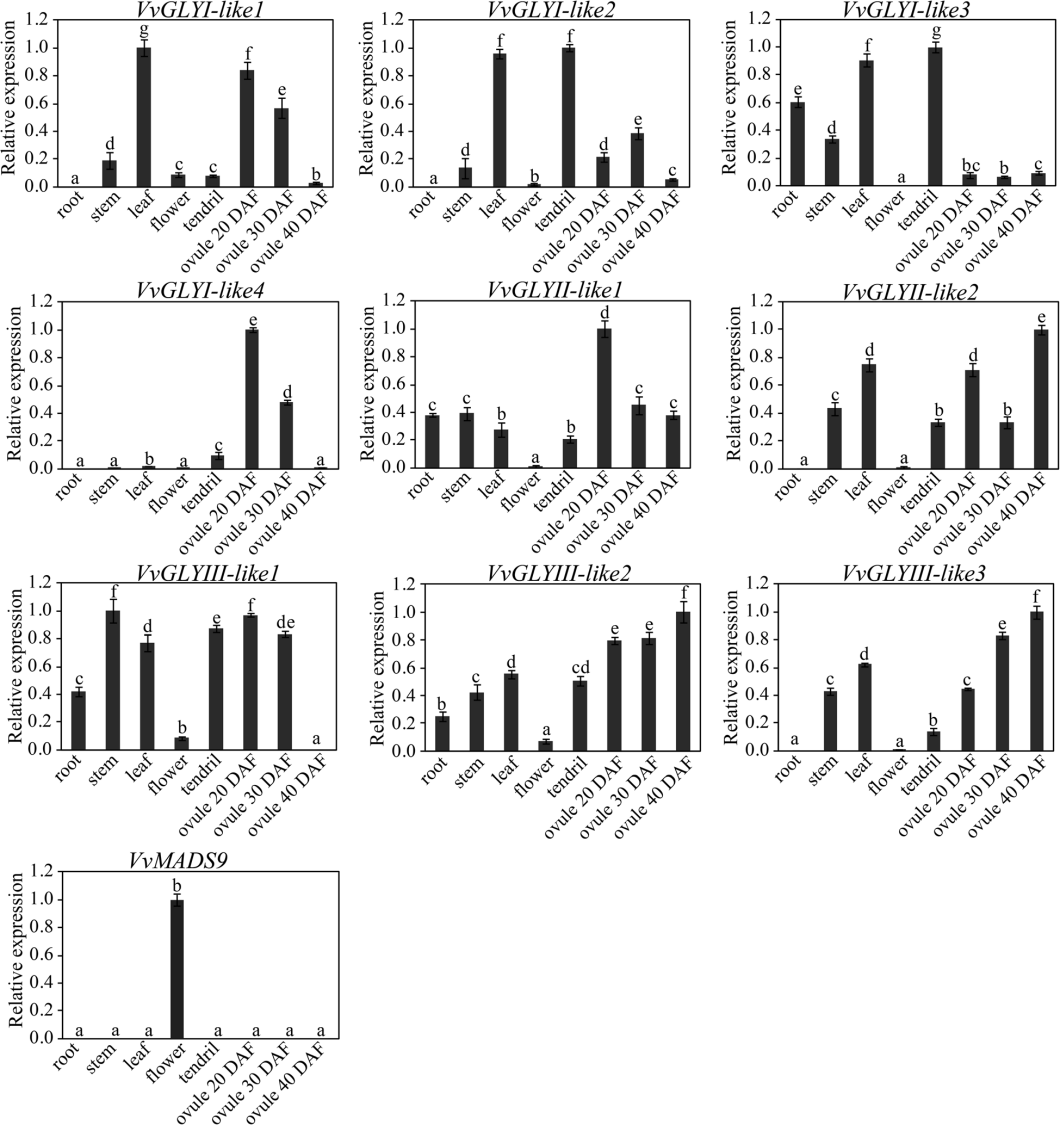
Expression analysis of grape glyoxalase genes in different tissues. All qRT-PCR experiments were carried out with three biological replications, and three technical repeats. The highest expression level was set at 1. Duncan’s multiple range test (*P* ≤ 0.05) is used to calculate the significant differences showing by different letters. Standard deviations are shown by error bars

**Fig. 4:**
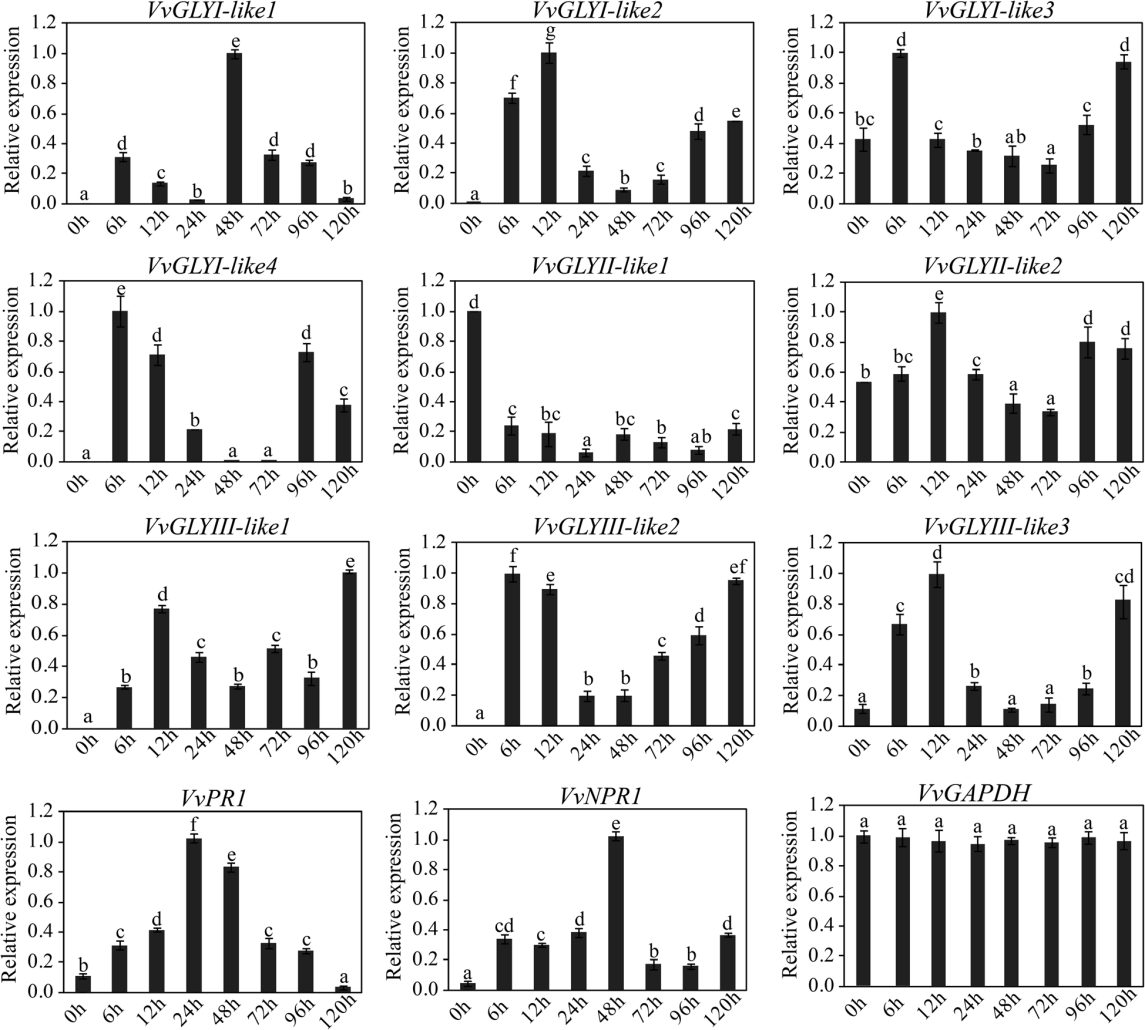
Expression analysis of grape glyoxalase genes under downy mildew stress. Eight time points after downy mildew inoculation are used in the analysis. The qRT-PCR experiments are conducted with three biological replicates and three technical repeats. The highest expression level was set at 1. Standard deviations are shown by error bars and Duncan’s multiple range test (*P* ≤ 0.05) was used to calculate the significant differences indicated by different letters

To investigate the expression of glyoxalase genes in the different tissues of grape, we analysed the expressions of glyoxalase genes in shoots, stems, leaves, flowers, tendrils and ovules at 20/30 and 40 days after flowering (DAF) in *V. vinifera* L. cv. ‘Thompson seedless’ by qRT-PCR. *VvMADS9* which is specifically expressed in flowers, served as the positive control [58, 59]. Based on the expression profiles, all the glyoxalase genes were expressed in ovules and most had higher expressions in stems, leaves, tendrils, but lower expressions in flowers. However, some differences were noted. For instance, *VvGLYI-4* had lower expressions in roots, stems, leaves than in tendrils and ovules (Fig. 3). With confirmation of the expressions of glyoxalase-like genes in grape, *PR1* and *NPR1* (involved in the defence response) and *VvGAPHD* (a housekeeping gene) were evaluated by qRT-PCR following infection with *P. viticola* (Fig. 4). There were no significant differences in the expressions of *VvGAPHD* after infection. This indicates our methods are reliable. The expressions of *PR1* and *NPR1* were found to increase 24–48 h after inoculation. However, *VvGLYI-like2, VvGLYI-like3*, *VvGLYI-like4*, *VvGLYII-like2* and all three *VvG*LY*YIII-like* genes were first expressed at 6–12 h, expression then decreased but increased again at 96–120 h, while *VvGLYII-like1* was down-regulated. Meanwhile, *VvGLYI-like1* was expressed most strongly at 48 h but like *PR1* and *NPR1*, expression then decreased. It is speculated that *VvGLYI-like1*could play an important role in grape’s defence response to *P. viticola* (Fig. 4)*.* The expression patterns of the various glyoxalase members are different. This indicates the various glyoxalase members play different roles in grape downy mildew stress modulation pathway.

## Discussion

### Identification of glyoxalase genes in plants

Genome-wide analysis of the glyoxalase enzymes have been carried out previously in rice, *Arabidopsis*, soybean and *M. truncatula* [8–10]. However, these families have not been studied in grape. Previous studies on GLYIs and GlYIIs in soybean and *M. truncatula* based on the presence of conserved metal ion binding sites and substrate binding sites to determine whether a protein sequence had enzyme activity [9, 10]. However, the screening criterias used in these studies were not comprehensive. Jain et al. screened GLYI-like gene sequences in *Arabidopsis* and determined they contained not only metal binding sites, but also GSH binding sites, active sites and dimer interfaces [11]. Limphong et al. characterised GlYIIs in *Arabidopsis* and identified 14 amino acids that are important for substrate binding, metal binding and catalytic activity [49]. Using the new screening criteria, the present study identifies four GLYI-like proteins and two GLYII-like proteins in grape, 10 GLYI-like proteins and three GLYII-like proteins in soybean and five GLYI-like proteins and two GLYII-like proteins in *M. truncatula*. The number of previously reported proteins in full families and putative active members of all five plant species are listed in Additional file 8: Table S5, including the length of their genomes [60–64]. Reports on the structural properties of plant GLYIII proteins are few, with structure reported only for *Arabidopsis* AtDJ-1d protein, the conserved binding site was Glu-Cys-His triad [6]. Similarly, OsDJ-1C, a rice DJ-1 protein, generated through homology modelling studies using AtDJ-1d as a template, also had the conserved three-dimensional structure [7]. These glyoxalase families display wide differences in their sequences which might lead to diversity in their biochemical functions. However, at this stage, little is known about plant DJ-1 proteins.

### Predicted metal ion dependence properties of glyoxalases

There are two types of metal dependency of GLYI proteins, Zn^2+^ and non-Zn^2+^ (mainly Ni^2+^). Regardless of the metal activation, both types have four conserved metal-binding residues (H/QEH/QE) [13, 57, 65]. Interestingly, metal dependence specificity of predicted GLYI proteins can easily be identified based on the length and sequence of proteins. The amino acid length of Zn^2+^-activated GLYI proteins is greater than of Ni^2+^-activated GLYI proteins and there is also a unique region in the sequence of Zn^2+^-activated proteins [11, 13, 56]. According to these criteria, in this study, three out of the four VvGLYI-like proteins are likely to be Ni^2+^-dependent and VvGLYI-like2 was speculated to be Zn^2+^-dependent. The same pattern of Ni^2+^-dependence dominance has been found in other plant species, two out of three *Arabidopsis* GLYI proteins are Ni^2+^-activated [11]. In addition, according to this study seven out of 10 soybean GLYI-like proteins and four out of five *M. truncatula* GLYI proteins are predicted to be Ni^2+^-activated (Additional file 1: Table S1). Moreover, previous reports have detected the presence of two GLYI domains in a single protein from *Saccharomyces cerevisiae*, *Plasmodium falciparum* and *O. sativa* [13, 66, 67]. Two domains could form two active sites in a single protein and both active sites were found to play roles in *P. falciparum* but one of the active sites was found to have no function in *O. sativa* [13, 67]. Here, VvGLYI-like1 and VvGLYI-like4 had two conserved glyoxalase domains (PF00903) (Fig. 1). They also possess four conserved metal binding residues in the N- terminal and three in the C- terminal (Additional file 5: Figure S1 and Additional file 9: Figure S4). That is the C- terminal domain of VvGLYIlike-1 and VvGLYI-like4 may have no function, as in rice. Similarly, GLYII enzymes also require divalent cations for their activity. GLYII proteins of plants, usually possess a binuclear metal binding center [16, 18, 20, 23]. AtGLYII-5 is reported to have a predominant metal center of Fe(III)Zn(II) but does not appear to bind Mn specifically [18]. However, AtGLYII-2 has been shown to bind a mixture of Zn, Fe or Mn and it also exhibits positive cooperativity in metal binding [21]. The gene dependence on metal ions still needs to be proved by experiments. We just predicted the metal dependence, providing some ideas for future research.

Previous characterisations of GLYIII activity in all species display a metal ion-independent nature [5, 6]. However, Hsp31 and human DJ-1 as glyoxalases reveal the existence of the essential catalytic residues, glutamic acid and cysteine, together with a histidine residue that assists catalysis [4, 5]. However, the histidine positioning in DJ-1 and Hsp31 are different. In the present study, both domains in the N-terminal and C-terminal of VvGLYIII-like2 resembled Hsp31, AtDJ-1d and OsDJ-1C in that the cysteine and histidine were contiguous (Additional file 7: Figure S3). These proteins are reported to be important detoxification GLYIII enzymes in *Arabidopsis*, rice and *E. coli* [6, 7]. Thus we predict that VvGLYIII-like2 could have the same functions as these and might play important roles as a GLYIII in grape. In contrast, the N-terminal of VvGLYIIIlike-3 resembled that of *Drosophila* in which histidine is replaced by tyrosine but the histidine in the C-terminal of VvGLYIII-like3 was replaced by phenylalanine [68]. However, the N-terminal and C-terminal of VvGLYIII-like1 are in the same as human DJ-1protein (Additional file 7: Figure S3). Although the catalytic core is similar, catalytic activity was quite variable among the members of the GLYIII family and this suggests a variety of function for the members in this family.

### Different expression patterns of glyoxalase genes in response to stress

The role of the glyoxalase system in resistance to multiple biotic and abiotic stresses has been well characterised in some studies. Recently, it has been reported that the glyoxalase pathway of rice not only increases tolerance to salinity, drought and extreme temperatures but also reduces damage from sheath blight fungus (*Rhizoctonia solani*) [69]. Moreover, some glyoxalase members of soybean such as *GmGLYI*-6/9/20 and *GmGLYII*-6/10 show special expression patterns in response to various pathogenic infection [9]. However, whether glyoxalase proteins participate in downy mildew response is unknown. In this study, we found that after downy mildew inoculation, most glyoxalase genes had two periods of high expression at 6–12 h and at 96–120 h. In particular, *VvGLYII-like1* had a down-regulated expression pattern after downy mildew inoculation. After fungal infection, intercellular MG levels are significantly enhanced in susceptible genotypes, so high GLYI activity is needed [5]. Nevertheless, a rice *GLYI* gene was found to be down-regulated after infection with *Xanthomonas oryzae* pv. *oryzae* or with *Pyricularia grisea* [38]. Like *PR1* and *NPR1*, *VvGLYI-like1* was highly expressed 48 h after inoculation. Thus, *VvGLYI-like1* may play an important role in the defence response to downy mildew. Whether the other glyoxalase-like genes are involved in defence responses still needs to be clarified. The infection process of *P. viticola* is complex and takes a long time, the expression levels of genes during this process are very variable. Further studies are required to determine if over expression of glyoxalase genes could offer direct protection to plants from pathogens.

Moreover, the high identity of glyoxalase genes between grape and *Arabidopsis* at the protein level indicates that glyoxalase genes may have similar conserved functions to their Arabidopsis orthologue genes (Additional file 10: Table S6). As with the grape glyoxalase-like genes, *AtGLYI-2*, *AtGLYI-3*, *AtGLYI-6*, *AtGLYII-2* and *AtGLYII-5* are highly expressed at all the stages of development [8]. The frequency of ESTs or cDNAs available in different databases has been considered as a useful tool for preliminary analysis of gene expression [70]. The search for ESTs was performed by comparing the grape glyoxalase cDNA sequences against EST sequences available at NCBI in BLASTN searches with the search parameters: maximum identity, > 95%; length, > 300 bp; and E value, < 10^− 10^ [71]. The ESTs were identified for all of the GLY genes, indicating they are expressed. However, the frequency of ESTs was low, indicating that these are expressed at very low levels. The matched EST sequences were derived from various grape tissues or libraries such as root, leaf, berry, seed, bud, flower, pericarp, shoot, clusters and stem, indicating the differential expression of VvGLY-likes in grape tissues (Additional file 11: Table S7).

## Conclusions

This study identifies four GLYI-like, two GLYII-like and three GLYIII-like glyoxalase proteins in grape. All these newly-identified members were further analysed for their homology, essential binding sites for catalytic activity, structural properties, expressions in different tissues and expressions under stress from downy mildew infection. This study lays a foundation for researching the roles of *GLY* genes of grape in response to downy mildew stress. Most glyoxalase genes were highly expressed at 6–12 h and 96–120 h post inoculation but *VvGLYII-like1* expression was down-regulated. In particular, *VvGLYI-like1* was highly expressed 48 h after inoculation and, like *PR1* and *NPR1*, might be involved in defence response to downy mildew. Despite many recent advances in functional studies of glyoxalase genes, the biological functions of most glyoxalase genes in physiological and developmental processes or plant defence still require elucidation. The bioinformatic analysis and expression patterns of the grape glyoxalase gene families conducted in our study provide an overview of the composition and expression of glyoxalase genes in grapevine that will facilitate selection of candidate genes for cloning and for further functional characterisation.

## Methods

### Identification of glyoxalase genes in grape

To identify the GLYI, GLYII and GLYIII genes in grape, the Hidden Markov Model (HMM) profiles of the glyoxalase domain (PF00903), metallo-beta-lactamase domain (PF00753) and DJ-1/PfpI (PF01965) were downloaded from the Pfam database (http://pfam.xfam.org/). They were then used as queries to search the Grape Genome Database (12X) (http://www.genoscope.cns.fr/externe/GenomeBrowser/Vitis/) using HMMER3.1 with default E-values (< 1.0) [48]. All putative GLYI, GLYII and GLYIII genes were manually confirmed by searching the NCBI (https://www.ncbi.nlm.nih.gov/) and the Pfam program again with score value ≥100 and e-value less than 10^− 30^. To find the possibly active GLYI proteins, each of the identified VvGLYI-like protein sequences along with putative GLYI proteins in rice, *Arabidopsis*, soybean and *M. truncatula* was analysed using BLASTP at the score value ≥100 and E value ≤0. Proteins with conserved binding sites, active sites, metal binding sites, GSH binging sites and dimer interfaces were assumed to be GLYI-like. The predicted proteins are named: prefix “Vv” for *Vitis vinifera*, followed by GLYI-like, GLYII-like or GLYIII-like, and lastly the Arabic numbers (e.g. 1, 2, 3) based on their locations on the chromosome.

### Exon-intron and protein structure analysis

The exon and intron of the *glyoxalase-like* genes were identified according to the Grape Genome Browser. Diagrams were drawn using the online tool GSDS 2.0 (http://gsds.cbi.pku.edu.cn) [72]. Coding sequences (CDs) and untranslated region (UTR) sequences of glyoxalase genes are provided in Additional files 16. All the newly predicted grape glyoxalase-like proteins were analysed using Pfam (http://pfam.xfam.org) to reveal the position of domains.

### Multiple sequence alignment and phylogenetic analyses

Two unrooted phylogenetic trees were constructed respectively with amino acid sequences of N-terminal lactoylglutathione lyase domain (PF00903) in GLYI-like and full length of GLYII-like proteins from grape *Arabidopsis*, rice, soybean, *M. truncatula* using MEGA 5.1 with the Maximum Likelihood method and 1000 bootstrap replicates [73, 74]. In addition, N-terminal and C-terminal DJ-1/PfpI (PF01965) domain from grape *Arabidopsis*, rice, *M. truncatula* along with DJ-1/PfpI domain from human, mouse, *Drosophila*, *C. elegans* and Hsp31 from *E. coli* were used to construct another phylogenetic tree using the same method and the sequences are shown in Additional files 13, 14 and 15 respectively. Multiple sequence alignment was carried out using ClustalW [75] and visualized with Jalview [76].

### Expression of grape glyoxalase genes in different tissues and under downy mildew stress

‘Thompson Seedless’ (*Vitis vinifera* L.) was grown in the vineyard of Northwest A&F University, Yangling, Shaanxi, China and grapevines were managed according to local standards. Samples of roots, stems, leaves, flowers, tendrils and ovules were collected. Ovules were collected 20 DAF (small globular embryo), 30 DAF (globular embryo) and 40 DAF (aborted embryo). For the downy mildew treatment, downy mildew (*Plasmopara viticola*) infected leaves were collected from the vineyard. Sporangia were isolated using vacuum aspiration [77]. Detached healthy leaves of ‘Thomson seedless’ were obtained from the same position on the shoot (fifth to sixth unfolded leaf). These were surface sterilized with 70% (vol/vol) ethanol and then rinsed in deionised water. The abaxial leaf surfaces were inoculated with 100 μl droplets of an aqueous suspension of sporangia (2 × 10^6^ sporangia ml^− 1^). The control plants were neither inoculated nor elicitor-treated. The petioles were then covered with a piece of wet cotton, incubated in the dark at 25 °C and > 95% relative humidity (RH) [78]. Inoculated leaves were then collected after 0, 6, 12, 24, 48, 72, 96 and 120 h, these were immediately immersed in liquid nitrogen and stored at − 80 °C pending analysis. All the plant materials were replicated three times.

### RNA isolation, qRT-PCR and statistical analysis

The EZNA Plant RNA Kit (Omega, Guangzhou, China) was used to extract the total RNA following the manufacturer’s instructions. For qRT-PCR (Quantitative Real-time PCR) analysis, the first-strand cDNA was synthesised using PrimeScript RTase (Takara, Dalian, China) according to the manufacturer’s instructions. Primers were designed by Primer Premier 5.0 (Premier, Canada) and these are listed in Additional file 12: Table S8. The qRT-PCR was carried out using SYBR green (Takara, Dalian, China) with the IQ5 real time PCR system (Bio-Rad, Hercules, CA, USA). Grape *Actin 7* (Accession no. XM_002282480) was used as an internal control. *VvMADS9* (Accession no. NM_001280946), *VvPR1* (Accession no. XM_002273752) and *VvNPR1* (Accession no. XM_002281439) were used as a positive control. Three biological repeats were used and each reaction was carried out three times. The normalised expression method (2^-△△C(t)^ method) was used to analyse the relative expression levels with the highest level of expression set as 1. Statistical analyses were conducted using SPSS 18.0 Software (Chicago, IL) and Excel. Statistically significant analyses were based on Duncan’s biological repeats and each reaction is reported as a mean with standard deviations (SDs) presented as error bars.

## Additional files

Additional file 1: **Table S1.** Essential amino acids and metal ion dependency analysis of all putative GLYI proteins from *Vitis vinifera*, *Arabidopsis*, *Oryza sativa*, *Glycine max* and *Medicago truncatula*. (DOCX 15 kb)

Additional file 2: **Table S2.** Conserved binding sites analysis of genes previously reported as GLYI proteins of *Arabidopsis*, rice, soybean and *Medicago truncatula* that are not likely to be GLYs. (DOC 94 kb)

Additional file 3: **Table S3.** Conserved binding sites analysis of all putative GLYII proteins from *Vitis vinifera*, *Arabidopsis*, *Oryza sativa*, *Glycine max* and *Medicago truncatula*. (DOCX 14 kb)

Additional file 4: **Table S4.** Conserved binding sites analysis of genes previously reported as GLYII proteins of *Arabidopsis*, rice, soybean and *Medicago truncatula* that are not likely to be GLYs. (DOCX 16 kb)

Additional file 5: **Figure S1.** Multiple Sequence alignments of GLYI domains. N-terminal GLYI domains of proteins listed in Additional file 1: Table S1, along with a Gly I from human (*Homo sapiens*, Accession No: AB209801), were aligned using ClustalW and edited using the Jalview program. Four conserved residues (H/E/H/E) for metal binding are shown with black boxes and specific regions for Zn^2+^-dependence are in pink boxes. (DOCX 759 kb)

Additional file 6: **Figure S2.** Sequence alignments of full length of GLYII proteins. Multiple sequence alignments were conducted with full length protein sequences listed in Additional file 3: Table S3 along with GlyII of human (*Homo sapiens*, Accession No: NP_005317) and a known GLYII protein from *Brassica juncea* (*B. juncea,* Accession No: AY185202). The conserved active motif G/CHT is indicated in black boxes. The conserved metal binding sites are marked with “#” and the GSH binding sites are marked with red stars. (DOCX 781 kb)

Additional file 7: **Figure S3.** Sequence alignment of the N-terminal and C-terminal DJ-1/PfpI domains of GLYIII-like proteins. Both the N-terminal and C-terminal DJ-1/PfpI domains of VvGLYIII-like proteins were aligned with other well characterized DJ-1/PfpI superfamily proteins from human, mouse, *Drosophila*, *Caenorhabditis elegans*, *Escherichia coli* and orthologs from *Arabidopsis,* rice, soybean and *Medicago truncatula*. Three residues relate to the activity are marked with black boxes. Proteins without the cysteine in the N-terminal or C-terminal are marked with red boxes respectively. (DOCX 1457 kb)

Additional file 8: **Table S5.** The number of previously reported glyoxalase genes and glyoxalase genes with the critical conserved binding sites in grape, rice, *Arabidopsis*, soybean and *Medicago truncatula*. (DOCX 17 kb)

Additional file 9: **Figure S4.** Multiple sequence alignment of C-terminal GLYI domain of VvGLYI-like1 and VvGLYI-like4. C-terminal GLYI domains of VvGLYI-like1 and VvGLYI-like4 were aligned with the C-terminal GLYI domain of OsGLYI-11 and the N-terminal GLYI domain of AtGLYI-2 using ClustalW and then edited by the Jalview program. All four conserved metal binding sites are shown in black boxes. (DOCX 247 kb)

Additional file 10: **Table S6.** Percent Identity Matrix of GLY-like families bweteen grape and *Arabidopsis*. (DOCX 23 kb)

Additional file 11: **Table S7.** Expression analysis of VvGLY-like genes in the ESTs database. (DOCX 14 kb)

Additional file 12: **Table S8.** Primers used in expression analysis of glyoxalase-like gene families in grape. (DOCX 18 kb)

Additional file 13: Amino acid sequences of lactoylglutathione lyase domains and full length protein sequences of GLYIs used for phylogenetic analysis and multiple sequence alignment. (DOCX 22 kb)

Additional file 14: Amino acid sequences of full length putative GLYIIs used for phylogenetic analysis and multiple sequence alignment. (DOCX 17 kb)

Additional file 15: Amino acid sequences of DJ-1/PfpI domains and full length sequences of DJ-1 proteins used for phylogenetic analysis. (DOCX 25 kb)

Additional file 16: Other sequences and their accession numbers used in this paper. (DOCX 58 kb)

## Abbreviations

CDS: Coding sequence
DAF: Day after flowering
GLYI: Glyoxalase I
GLYII: Glyoxalase II
GLYIII: Glyoxalase III
GSDS: Gene Structure Display Server
GSH: reduced glutathione
HMM: Hidden Markov Model
HTA: Hemithioacetal
Mb: Mega base
MG: Methylglyoxal
ML: Maximum Likelihood
qRT-PCR: Quantitative Real-time PCR
RH: Relative humidity
SLG: S-D-lactoyl-glutathione
UTR: Untranslated region
