# Supplementary material for: Genome-wide analysis of glyoxalase-like gene families in grape (Vitis vinifera L.) and their expression profiling in response to downy mildew infection
Source: BMC Genomics. 2019 May 9;20:362. doi: 10.1186/s12864-019-5733-y (PMC6509763; doi:10.1186/s12864-019-5733-y)
Supplement: Supplementary file 1 — Table S1. Essential amino acids and metal ion dependency analysis of all putative GLYI proteins from Vitis vinifera, Arabidopsis, Oryza sativa, Glycine max and Medicago truncatula. (DOCX 15 kb) [file 12864_2019_5733_MOESM1_ESM.docx]

**Additional file 1 Table S1.** Essential amino acids and metal ion dependency analysis of all putative GLYI proteins from *Vitis vinifera*, *Arabidopsis*, *Oryza sativa*, *Glycine max* and *Medicago truncatula*

| Putative GLYI Protein | Active site | Metal binding site | GSH binding site | Dimer interface | Length of GLYI domain(aa) | Metal ion dependency | Essential amino acids |
| --- | --- | --- | --- | --- | --- | --- | --- |
| VvGLYI-like1 | √ | √ | √ | √ | 121 | Ni | Present |
| VvGLYI-like2 | √ | √ | √ | √ | 151 | Zn | Present |
| VvGLYI-like3 | √ | √ | √ | √ | 121 | Ni | Present |
| VvGLYI-like4 | √ | √ | √ | √ | 121 | Ni | Present |
| AtGLYI-2 | √ | √ | √ | √ | 151 | Zn | Present |
| AtGLYI-3 | √ | √ | √ | √ | 121 | Ni | Present |
| AtGLYI-6 | √ | √ | √ | √ | 121 | Ni | Present |
| OsGLYI-2 | √ | √ | √ | √ | 121 | Ni | Present |
| OsGLYI-7 | √ | √ | √ | √ | 121 | Ni | Present |
| OsGLYI-8 | √ | √ | √ | √ | 151 | Zn | Present |
| OsGLYI-11 | √ | √ | √ | √ | 121 | Ni | Present |
| GmGLYI-1 | √ | √ | √ | √ | 123 | Ni | Present |
| GmGLYI-3 | √ | √ | √ | √ | 121 | Ni | Present |
| GmGLYI-4 | √ | √ | √ | √ | 121 | Ni | Present |
| GmGLYI-8 | √ | √ | √ | √ | 121 | Ni | Present |
| GmGLYI-10 | √ | √ | √ | √ | 121 | Ni | Present |
| GmGLYI-11 | √ | √ | √ | √ | 121 | Ni | Present |
| GmGLYI-14 | √ | √ | √ | √ | 175 | Zn | Present |
| GmGLYI-15 | √ | √ | √ | √ | 151 | Zn | Present |
| GmGLYI-16 | √ | √ | √ | √ | 151 | Zn | Present |
| GmGLYI-21 | √ | √ | √ | √ | 121 | Ni | Present |
| MtGLYI-4 | √ | √ | √ | √ | 121 | Ni | Present |
| MtGLYI-7 | √ | √ | √ | √ | 121 | Ni | Present |
| MtGLYI-10 | √ | √ | √ | √ | 151 | Zn | Present |
| MtGLYI-22 | √ | √ | √ | √ | 121 | Ni | Present |
| MtGLYI-24 | √ | √ | √ | √ | 121 | Ni | Present |

aa, amino acid; The proteins from *A. thaliana* were previously reported in reference [8]; The proteins from *G. max* were previously reported in reference [9]; The proteins from *M. truncatula* were previously reported in reference [10].
