# Supplementary material for: Genome-wide analysis of glyoxalase-like gene families in grape (Vitis vinifera L.) and their expression profiling in response to downy mildew infection
Source: BMC Genomics. 2019 May 9;20:362. doi: 10.1186/s12864-019-5733-y (PMC6509763; doi:10.1186/s12864-019-5733-y)
Supplement: Supplementary file 2 — Table S2. Conserved binding sites analysis of genes previously reported as GLYI proteins of Arabidopsis, rice, soybean and Medicago truncatula that are not likely to be GLYs. (DOC 94 kb) [file 12864_2019_5733_MOESM2_ESM.doc]

**Additional file 2 Table S2.** Conserved binding sites analysis of genes previously reported as GLYI proteins of *Arabidopsis*, rice, soybean and *Medicago truncatula* that are not likely to be GLYs

| Putative GLYI Protein | Active site | Metal binding site | GSH binding site | Dimer interface |
| --- | --- | --- | --- | --- |
| AtGLYI-1^a^ | － | － | － | － |
| AtGLYI-4^a^ | － | √ | － | √ |
| AtGLYI-5^a^ | √ | － | － | － |
| AtGLYI-7^a^ | － | √ | － | √ |
| AtGLYI-8^a^ | － | √ | － | √ |
| AtGLYI-9^a^ | － | √ | － | √ |
| AtGLYI-10^a^ | √ | － | － | － |
| AtGLYI-11^a^ | － | － | － | √ |
| OsGLYI-1^a^ | － | √ | － | √ |
| OsGLYI-3^a^ | √ | √ | － | － |
| OsGLYI-4^a^ | － | √ | － | √ |
| OsGLYI-5^a^ | － | √ | － | √ |
| OsGLYI-6^a^ | － | √ | － | √ |
| OsGLYI-9^a^ | － | √ | － | √ |
| OsGLYI-10^a^ | － | √ | － | √ |
| GmGLYI-2^b^ | － | √ | － | √ |
| GmGLYI-5^b^ | √ | √ | － | － |
| GmGLYI-6^b^ | － | √ | － | √ |
| GmGLYI-7^b^ | √ | － | √ | √ |
| GmGLYI-9^b^ | － | √ | － | √ |
| GmGLYI-12^b^ | － | － | － | － |
| GmGLYI-13^b^ | － | √ | － | √ |
| GmGLYI-17^b^ | － | √ | － | √ |
| GmGLYI-18^b^ | － | √ | － | √ |
| GmGLYI-19^b^ | － | √ | － | √ |
| GmGLYI-20^b^ | － | √ | － | √ |
| GmGLYI-22^b^ | － | √ | － | √ |
| GmGLYI-23^b^ | － | √ | － | √ |
| GmGLYI-24^b^ | － | √ | － | √ |
| MtGLYI-1^c^ | 4-hydroxyphenylpyruvate dioxygenase | | | |
| MtGLYI-2^c^ | － | √ | － | √ |
| MtGLYI-3^c^ | － | √ | － | √ |
| MtGLYI-5^c^ | － | √ | － | √ |
| MtGLYI-6^c^ | － | √ | － | √ |
| MtGLYI-8^c^ | － | √ | － | √ |
| MtGLYI-9^c^ | √ | － | √ | － |
| MtGLYI-11^c^ | － | √ | － | √ |
| MtGLYI-12^c^ | － | √ | － | √ |
| MtGLYI-13^c^ | － | √ | － | √ |
| MtGLYI-14^c^ | √ | － | √ | √ |
| MtGLYI-15^c^ | √ | － | √ | √ |
| MtGLYI-16^c^ | － | √ | － | √ |
| MtGLYI-17^c^ | － | √ | － | √ |
| MtGLYI-18^c^ | － | － | － | － |
| MtGLYI-19^c^ | － | √ | － | √ |
| MtGLYI-20^c^ | 4-hydroxyphenylpyruvate dioxygenase | | | |
| MtGLYI-21^c^ | 4-hydroxyphenylpyruvate dioxygenase | | | |
| MtGLYI-23^c^ | － | √ | － | √ |
| MtGLYI-25^c^ | － | √ | － | √ |
| MtGLYI-26^c^ | － | － | － | － |
| MtGLYI-27^c^ | 2-hydroxy-6-oxo-6-phenylhexa-2,4-dienoate hydrolase | | | |
| MtGLYI-28^c^ | enoyl-CoA hydratase/isomerase | | | |
| MtGLYI-29^c^ | √ | √ | － | － |

^a^ This protein was previously reported as GLYI protein in reference [8]

^b^ This protein was previously reported as GLYI protein in reference [9]

^c^ This protein was previously reported as GLYI protein in reference [10]
