# Supplementary material for: Genome-wide analysis of glyoxalase-like gene families in grape (Vitis vinifera L.) and their expression profiling in response to downy mildew infection
Source: BMC Genomics. 2019 May 9;20:362. doi: 10.1186/s12864-019-5733-y (PMC6509763; doi:10.1186/s12864-019-5733-y)
Supplement: Supplementary file 3 — Table S3. Conserved binding sites analysis of all putative GLYII proteins from Vitis vinifera, Arabidopsis, Oryza sativa, Glycine max and Medicago truncatula. (DOCX 14 kb) [file 12864_2019_5733_MOESM3_ESM.docx]

**Additional file 3 Table S3.** Conserved binding sites analysis of all putative GLYII proteins from *Vitis vinifera*, *Arabidopsis*, *Oryza sativa*, *Glycine max* and *Medicago truncatula*

| Putative Grape GLYII Protein | Active site | Metal binding site | GSH binding site | Essential amino acids |
| --- | --- | --- | --- | --- |
| VvGLYII-like1 | √ | √ | √ | Present |
| VvGLYII-like2 | √ | √ | √ | Present |
| AtGLYII-2 | √ | √ | √ | Present |
| AtGLYII-4 | √ | √ | √ | Present |
| AtGLYII-5 | √ | √ | √ | Present |
| OsGLYII-2 | √ | √ | √ | Present |
| OsGLYII-3 | √ | √ | √ | Present |
| GmGLYII-7 | √ | √ | √ | Present |
| GmGLYII-8 | √ | √ | √ | Present |
| GmGLYII-9 | √ | √ | √ | Present |
| MtGLYII-7 | √ | √ | √ | Present |
| MtGLYII-12 | √ | √ | √ | Present |

The proteins from *A. thaliana* were previously reported in reference [8]; The proteins from *G. max* were previously reported in reference [9]; The proteins from *M. truncatula* were previously reported in reference [10]
