# Supplementary material for: Genome-wide analysis of glyoxalase-like gene families in grape (Vitis vinifera L.) and their expression profiling in response to downy mildew infection
Source: BMC Genomics. 2019 May 9;20:362. doi: 10.1186/s12864-019-5733-y (PMC6509763; doi:10.1186/s12864-019-5733-y)
Supplement: Supplementary file 4 — Table S4. Conserved binding sites analysis of genes previously reported as GLYII proteins of Arabidopsis, rice, soybean and Medicago truncatula that are not likely to be GLYs. (DOCX 16 kb) [file 12864_2019_5733_MOESM4_ESM.docx]

**Additional file 4 Table S4.** Conserved binding sites analysis of genes previously reported as GLYII proteins of *Arabidopsis*, rice, soybean and *Medicago truncatula* that are not likely to be GLYs

| Putative Grape GLYII Protein | Active site | Metal binding site | GSH binding site |
| --- | --- | --- | --- |
|  |  |  |  |
|  |  |  |  |
| AtGLYII-1^a^ | √ | - | - |
| AtGLYII-3^a^ | ethylmalonic encephalopathy protein 1(ETHE1) | | |
| OsGLYII-1^a^ | ethylmalonic encephalopathy protein 1(ETHE1) | | |
| GmGLYII-1^b^ | √ | - | - |
| GmGLYII-2^b^ | √ | √ | - |
| GmGLYII-3^b^ | √ | √ | - |
| GmGLYII-4^b^ | √ | - | √ |
|  |  |  |  |
|  |  |  |  |
| GmGLYII-5^b^ | √ | - | √ |
| GmGLYII-6^b^ | ethylmalonic encephalopathy protein 1(ETHE1) | | |
| GmGLYII-10^b^ | √ | √ | - |
| GmGLYII-11^b^ | √ | - | - |
| GmGLYII-12^b^ | √ | √ | - |
| MtGLYII-1^c^ | - | - | - |
| MtGLYII-2^c^ | - | - | - |
| MtGLYII-3^c^ | √ | √ | - |
| MtGLYII-4^c^ | - | - | - |
| MtGLYII-5^c^ | - | - | - |
| MtGLYII-6^c^ | ethylmalonic encephalopathy protein 1(ETHE1) | | |
| MtGLYII-8^c^ | - | - | - |
| MtGLYII-9^c^ | RNA-metabolising metallo-beta-lactamase | | |
| MtGLYII-10^c^ | - | √ | - |
| MtGLYII-11^c^ | RNA-metabolising metallo-beta-lactamase | | |
| MtGLYII-13^c^ | - | - | - |
| MtGLYII-14^c^ | √ | √ | - |

^a^ This protein was previously reported as GLYII protein in reference [8]

^b^ This protein was previously reported as GLYII protein in reference [9]

^c^ This protein was previously reported as GLYII protein in reference [10]
