# Supplementary material for: Genome-wide analysis of glyoxalase-like gene families in grape (Vitis vinifera L.) and their expression profiling in response to downy mildew infection
Source: BMC Genomics. 2019 May 9;20:362. doi: 10.1186/s12864-019-5733-y (PMC6509763; doi:10.1186/s12864-019-5733-y)
Supplement: Supplementary file 5 — Figure S1. Multiple Sequence alignments of GLYI domains. N-terminal GLYI domains of proteins listed in Additional file 1: Table S1, along with a Gly I from human (Homo sapiens, Accession No: AB209801), were aligned using ClustalW and edited using the Jalview program. Four conserved residues (H/E/H/E) for metal binding are shown with black boxes and specific regions for Zn2+-dependence are in pink boxes. (DOCX 759 kb) [file 12864_2019_5733_MOESM5_ESM.docx]

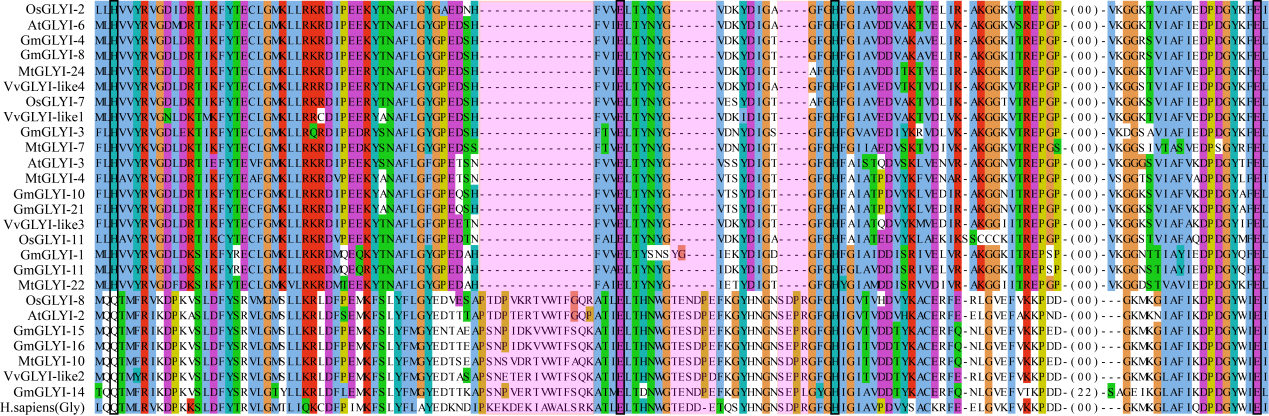


**Additional file 5: Figure S1. Multiple Sequence alignments of GLYI domains.** N-terminal GLYI domains of proteins listed in Additional file 1: Table S1, along with a Gly I from human (*Homo sapiens*, Accession No: AB209801), were aligned using ClustalW and edited using the Jalview program. Four conserved residues (H/E/H/E) for metal binding are shown with black boxes and specific regions for Zn^2+^-dependence are in pink boxes.
