# Supplementary material for: Genome-wide analysis of glyoxalase-like gene families in grape (Vitis vinifera L.) and their expression profiling in response to downy mildew infection
Source: BMC Genomics. 2019 May 9;20:362. doi: 10.1186/s12864-019-5733-y (PMC6509763; doi:10.1186/s12864-019-5733-y)
Supplement: Supplementary file 6 — Figure S2. Sequence alignments of full length of GLYII proteins. Multiple sequence alignments were conducted with full length protein sequences listed in Additional file 3: Table S3 along with GlyII of human (Homo sapiens, Accession No: NP_005317) and a known GLYII protein from Brassica juncea (B. juncea, Accession No: AY185202). The conserved active motif G/CHT is indicated in black boxes. The conserved metal binding sites are marked with “#” and the GSH binding sites are marked with red stars. (DOCX 781 kb) [file 12864_2019_5733_MOESM6_ESM.docx]

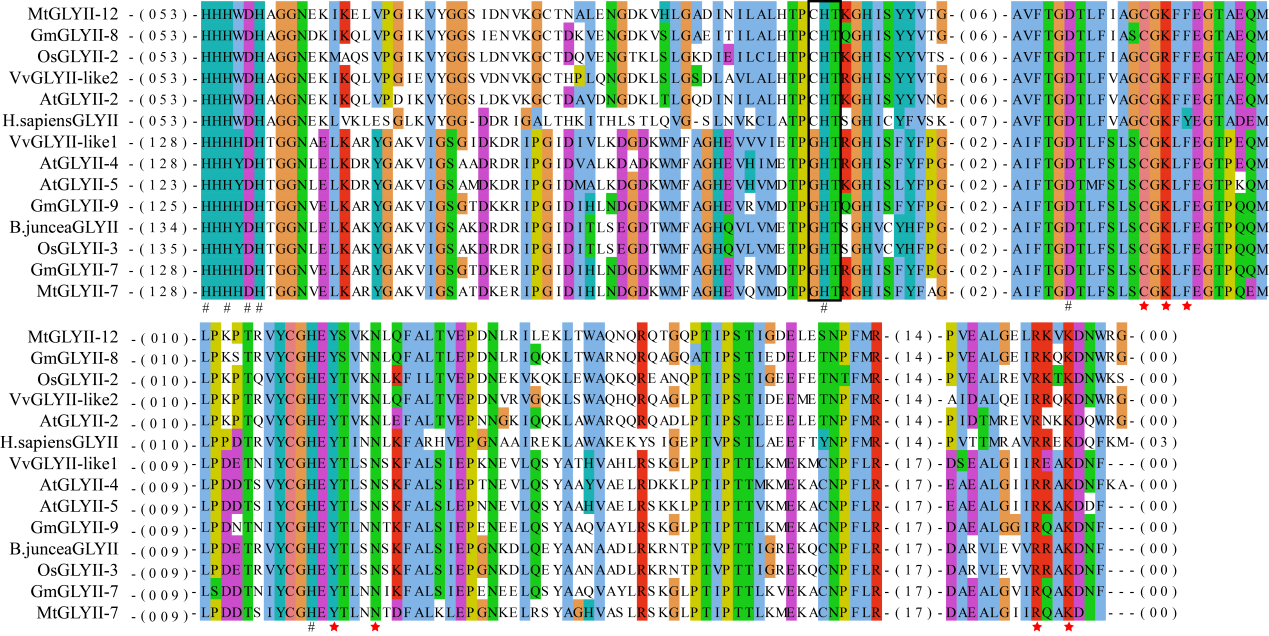


**Additional file 6: Figure S2. Sequence alignments of full length of GLYII proteins.** Multiple sequence alignments were conducted with full length protein sequences listed in Additional file 3: Table S3 along with GlyII of human (*Homo sapiens*, Accession No: NP_005317) and a known GLYII protein from *Brassica juncea* (*B. juncea,* Accession No: AY185202). The conserved activ motif G/CHT are indicated in black boxes. The conserved metal binding sites are marked with "#" and the GSH binding sites are marked with red stars.
