# Supplementary material for: Genome-wide analysis of glyoxalase-like gene families in grape (Vitis vinifera L.) and their expression profiling in response to downy mildew infection
Source: BMC Genomics. 2019 May 9;20:362. doi: 10.1186/s12864-019-5733-y (PMC6509763; doi:10.1186/s12864-019-5733-y)
Supplement: Supplementary file 7 — Figure S3. Sequence alignment of the N-terminal and C-terminal DJ-1/PfpI domains of GLYIII-like proteins. Both the N-terminal and C-terminal DJ-1/PfpI domains of VvGLYIII-like proteins were aligned with other well characterized DJ-1/PfpI superfamily proteins from human, mouse, Drosophila, Caenorhabditis elegans, Escherichia coli and orthologs from Arabidopsis, rice, soybean and Medicago truncatula. Three residues relate to the activity are marked with black boxes. Proteins without the cysteine in the N-terminal or C-terminal are marked with red boxes respectively. (DOCX 1457 kb) [file 12864_2019_5733_MOESM7_ESM.docx]

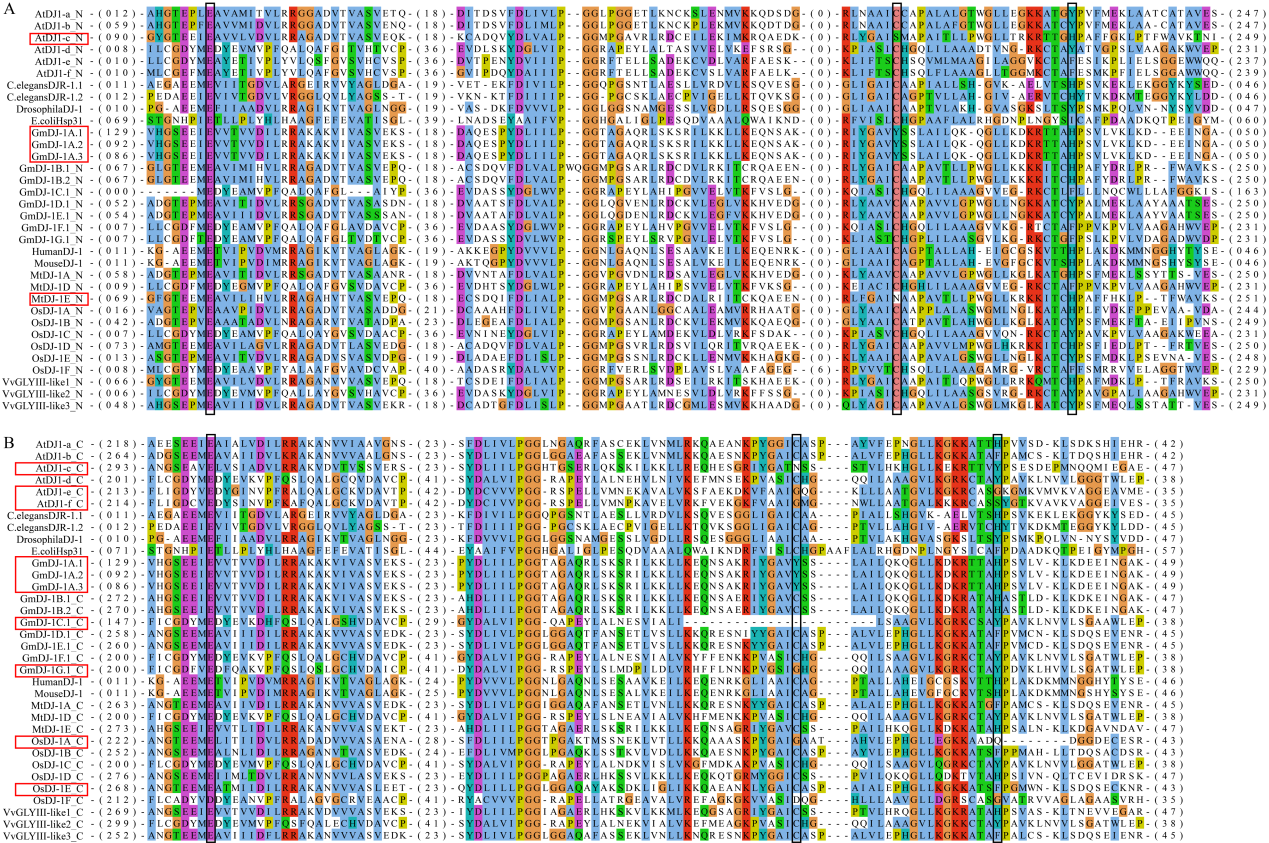


**Additional file 7: Figure S3. Sequence alignment of the N-terminal and C-terminal DJ-1/PfpI domains of GLYIII-like proteins.** Both the N-terminal and C-terminal DJ-1/PfpI domains of VvGLYIII-like proteins were aligned with other well characterized DJ-1/PfpI superfamily proteins from human, mouse, *Drosophila*, *Caenorhabditis elegans*, *Escherichia coli* and orthologs from *Arabidopsis,* rice, soybean and *Medicago truncatula*. Three residues relate to the activity are marked with black boxes. Proteins without the cysteine in the N-terminal or C-terminal are marked with red boxes respectively.
