# Supplementary material for: Genome-wide analysis of glyoxalase-like gene families in grape (Vitis vinifera L.) and their expression profiling in response to downy mildew infection
Source: BMC Genomics. 2019 May 9;20:362. doi: 10.1186/s12864-019-5733-y (PMC6509763; doi:10.1186/s12864-019-5733-y)
Supplement: Supplementary file 8 — Table S5. The number of previously reported glyoxalase genes and glyoxalase genes with the critical conserved binding sites in grape, rice, Arabidopsis, soybean and Medicago truncatula. (DOCX 17 kb) [file 12864_2019_5733_MOESM8_ESM.docx]

**Additional file 8 Table S5.** The number of previously reported glyoxalase genes and glyoxalase genes with the critical conserved binding sites in grape, rice, *Arabidopsis*, soybean and *Medicago truncatula*

| Name of species | Classification | Genome size | Number of members | | | | | | Reference |
| --- | --- | --- | --- | --- | --- | --- | --- | --- | --- |
|  |  |  | Counts of previously reported GLYIs | Counts of GLYIs with conserved sites | Counts of previously reported GLYIIs | Counts of GLYIIs with conserved sites | Counts of previously reported GLYIIIs | Counts of GLYIIIs with conserved sites |  |
| *Vitis vinifera* | Dicot  (diploid) | 486Mb | 4 | 4 | 2 | 2 | 3 | 3 | [58] |
| *Oryza sativa* | Monocot  (diploid) | 466 Mb | 11 | 4 | 3 | 2 | 6 | 4 | [8, 10, 59] |
| *Arabidopsis thaliana* | Dicot  (diploid) | 125 Mb | 11 | 3 | 5 | 3 | 6 | 3 | [8, 10, 60] |
| *Glycine max* | Dicot  (tetraploid) | 1.1 Gb | 24 | 10 | 12 | 3 | 10 | 5 | [9, 10, 54, 61] |
| *Medicago truncatula* | Dicot  (diploid) | 375 Mb | 29 | 5 | 14 | 2 | 5 | 2 | [10, 62] |
