# Supplementary material for: Genome-wide analysis of glyoxalase-like gene families in grape (Vitis vinifera L.) and their expression profiling in response to downy mildew infection
Source: BMC Genomics. 2019 May 9;20:362. doi: 10.1186/s12864-019-5733-y (PMC6509763; doi:10.1186/s12864-019-5733-y)
Supplement: Supplementary file 9 — Figure S4. Multiple sequence alignment of C-terminal GLYI domain of VvGLYI-like1 and VvGLYI-like4. C-terminal GLYI domains of VvGLYI-like1 and VvGLYI-like4 were aligned with the C-terminal GLYI domain of OsGLYI-11 and the N-terminal GLYI domain of AtGLYI-2 using ClustalW and then edited by the Jalview program. All four conserved metal binding sites are shown in black boxes. (DOCX 247 kb) [file 12864_2019_5733_MOESM9_ESM.docx]

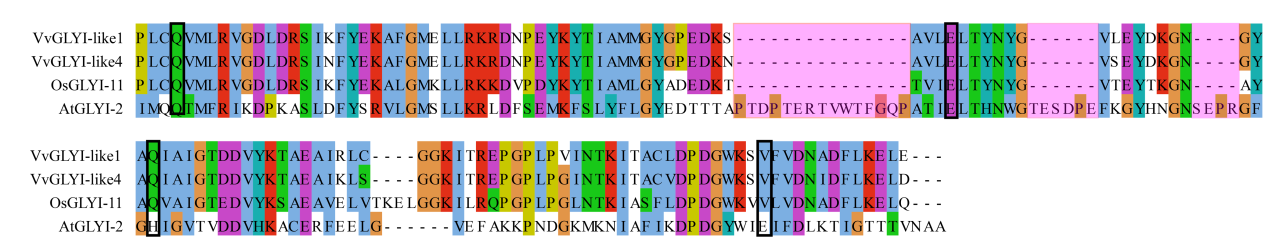


**Additional file 9: Figure S4. Multiple sequence alignment of C-terminal GLYI domain of VvGLYI-like1 and VvGLYI-like4.** C-terminal GLYI domains of VvGLYI-like1 and VvGLYI-like4 are aligned with the C-terminal GLYI domain of OsGLYI-11 and the N-terminal GLYI domain of AtGLYI-2 using ClustalW and then edited by the Jalview program. All four conserved metal binding sites are shown in black boxes.
