# Supplementary material for: Genome-wide analysis of glyoxalase-like gene families in grape (Vitis vinifera L.) and their expression profiling in response to downy mildew infection
Source: BMC Genomics. 2019 May 9;20:362. doi: 10.1186/s12864-019-5733-y (PMC6509763; doi:10.1186/s12864-019-5733-y)
Supplement: Supplementary file 10 — Table S6. Percent Identity Matrix of GLY-like families bweteen grape and Arabidopsis. (DOCX 23 kb) [file 12864_2019_5733_MOESM10_ESM.docx]

|  | VvGLYI-like1 | VvGLYI-like2 | VvGLYI-like3 | VvGLYI-like4 | VvGLYII-like1 | VvGLYII-like2 | VvGLYIII-like1 | VvGLYIII-like2 | VvGLYIII-like3 |
| --- | --- | --- | --- | --- | --- | --- | --- | --- | --- |
| VvGLYI-like1 | 100% |  |  |  |  |  |  |  |  |
| VvGLYI-like2 | 24.6% | 100% |  |  |  |  |  |  |  |
| VvGLYI-like3 | 71.6% | 22.9% | 100% |  |  |  |  |  |  |
| VvGLYI-like4 | 80.4% | 24.6% | 73.0% | 100% |  |  |  |  |  |
| VvGLYII-like1 | 11.2% | 8.3% | 7.8% | 9.1% | 100% |  |  |  |  |
| VvGLYII-like2 | 8.8% | 7.9% | 7.6% | 8.4% | 45.4% | 100% |  |  |  |
| VvGLYIII-like1 | 8.2% | 8.5% | 8.6% | 7.9% | 9.6% | 10.9% | 100% |  |  |
| VvGLYIII-like2 | 8.5% | 6.8% | 9.6% | 8.4% | 9.8% | 9.4% | 18.4% | 100% |  |
| VvGLYIII-like3 | 9.4% | 9.4% | 10.4% | 9.6% | 8.0% | 10.9% | 48.4% | 20.5% | 100% |
| AtGLYI-2 | 24.6% | 73.1% | 23.8% | 25.1% | 9.3% | 7.9% | 9.9% | 7.2% | 10.3% |
| AtGLYI-3 | 65.0% | 21.7% | 79.9% | 64.8% | 8.7% | 8.4% | 6.8% | 10.1% | 9.0% |
| AtGLYI-6 | 77.3% | 23.2% | 74.4% | 83.1% | 10.4% | 9.2% | 7.6% | 8.9% | 9.0% |
| AtGLYII-2 | 10.5% | 6.9% | 8.8% | 9.7% | 43.4% | 74.8% | 12.1% | 9.8% | 10.9% |
| AtGLYII-4 | 10.2% | 7.7% | 7.8% | 8.7% | 72.6% | 43.8% | 9.5% | 10.0% | 8.6% |
| AtGLYII-5 | 10.7% | 7.8% | 8.3% | 8.6% | 74.4% | 42.2% | 10.4% | 8.9% | 8.4% |
| AtDJ-1a | 8.8% | 8.5% | 10.4% | 9.0% | 8.5% | 8.9% | 46.6% | 19.7% | 64.4% |
| AtDJ-1b | 9.5% | 10.3% | 11.4% | 10.4% | 8.0% | 10.1% | 44.8% | 18.1% | 69.0% |
| AtDJ-1d | 10.3% | 9.5% | 11.1% | 10.2% | 8.5% | 8.3% | 16.7% | 81.3% | 19.1% |
| AtDJ-1e | 8.4% | 5.8% | 8.9% | 9.2% | 11.5% | 10.2% | 17.5% | 44.4% | 17.2% |
| AtDJ-1f | 9.0% | 5.8% | 8.8% | 9.1% | 11.8% | 9.8% | 18.2% | 43.9% | 17.2% |

**Additional file 10 Table S6.** Percent Identity Matrix of GLY-like families between grape and *Arabidopsis*

|  | AtGLYI-2 | AtGLYI-3 | AtGLYI-6 | AtGLYII-2 | AtGLYII-4 | AtGLYII-5 | AtDJ-1a | AtDJ-1b | AtDJ-1d | AtDJ-1e | AtDJ-1f |
| --- | --- | --- | --- | --- | --- | --- | --- | --- | --- | --- | --- |
| AtGLYI-2 | 100% |  |  |  |  |  |  |  |  |  |  |
| AtGLYI-3 | 22.2% | 100% |  |  |  |  |  |  |  |  |  |
| AtGLYI-6 | 24.2% | 68.4% | 100% |  |  |  |  |  |  |  |  |
| AtGLYII-2 | 6.9% | 8.8% | 9.7% | 100% |  |  |  |  |  |  |  |
| AtGLYII-4 | 8.2% | 8.6% | 9.3% | 43.0% | 100% |  |  |  |  |  |  |
| AtGLYII-5 | 8.8% | 9.2% | 9.2% | 43.4% | 84.9% | 100% |  |  |  |  |  |
| AtDJ-1a | 8.5% | 8.6% | 8.9% | 10.1% | 8.7% | 8.9% | 100% |  |  |  |  |
| AtDJ-1b | 10.7% | 9.4% | 9.8% | 9.7% | 10.1% | 9.7% | 80.8% | 100% |  |  |  |
| AtDJ-1d | 8.6% | 10.1% | 10.4% | 10.2% | 9.7% | 7.3% | 17.8% | 17.2% | 100% |  |  |
| AtDJ-1e | 8.5% | 9.3% | 9.4% | 10.2% | 13.0% | 12.9% | 13.8% | 14.9% | 43.4% | 100% |  |
| AtDJ-1f | 8.1% | 8.6% | 10.3% | 9.0% | 10.8% | 10.4% | 15.6% | 14.8% | 42.9% | 75.9% | 100% |
