# Supplementary material for: Genome-wide analysis of glyoxalase-like gene families in grape (Vitis vinifera L.) and their expression profiling in response to downy mildew infection
Source: BMC Genomics. 2019 May 9;20:362. doi: 10.1186/s12864-019-5733-y (PMC6509763; doi:10.1186/s12864-019-5733-y)
Supplement: Supplementary file 11 — Table S7. Expression analysis of VvGLY-like genes in the ESTs database. (DOCX 14 kb) [file 12864_2019_5733_MOESM11_ESM.docx]

**Additional file 11 Table S7.** Expression analysis of VvGLY-like genes in the ESTs database

| Gene name | Tissue and organ type | | | | | | | | |  | Number of ESTs in dbEST |
| --- | --- | --- | --- | --- | --- | --- | --- | --- | --- | --- | --- |
|  | root | leaf | berry | seed | bud | flower | pericarp | shoot | clusters | stem |  |
| *VvGLYI-like 1* | + | + |  |  |  | + |  | + |  |  | 61 |
| *VvGLYI-like 2* | + | + | + |  | + | + | + |  | + | + | 14 |
| *VvGLYI-like 3* | + | + | + | + | + | + | + |  | + |  | 55 |
| *VvGLYI-like 4* | + | + | + | + | + | + |  | + | + |  | 16 |
| *VvGLYII-like 1* | + | + | + | + | + | + | + |  | + |  | 43 |
| *VvGLY II-like 2* | + | + | + | + | + | + | + |  | + |  | 81 |
| *VvGLY III-like 1* |  |  |  |  | + |  |  |  |  |  | 2 |
| *VvGLY III-like 2* | + | + | + |  | + | + | + |  | + |  | 36 |
| *VvGLY III-like 3* | + | + | + | + |  | + | + |  |  | + | 35 |
