# Supplementary material for: Genome-wide analysis of glyoxalase-like gene families in grape (Vitis vinifera L.) and their expression profiling in response to downy mildew infection
Source: BMC Genomics. 2019 May 9;20:362. doi: 10.1186/s12864-019-5733-y (PMC6509763; doi:10.1186/s12864-019-5733-y)
Supplement: Supplementary file 12 — Table S8. Primers used in expression analysis of glyoxalase-like gene families in grape. (DOCX 18 kb) [file 12864_2019_5733_MOESM12_ESM.docx]

| Gene | Gene ID (Accession No.) | Forward and reverse primer sequence (5'-3') | |
| --- | --- | --- | --- |
| *VvGLYΙ-like1* | VIT_04s0008g03560 | F | TCTGGTAAAGGCAAAGGG |
|  | XM_002283932 | R | TAAGGGCTCTGGTGTAGG |
| *VvGLYΙ-like2* | VIT_06s0061g00460 | F | GCTCTATTGCCACTTCTC |
|  | XM_002276240 | R | AAACAACCGAAACCTACT |
| *VvGLY1-like3* | VIT_10s0116g01660 | F | TAAAGGTGGAAAGAGTGTC |
|  | XM_002273310 | R | GAGTGGTTCAGGCGTTGG |
| *VvGLYΙ-like4* | VIT_11s0016g03440 | F | AATCCCAAGGACAACCAC |
|  | XM_003633073 | R | CTGCACGTACTCTATCTTCATC |
| *VvGLYΙΙ-like1* | VIT_05s0102g01180 | F | TGCCAGACGAAACAAATA |
|  | XM_002271759 | R | AGCCCTTACTACGGAGAT |
| *VvGLYΙΙ-like2* | VIT_13s0067g00180 | F | CTCGTCCTCACCACTCAC |
|  | XM_002267435 | R | AGACCCACCGTACACTTC |
| *VvGLYΙΙΙ-like1* | VIT_03s0063g00300 | F | TGATACCTCCATCTCAACA |
|  | XM_002282219 | R | GAATTTCACTATCCCTCAAT |
| *VvGLYΙΙΙ-like2* | VIT_04s0079g00800 | F | AGCACTAGGAGGCACCAT |
|  | XM_010650611 | R | GGCAAACCGCATCAACAT |
| *VvGLYΙΙΙ-like3* | VIT_19s0014g01520 | F | AGGGAGTCAAATAAACCATA |
|  | XM_010645827 | R | GATCGGACAGCTTGCTAC |
| *Actin 7* | VIT_04s0044g00580 | F | CCATCCTTCGTCTTGACCTTGCTG |
|  | XM_002282480 | R | AGTGGTGAACATGTAACCCCTCTC |
| *VvMADS9* | VIT_18s0001g01760 | F | CAGCCAAACTTACAGGAGAGG |
|  | NM_001280946 | R | CACAAACCAAGATCACACACC |
| *VvPR1* | VIT_03s0088g00810 | F | GTGTAGGAGTCCATTAGC |
|  | XM_002273752 | R | GCATTGAGGTAGTCTTGT |
| *VvNPR1* | VIT_11s0016g01990  XM_002281439 | F  R | TGTGCGTAGAGGTATTGG  AACTCAGAAGTGCCATCC |
| *VvGAPDH* | VIT_17s0000g10430  XM 002263109 | F  R | TG AAGAAGAAGATGTGGAAGG C  TCATGACCAATGTGAGCAATATAGT |

**Additional file 12 Table S8.** Primers used in expression analysis of glyoxalase-like gene families in grape
