# Supplementary material for: Genome-wide analysis of glyoxalase-like gene families in grape (Vitis vinifera L.) and their expression profiling in response to downy mildew infection
Source: BMC Genomics. 2019 May 9;20:362. doi: 10.1186/s12864-019-5733-y (PMC6509763; doi:10.1186/s12864-019-5733-y)
Supplement: Supplementary file 13 — Amino acid sequences of lactoylglutathione lyase domains and full length protein sequences of GLYIs used for phylogenetic analysis and multiple sequence alignment. (DOCX 22 kb) [file 12864_2019_5733_MOESM13_ESM.docx]

**Additional file 13:** Amino acid sequences of lactoylglutathione lyase domains and full length protein sequences of GLYIs used for phylogenetic analysis and multiple sequence alignment

**Lactoylglutathione lyase domains**

>VvGLYI-1

MLHVVYRVGNLDKTMKFYTECLGMKLLRRCDIPEERYANAFLGYGPEDSHFVVELTYNYGVDKIDIGTGFGHFGIAVEDVAKTVDLVKAKGGKVTREPGPVKGGKTVIAFVEDPDGYKFEL

>VvGLYI-2

QTMYRIKDPKVSLDFYSRVLGMSLLKRLDFPEMKFSLYFMGYEDTASAPSNETERIVWTFSQKATIELTHNWGTESDPDFKGYHNGNSEPRGFGHIGITVDDTYKACERFERLGVEFVKKPDDGKMKGLAFIKDPDGYWIEI

>VvGLYI-3

FLHVVYRVGDLDRTIKFYTECFGMKLLRKRDIPEEKYTNAFLGFGPEETNFVVELTYNYGVDKYDIGTGFGHFAIATQDVYKMVEDIRAKGGIITREPGPVKGGKSVIAFAKDPDGYIFEL

>VvGLYI-4

MLHVVYRVGDLDRTIKFYTECLGMKLLRRRDIPEERYTNAFLGYGPEDSHFVIELTYNYGVDKYDIGAGFGHFGIAVEDVTKTVDLIKAKGGKVTREPGPVKGGSTVIAFIEDPDGYKFEL

>AtGLYI-2

QTMFRIKDPKASLDFYSRVLGMSLLKRLDFSEMKFSLYFLGYEDTTTAPTDPTERTVWTFGQPATIELTHNWGTESDPEFKGYHNGNSEPRGFGHIGVTVDDVHKACERFEELGVEFAKKPNDGKMKNIAFIKDPDGYWIEI

>AtGLYI-3

FLHVVYRVGDLDRTIEFYTEVFGMKLLRKRDIPEEKYSNAFLGFGPETSNFVVELTYNYGVSSYDIGTGFGHFAISTQDVSKLVENVRAKGGNVTREPGPVKGGGSVIAFVKDPDGYTFEL

>AtGLYI-6

MLHVVYRVGDMDRTIKFYTECLGMKLLRKRDIPEEKYTNAFLGYGPEDSHFVIELTYNYGVDKYDIGAGFGHFGIAVDDVAKTVELVKAKGGKVSREPGPVKGGKTVIAFIEDPDGYKFEL

>OsGLYI-2

LLHVVYRVGDIDRTIKFYTECLGMKLLRKRDIPEEKYTNAFLGYGAEDNHFVVELTYNYGVDKYDIGAGFGHFGIAVDDVAKTVELIRAKGGKVTREPGPVKGGKTVIAFVEDPDGYKFEI

>OsGLYI-7

MLHVVYRVGDLDKTIKFYTECLGMKLLRKRDIPEERYTNAFLGYGPEDSHFVVELTYNYGVESYDIGTAFGHFGIAVEDVAKTVDLIKAKGGTVTREPGPVKGGKSVIAFIEDPDGYKFEL

>OsGLYI-8

QTMFRVKDPKVSLDFYSRVMGMSLLKRLDFPEMKFSLYFLGYEDVESAPTDPVKRTVWTFGQRATLELTHNWGTENDPEFKGYHNGNSDPRGFGHIGVTVHDVYKACERFERLGVEFVKKPDDGKMKGIAFIKDPDGYWIEI

>OsGLYI-11

LLHAVYRVGDLDRTIKCYTECFGMKLLRKRDVPEEKYTNAFLGFGPEDTNFALELTYNYGVDKYDIGAGFGHFAIATEDVYKLAEKIKSSCCCKITREPGPVKGGSTVIAFAQDPDGYMFEL

>GmGLYI-1

MLHVVYRVGDLDKSIKFYRECLGMKLLRKRDMQEQKYTNAFLGYGPEDAHFVVELTYSNSYGIEKYDIGDGFGHFGIAIDDISRIVELVRAKGGKITREPSPVKGGNTTIAYIEDPDGYQFEL

>GmGLYI-3

FLHVVYRVGDLEKTIKFYTECLGMKLLRQRDIPEDRYSNAFLGYGPEDSNFTVELTYNYGVDNYDIGSGFGHFGVAVEDIYKRVDLVKAKGGKVTREPGPVKDGSAVIAFIEDPDGYKFEL

>GmGLYI-4

MLHVVYRVGDLDRTIKFYTECLGMKLLRKRDIPEEKYTNAFLGYGPEDSHFVIELTYNYGVDKYDIGTGFGHFGIAVDDVAKAVELIRAKGGKITREPGPVKGGRSVIAFIEDPDGYKFEL

>GmGLYI-8

MLHVVYRVGDLDRTIKFYTECLGMKLLRKRDIPEEKYTNAFLGYGPEDSHFVIELTYNYGVDKYDIGTGFGHFGIAVDDVAKAVELIRAKGGKITREPGPVKGGRSVIAFIEDPDGYKFEL

>GmGLYI-10

FLHVVYRVGDLDRTIKFYTECFGMKLLRKRDIPEEKYANAFLGFGPEQSHFVVELTYNYGVTSYDIGTGFGHFAIATPDVYKLVEDIRAKGGNITREPGPVKGGKSVIAFVKDPDGYAFEL

>GmGLYI-11

MLHVVYRVGDLDKSIKFYRECLGMKLLRKRDMQEQRYTNAFLGYGPEDAHFVAELTYNYGIDKYDIGDGFGHFGLAVDDISRIVELVRAKGGKITREPSPVKGGNSTIAYIEDPDGYQFEL

>GmGLYI-14

TMFRIKDPKVSLDFYSRVLGTYLLKRLDFLEMKFSLYFMGYEDTTKAPSNPVERTVWTFSQKATMELTDNWGTENDPEFKGYHNGNSEPLGYGHIGIAVDDTYKACERFQNLGVEFVTKPDDGFFSQHYCFKHYFQVFFILELFSAGEIKGLAFIKDPDGYWIEL

>GmGLYI-15

QTMFRIKDPKVSLDFYSRVLGMSLLKRLDFPEMKFSLYFMGYENTAEAPSNPIDKVVWTFSQKATIELTHNWGTESDPEFKGYHNGNSEPRGFGHIGVTVDDTYKACERFQNLGVEFVKKPEDGKMKGIAFIKDPDGYWIEI

>GmGLYI-16

QTMFRIKDPKVSLDFYSRVLGMSLLKRLDFPEMKFSLYFMGYEDTTEAPSNPIDKVVWTFSQKATIELTHNWGTESDPEFKGYHNGNSEPRGFGHIGITVDDTYKACERFQNLGVEFVKKPDDGKMKGIAFIKDPDGYWIEI

>GmGLYI-21

FLHVVYRVGDLDRTIKFYTECFGMKLLRKRDIPEEKYANAFLGFGPEQSHFVVELTYNYGVTSYDIGTGFGHFAIATPDVYKLVEDIRAKGGNVTREPGPVKGGKSVIAFVKDPDGYAFEL

>MtGLYI-4

LHAVYRVGDLDRTIKFYTEAFGMKLLRKRDVPEEKYANAFLGFGPETSNFVVELTYNYGVTSYDIGTGFGHFAIATPDVYKFVENARAKGGKVTREPGPVSGGTSVIAFVADPDGYLFEI

>MtGLYI-7

FLHVVYKVGDLDKTIKFYTECLGMKLLRKRDIPEDKYSNAFLGYGPEDSSFTVELTYNYGVDNYDIGTGFGHFGIIAEDVSKTVDIVKAKGGKVTREPGSVKGGSIVTASVEDPSGYRFEL

>MtGLYI-10

QTMFRIKDPKVSLDFYSRVLGMSLLKRLDFPEMKFSLYFMGYEDTSEAPSNSVDRTVWTFAQKATIELTHNWGTESDPEFKGYHNGNSDPRGFGHIGITVDDTYKACERFQNLGVEFVKKPEDGKMKGIAFIKDPDGYWIEI

>MtGLYI-22

MLHVVYRVGDFDKSIKFYTECLGMKVLRKRDMTEEKYTNAFLGYGPEDAHFAIELTYNYGIETYDIGTGFGHYGIAMDDISRVVDIVRAKGGIITREPGPVKGGDSTVAVIEDPDGYKFEL

>MtGLYI-24

LHVVYRVGDLDRTIKFYTECLGMKLLRKRDIPEERYTNAFLGYGPEDSHFVIELTYNYGVDKYDIGTAFGHFGIAVDDITKTVELIRAKGGKITREPGPVKGGKTVIAFVEDPDGYKFEL

>H.sapiens(GlyI)

LQQTMLRVKDPKKSLDFYTRVLGMTLIQKCDFPIMKFSLYFLAYEDKNDIPKEKDEKIAWALSRKATLELTHNWGTEDDETQSYHNGNSDPRGFGHIGIAVPDVYSACKRFEELGVKFVKKPDDGKMKGLAFIQDPDGYWIEI

**Full length protein sequences**

>VvGLYI-1 (Accession No: XP_002283968)

MRTLPMATTPFQHLSSSPPFLSSLRFPTSSFASSLTGFTSSRRLALFHLGTAIPQSELLGGKTLKLFRMEGNMLEAGTAGNMAQAAVSEGNVLEWAKTDKRRMLHVVYRVGNLDKTMKFYTECLGMKLLRRCDIPEERYANAFLGYGPEDSHFVVELTYNYGVDKIDIGTGFGHFGIAVEDVAKTVDLVKAKGGKVTREPGPVKGGKTVIAFVEDPDGYKFELLEREPTPEPLCQVMLRVGDLDRSIKFYEKAFGMELLRKRDNPEYKYTIAMMGYGPEDKSAVLELTYNYGVLEYDKGNGYAQIAIGTDDVYKTAEAIRLCGGKITREPGPLPVINTKITACLDPDGWKSVFVDNADFLKELE

>VvGLYI-2 (Accession No: XP_002276276)

MASCSIATSLSRLSLLRLIPKPSSSYSSSIPLFPTTTRKDPSRFRLFSASMASEPKESPSNNPGLHSSPDEATKGYFMQQTMYRIKDPKVSLDFYSRVLGMSLLKRLDFPEMKFSLYFMGYEDTASAPSNETERIVWTFSQKATIELTHNWGTESDPDFKGYHNGNSEPRGFGHIGITVDDTYKACERFERLGVEFVKKPDDGKMKGLAFIKDPDGYWIEIFDLRRIGTVSTTAA

>VvGLYI-3 (Accession No: XP_002273346)

MAEAAPVVPSDELLEWPKKDKRRFLHVVYRVGDLDRTIKFYTECFGMKLLRKRDIPEEKYTNAFLGFGPEETNFVVELTYNYGVDKYDIGTGFGHFAIATQDVYKMVEDIRAKGGIITREPGPVKGGKSVIAFAKDPDGYIFELIQRGPTPEPLCQVMLRVGDLERSIKFYEKALGMKMVKKTDRPEYKYSIAMMGYAEEHETTVLELTYNYGVTEYTKGNAYAQVAISTDDVYKSAEVVNLVTKELGGKITRQPGPIPGLNTKITSFLDPDGWKTVLVDNEDFLKELHKEE

>VvGLYI-4 (Accession No: XP_003633121)

MVRIIPMATSFRPSLSSFGFSTSSRLGFPLSTFNISRTVTSLHVGSAIPQSQIFGLNASKLLRGGEGNAMGFNATGNIAHASTSAAQENVLEWVKKDKRRMLHVVYRVGDLDRTIKFYTECLGMKLLRRRDIPEERYTNAFLGYGPEDSHFVIELTYNYGVDKYDIGAGFGHFGIAVEDVTKTVDLIKAKGGKVTREPGPVKGGSTVIAFIEDPDGYKFELLERGPTPEPLCQVMLRVGDLDRSINFYEKAFGMELLRKRDNPEYKYTIAMMGYGPEDKNAVLELTYNYGVSEYDKGNGYAQIAIGTDDVYKTAEAIKLSGGKITREPGPLPGINTKITACVDPDGWKSVFVDNIDFLKELD

>AtGLYI-2 (Accession No: AT1G08110)

MSSYSIASAISRISPLIRFVKPYSTGFSFITCACNSTRRPKRFDQLCVFSMASEARESPANNPGLSTNRDEATKGYIMQQTMFRIKDPKASLDFYSRVLGMSLLKRLDFSEMKFSLYFLGYEDTTTAPTDPTERTVWTFGQPATIELTHNWGTESDPEFKGYHNGNSEPRGFGHIGVTVDDVHKACERFEELGVEFAKKPNDGKMKNIAFIKDPDGYWIEIFDLKTIGTTTVNAA

>AtGLYI-3 (Accession No: AT1G11840)

MNEIASASMLRLCQCFISICNVHFVSMRAAESSFLLSRNMAEASDLLEWPKKDNRRFLHVVYRVGDLDRTIEFYTEVFGMKLLRKRDIPEEKYSNAFLGFGPETSNFVVELTYNYGVSSYDIGTGFGHFAISTQDVSKLVENVRAKGGNVTREPGPVKGGGSVIAFVKDPDGYTFELIQRGPTPEPFCQVMLRVGDLDRAIKFYEKALGMRLLRKIERPEYKYTIGMMGYAEEYESIVLELTYNYDVTEYTKGNAYAQIAIGTDDVYKSGEVIKIVNQELGGKITREAGPLPGLGTKIVSFLDPDGWKTVLVDNKDFLKELE

>AtGLYI-6 (Accession No: AT1G67280)

MVRIIPMAASSIRPSLACFSDSPRFPISLLSRNLSRTLHVPQSQLFGLTSHKLLRRSVNCLGVAESGKAAQATTQDDLLTWVKNDKRRMLHVVYRVGDMDRTIKFYTECLGMKLLRKRDIPEEKYTNAFLGYGPEDSHFVIELTYNYGVDKYDIGAGFGHFGIAVDDVAKTVELVKAKGGKVSREPGPVKGGKTVIAFIEDPDGYKFELLERGPTPEPLCQVMLRVGDLDRAIKFYEKAFGMELLRTRDNPEYKYTIAMMGYGPEDKFPVLELTYNYGVTEYDKGNAYAQIAIGTDDVYKTAEAIKLFGGKITREPGPLPGISTKITACLDPDGWKSVFVDNIDFLKELE

>OsGLYI-2 (Accession No: LOC_Os02g17920)

MRALPMAAGRAAAVAACASPAVPRRSLLLSTAAAGEPPCRPPADSSSPSKFSRFDRSAVRLLGWTAALQPEPVRLTRGASAAPKLRASPPDAAQAAAAFGSKEEAFAWAKSDNRRLLHVVYRVGDIDRTIKFYTECLGMKLLRKRDIPEEKYTNAFLGYGAEDNHFVVELTYNYGVDKYDIGAGFGHFGIAVDDVAKTVELIRAKGGKVTREPGPVKGGKTVIAFVEDPDGYKFEILERPGTPEPLCQVMLRVGNLDRAISFYEKACGMELLRKRDNPEYKYTVAMMGYGPEDKNAVLELTYNYGVTEYDKGNAYAQIAIGTDDVYKTAEVVKLFGGQVVREPGPLPGINTKITSILDPDGWKSVFVDNIDFAKELE

>OsGLYI-7 (Accession No: LOC_Os05g14194)

MARLLLPLPIAAAAASRLRLPVLSSSVARREALLFGGRVAAARAPVRLARRGVSAGAEAGGSSSAAAAAQVIGQDEAVEWVKKDRRRMLHVVYRVGDLDKTIKFYTECLGMKLLRKRDIPEERYTNAFLGYGPEDSHFVVELTYNYGVESYDIGTAFGHFGIAVEDVAKTVDLIKAKGGTVTREPGPVKGGKSVIAFIEDPDGYKFELIERGPTPEPLCQVMLRVGDLDHAINFYEKAFGMELLRKRDNPQYKYTIAMMGYGPEDKNAVLELTYNYGVKEYDKGNAYAQIAISTDDVYKTAEVIRQNGGQITREPGPLPGINTKITACTDPDGWKTVFVDNVDFLKELEE

>OsGLYI-8 (Accession No: LOC_Os05g22970)

MAAAAIAAASLLPSSAFALRRLSSAANVSRFAQLKRFDRARRFAPAAAMSTSSGPKEAPANNPGLQAPSEKDPATKGYFMQQTMFRVKDPKVSLDFYSRVMGMSLLKRLDFPEMKFSLYFLGYEDVESAPTDPVKRTVWTFGQRATLELTHNWGTENDPEFKGYHNGNSDPRGFGHIGVTVHDVYKACERFERLGVEFVKKPDDGKMKGIAFIKDPDGYWIEIFDLNRIGAVTAEAS

>OsGLYI-11 (Accession No: LOC_Os08g09250)

MASGSEAEKSPEVVLEWPKKDKKRLLHAVYRVGDLDRTIKCYTECFGMKLLRKRDVPEEKYTNAFLGFGPEDTNFALELTYNYGVDKYDIGAGFGHFAIATEDVYKLAEKIKSSCCCKITREPGPVKGGSTVIAFAQDPDGYMFELIQRGPTPEPLCQVMLRVGDLDRSIKFYEKALGMKLLRKKDVPDYKYTIAMLGYADEDKTTVIELTYNYGVTEYTKGNAYAQVAIGTEDVYKSAEAVELVTKELGGKILRQPGPLPGLNTKIASFLDPDGWKVVLVDNADFLKELQ

>GmGLYI-1 (Accession No: Glyma.01g146300)

MSSSIRPSLSSFMLPSLASCNPSQKLSLFRLGSGIRQFHKFGLKASRFLRHDDKCMRVMAFGNMSTAATQENVLDWVKHDKRRMLHVVYRVGDLDKSIKFYRECLGMKLLRKRDMQEQKYTNAFLGYGPEDAHFVVELTYSNSYGIEKYDIGDGFGHFGIAIDDISRIVELVRAKGGKITREPSPVKGGNTTIAYIEDPDGYQFELLERVPSPEPLCKVMLRVGDLDRSIKFYEKAFGMELLRTQDDPESKSTIGILGYGPEEKNTVLELTYNYGVTNYDKGDAYAQITIDTDDVYKTAEAIKLAGGKITREPGPIPVMKTKITSCVDPDGWKTVFVDNVDFRRELE

>GmGLYI-3 (Accession No: Glyma.04g083100)

MSSSLMLPAASMLRPCTTSSSSCTSSRRLALFHLVSTGSIALPQAQLFGAKGPELLRVVEASAAEKLAQPEKDLFDWVKNDNRRFLHVVYRVGDLEKTIKFYTECLGMKLLRQRDIPEDRYSNAFLGYGPEDSNFTVELTYNYGVDNYDIGSGFGHFGVAVEDIYKRVDLVKAKGGKVTREPGPVKDGSAVIAFIEDPDGYKFELLERRPTSEPLCQVMLRVGDLDRAIAFYEKAVGMKLLRKRDNPEQKYTVAFMGYGPEDKNTVLELTYNYGVTNYDKGNGYAQIAIGTNDVYKTAEAIKLCGGKIIREPGPLPGINTKIVACLDPDGWKLAFVDNVDFLKELE

>GmGLYI-4 (Accession No: Glyma.05g228500)

MVLVRVVPMASSSSIRPTLSSLRFLTPSSLSLSNPSSRISFSHLPSPSVSQSNSFGLKASRELRQHGNSTRIMASGDVSQSISAASPENVLEWVKQDKRRMLHVVYRVGDLDRTIKFYTECLGMKLLRKRDIPEEKYTNAFLGYGPEDSHFVIELTYNYGVDKYDIGTGFGHFGIAVDDVAKAVELIRAKGGKITREPGPVKGGRSVIAFIEDPDGYKFELIERGPTPEPLCQVMLRVGDLNRSIEFYEKAFGMELLRTRDNPEYKYTIAMLGYGPEDKSTVLELTYNYGVTEYDKGNAYAQIAVGTDDVYKTAEAIKLAGGKITREPGALPGINTKITACLDPDGWKSYITRSGRSVFCLLWHDT

>GmGLYI-8 (Accession No: Glyma.08g035400)

MVLVRLVPMASSSIRPALSTPSSFSLFSPSRRISFSHLPSPSVSQSNSFGLKASRVLRQYGNSTRIMASGDLSHSVAAASPENVLEWVKQDKRRMLHVVYRVGDLDRTIKFYTECLGMKLLRKRDIPEEKYTNAFLGYGPEDSHFVIELTYNYGVDKYDIGTGFGHFGIAVDDVAKAVELIRAKGGKITREPGPVKGGRSVIAFIEDPDGYKFELIERGPTPEPLCQVMLRVGDLNRSIEFYEKAFGMELLRTRDNPEYKYTIAMLGYGPEDKSTVLELTYNYGVTEYDKGNAYAQIAIGTDDVYKTAEAIKLAGGKITREPGPLPGINTKITACLDPDGWKSVFVDNVDFLKELE

>GmGLYI-10 (Accession No: Glyma.09g004300)

MLLYKATRPQCRRLFWFVVLCLAIFTIFTEHLHSTLNMAEATQSNAELLEWPKKDKRRFLHVVYRVGDLDRTIKFYTECFGMKLLRKRDIPEEKYANAFLGFGPEQSHFVVELTYNYGVTSYDIGTGFGHFAIATPDVYKLVEDIRAKGGNITREPGPVKGGKSVIAFVKDPDGYAFELIQRSSTPEPLCQVMLRVGDLERSIKFYEKTLGLRVVKKTDRPEYKYTIAMLGYAEEHETTVLELTYNYGVTEYTKGNAYAQVAIGTDDVYKSAEVVNIVTQELGGKITRQPGPVPGLNTKITSFLDPDGWKTVLVDNQDFLKELE

>GmGLYI-11 (Accession No: Glyma.09g193800)

MASSIRPSLSSFMLPSLRSCNPSEKLSLFHLGSGIRLYHKFGLKSSRLLRHDDNKCMRVMASGNMSTAATQENVLDWVKHDKRRMLHVVYRVGDLDKSIKFYRECLGMKLLRKRDMQEQRYTNAFLGYGPEDAHFVAELTYNYGIDKYDIGDGFGHFGLAVDDISRIVELVRAKGGKITREPSPVKGGNSTIAYIEDPDGYQFELSERVSSPEPLSKVMLRVGDLDRSIKFYEKAFGMELLRTQDDPESKSTIAILGYGPEEKNTVLELTYNYGVTDYDKGDAYAQITIGTDDVYKTAEAIKLAGGKITREPGPVPGIKTKITLCVDPDGWKTVFVDNVDFRRELE

>GmGLYI-14 (Accession No: Glyma.11g194200)

MKDSSSAMVHSMRRSFFNFCLTEKAQLDLYGHSNRINCDCSEPKESPSNNPGLHTTPDQATKAYFTQQTMFRIKDPKVSLDFYSRVLGTYLLKRLDFLEMKFSLYFMGYEDTTKAPSNPVERTVWTFSQKATMELTDNWGTENDPEFKGYHNGNSEPLGYGHIGIAVDDTYKACERFQNLGVEFVTKPDDGFFSQHYCFKHYFQVFFILELFSAGEIKGLAFIKDPDGYWIELFDLKILGGEQAAAHA

>GmGLYI-15 (Accession No: Glyma.11g194300)

MTVTASLHRLSRLRFIAKPQPFLSPHSIPSHFSLTPKTKKANRFRFLSMAAEPKESPSNNPGLHTTPDEATKGYIMQQTMFRIKDPKVSLDFYSRVLGMSLLKRLDFPEMKFSLYFMGYENTAEAPSNPIDKVVWTFSQKATIELTHNWGTESDPEFKGYHNGNSEPRGFGHIGVTVDDTYKACERFQNLGVEFVKKPEDGKMKGIAFIKDPDGYWIEIFDRKTIGNVTQTAA

>GmGLYI-16 (Accession No: Glyma.12g079700)

MAATASLHRLSRLRFIAKPQPFLSPHSTPSHFSLTPKTKKPNRFRFRFRSMAAEPKESPSNNPGLHTTPDEATKGYIMQQTMFRIKDPKVSLDFYSRVLGMSLLKRLDFPEMKFSLYFMGYEDTTEAPSNPIDKVVWTFSQKATIELTHNWGTESDPEFKGYHNGNSEPRGFGHIGITVDDTYKACERFQNLGVEFVKKPDDGKMKGIAFIKDPDGYWIEIFDRKTIGNVTQAPA

>GmGLYI-21 (Accession No: Glyma.15g108400)

MAEATQSNAELLEWPKKDKRRFLHVVYRVGDLDRTIKFYTECFGMKLLRKRDIPEEKYANAFLGFGPEQSHFVVELTYNYGVTSYDIGTGFGHFAIATPDVYKLVEDIRAKGGNVTREPGPVKGGKSVIAFVKDPDGYAFELIQRPSTPEPLCQVMLRVGDLERSIKFYEKALGLRVVKKTDRPEYKYTIAMLGYAEEHETTVLELTYNYGVTEYTKGNAYAQVAIGTDDVYKSAEVVNIVTQELGGKITRQPGPIPGLNTKITAFLDPDGWKTVLVDNQDFLKELE

>MtGLYI-4 (Accession No: Medtr2g023500)

MAEAAQPNAELLEWAKKDKRRFLHAVYRVGDLDRTIKFYTEAFGMKLLRKRDVPEEKYANAFLGFGPETSNFVVELTYNYGVTSYDIGTGFGHFAIATPDVYKFVENARAKGGKVTREPGPVSGGTSVIAFVADPDGYLFEILQRASTPEPLCQVMLRVGDLERSIKFYEKALGLKLARTIDRPQYKYTLAMLGYAEEHETIVLELTYNYGVTEYTKGNAYAQVAVGTDDVYKSAELVNLATQEFGGKITRQPGPIPGLNTKITSFLDPDGWKTVLVDNQDFLKELE

>MtGLYI-7 (Accession No: Medtr3g110185)

MHAIAMDVYGKRSLLTLMDKEKTEQQESPNIHLHVQIHSHREANEQDIQFSPPRPSTTFPQSPWTLSSLPPPSPSLLYHCIASLHRHEGNIYSIAVSKGFIFTGSNSSRIRVWKQPDCMDKGYLKSNSGEIRTILAYNNMVFSSHKDHKIRIWNFNVSENFKSKKVATLPKRSKNSFLNFSRTKNNNSHNHKHKDLVSCMAYYHSEGLLYTGSHDRTVKAWRISDRNCVDSFLAHEDHVNAILVNQDDGCVFTCSSDGSVKIWRRVYTENSHTLTMTLKFQHSPVNTLALSSSFNHCFLYSGSSDGMINFWEKERLCYRFNHGGFLQGHRFAVLCVETVGNMVFSGSEDTTIRVWRREEDSCYHECLMVLDGHRGPVRCLAACLEMEKVVVGFLVYSASLDQTFKVWRIKVFSEDENVCLDGDNNKCDGRVKKIREYDMSPVLSPSWVEKKLQGGNKETDKGRSVSFICITMISSLMLPSATTLRPCCSCSITPSSSSSSSRRIALFHLLTTGGIALPQSQLLGGKGSDLFQIAEANAAVNLAQPDQNLFNWVQNDNRRFLHVVYKVGDLDKTIKFYTECLGMKLLRKRDIPEDKYSNAFLGYGPEDSSFTVELTYNYGVDNYDIGTGFGHFGIIAEDVSKTVDIVKAKGGKVTREPGSVKGGSIVTASVEDPSGYRFELLERRPTREPLCKVMLRVGDLDRVIAFYEKAVGMKLLHKIDNPEEKYTVAKLGYGPEANGPVLQLTYNYGVTNYDKGNGYAQIAIGTDDVYKTAEAIKSCGGKIIREPGPLPGINTKIVVCLDPDGWKLVFVDNVDFLKELE

>MtGLYI-10 (Accession No: Medtr4g057685)

MMSIATSNFLSRFRFIAKHQSLPIRSPVSIPFHFSLKKQPIRRFRFFSMAASESKESPANNPGLHATVDEATKGYFMQQTMFRIKDPKVSLDFYSRVLGMSLLKRLDFPEMKFSLYFMGYEDTSEAPSNSVDRTVWTFAQKATIELTHNWGTESDPEFKGYHNGNSDPRGFGHIGITVDDTYKACERFQNLGVEFVKKPEDGKMKGIAFIKDPDGYWIEIFDRKTIGNVTGSAA

>MtGLYI-22 (Accession No: Medtr6g087120)

MASSSIRPSLSSLNKLPSFSSRNLSQRFSLFHLRNGVRLLPQNFGLKASRLLRHDSGSMRVMASRSMSQSVTQENALDWVKWDKRRMLHVVYRVGDFDKSIKFYTECLGMKVLRKRDMTEEKYTNAFLGYGPEDAHFAIELTYNYGIETYDIGTGFGHYGIAMDDISRVVDIVRAKGGIITREPGPVKGGDSTVAVIEDPDGYKFELLERAPSPEPLCKVMLRVGDLDRSIKFYEKVVGMELLRKQDDPESKCTVAIMGYGPEEKTTVLELTYNYGITKYDKGDAYAQIAIGTDDVYKTAEAIKLAGGKITREAGPVPGYRTKITSCVDPDGWKTVFVDNHDFHKELE

>MtGLYI-24 (Accession No: Medtr8g102980)

MVRVIPIASSSILPTLSLFNRTPRISFSHFSTAVPQSHNFGLKACRLFKQNGNSLKVMSSGNVSSSVTAASPENVLEWVKQDKRRMLHVVYRVGDLDRTIKFYTECLGMKLLRKRDIPEERYTNAFLGYGPEDSHFVIELTYNYGVDKYDIGTAFGHFGIAVDDITKTVELIRAKGGKITREPGPVKGGKTVIAFVEDPDGYKFELLERGPTPEPLCQVMLRVGDLNRSIEFYEKAFGMELLRTRDNPDNKYTIAMLGYGPEDKSTVLELTYNYGVTEYDKGNAYAQIAIGTDDVYKTAEAIKLSTGKLTREPGPLPGINTKITACLDPDGWKTVFVDNIDFLKELE

>H.sapiens(GlyI) (Accession No: AB209801)

MAEPQPPSGGLTDEAALSYCSDADPSTKDFLLQQTMLRVKDPKKSLDFYTRVLGMTLIQKCDFPIMKFSLYFLAYEDKNDIPKEKDEKIAWALSRKATLELTHNWGTEDDETQSYHNGNSDPRGFGHIGIAVPDVYSACKRFEELGVKFVKKPDDGKMKGLAFIQDPDGYWIEILNPNKMATLM
