# Supplementary material for: Genome-wide analysis of glyoxalase-like gene families in grape (Vitis vinifera L.) and their expression profiling in response to downy mildew infection
Source: BMC Genomics. 2019 May 9;20:362. doi: 10.1186/s12864-019-5733-y (PMC6509763; doi:10.1186/s12864-019-5733-y)
Supplement: Supplementary file 14 — Amino acid sequences of full length putative GLYIIs used for phylogenetic analysis and multiple sequence alignment. (DOCX 17 kb) [file 12864_2019_5733_MOESM14_ESM.docx]

**Additional file 14:** Amino acid sequences of full length putative GLYIIs used for phylogenetic analysis and multiple sequence alignment

>VvGLYII-1 (Accession No: XP_002271795)

MQVISKAPSAMASFSCSSRGGNGLGVWPGMRQLCLRKSLLYGLMRLLSTPFKTLRTAGRTLKVAQFCSVSNTSSTLQIELVPCLKDNYAYLLHDVDTGTVGVVDPSEAVPVIDALSRKNRNLTYILNTHHHHDHTGGNAELKARYGAKVIGSGIDKDRIPGIDIVLKDGDKWMFAGHEVVVIETPGHTRGHISFYFPGSGAIFTGDTLFSLSCGKLFEGTPEQMHSSLSKIMSLPDETNIYCGHEYTLSNSKFALSIEPKNEVLQSYATHVAHLRSKGLPTIPTTLKMEKMCNPFLRTSSPEIRKSLKITATADDSEALGIIREAKDNF

>VvGLYII-2 (Accession No: XP_002267471)

MKIVPVPCLEDNYSYLIIDESSKEAAVVDPVEPQKVLQAAYEYGVHLKLVLTTHHHWDHAGGNEKIKQLVPGIEVYGGSVDNVKGCTHPLQNGDKLSLGSDLAVLALHTPCHTRGHISYYVTGKEEDVPAVFTGDTLFVAGCGKFFEGTAEQMYQSLCVTLASLPKPTRVYCGHEYTVKNLQFALTVEPDNVRVGQKLSWAQHQRQAGLPTIPSTIDEEMETNPFMRVDLPELQERVGCQSAIDALQEIRRQKDNWRG

>AtGLYII-2 (Accession No: AT1G53580)

MKIFHVPCLQDNYSYLIIDESTGDAAVVDPVDPEKVIASAEKHQAKIKFVLTTHHHWDHAGGNEKIKQLVPDIKVYGGSLDKVKGCTDAVDNGDKLTLGQDINILALHTPCHTKGHISYYVNGKEGENPAVFTGDTLFVAGCGKFFEGTAEQMYQSLCVTLAALPKPTQVYCGHEYTVKNLEFALTVEPNNGKIQQKLAWARQQRQADLPTIPSTLEEELETNPFMRVDKPEIQEKLGCKSPIDTMREVRNKKDQWRG

>AtGLYII-4 (Accession No: AT2G43430)

MQAISKVSSAASFFRCSRKLVSQPCVRPCVRQLHVRKGLVSGVMKLFSSPLRTLRDAGKSVRISRFCSVSNVSSSLQIELVPCLTDNYAYILHDEDTGTVGVVDPSEAVPVMDALQKNSRNLTYILNTHHHYDHTGGNLELKDRYGAKVIGSAADRDRIPGIDVALKDADKWMFAGHEVHIMETPGHTRGHISFYFPGARAIFTGDTLFSLSCGKLFEGTPEQMLASLQRIIALPDDTSVYCGHEYTLSNSKFALSIEPTNEVLQSYAAYVAELRDKKLPTIPTTMKMEKACNPFLRTENTDIRRALGIPETADEAEALGIIRRAKDNFKA

>AtGLYII-5 (Accession No: AT3G10850)

MQTISKASSATSFFRCSRKLSSQPCVRQLNIRKSLVCRVMKLVSSPLRTLRGAGKSIRVSKFCSVSNVSSLQIELVPCLKDNYAYILHDEDTGTVGVVDPSEAEPIIDSLKRSGRNLTYILNTHHHYDHTGGNLELKDRYGAKVIGSAMDKDRIPGIDMALKDGDKWMFAGHEVHVMDTPGHTKGHISLYFPGSRAIFTGDTMFSLSCGKLFEGTPKQMLASLQKITSLPDDTSIYCGHEYTLSNSKFALSLEPNNEVLQSYAAHVAELRSKKLPTIPTTVKMEKACNPFLRSSNTDIRRALRIPEAADEAEALGIIRKAKDDF

>OsGLYII-2 (Accession No: LOC_Os03g21460)

MKIIPVACLEDNYAYLIVDESTKSAAAVDPVEPEKVLAAAAEVGVRIDCVLTTHHHWDHAGGNEKMAQSVPGIKVYGGSLDNVKGCTDQVENGTKLSLGKDIEILCLHTPCHTKGHISYYVTSKEEEDPAVFTGDTLFIAGCGRFFEGTAEQMYQSLCVTLGSLPKPTQVYCGHEYTVKNLKFILTVEPDNEKVKQKLEWAQKQREANQPTIPSTIGEEFETNTFMRVDLPEIQAKFGAKSPVEALREVRKTKDNWKS

>OsGLYII-3 (Accession No: LOC_Os09g34100)

MRMLSKACSLVASSLPRCSSSAAPTIRGQPSLLPSVRKEWLGKPLLYGIGTLLVMPLRTLHGVGRMFGAGRFLCNMTSVSSSLQIELVPCLQDNYAYILHDVDTGTVGVVDPSEATPIINALEKRNQNLTYILNTHHHYDHTGGNLELKAKYGAKVIGSAKDRDRIPGIDITLSEGDTWMFAGHQVLVMETPGHTSGHVCYHFPGSGAIFTGDTLFSLSCGKLFEGTPQQMYSSLQKIIALPDETRVYCGHEYTLSNSKFALSIEPGNKDLQEYAANAADLRKRNTPTVPTTIGREKQCNPFLRTSSPEIKNTLSIPDHFDDARVLEVVRRAKDNF

>GmGLYII-7 (Accession No: Glyma.13g345400)

MLHMFSKASSAMATFPCSRVKSGLCVWPDVRQLCFRKGMLYGFMRLFSTPLKTLRGASRSLRVTQFCSVANMSSSLQIELVPCLKDNYAYLLHDVDTGTVGVVDPSEAVPIIDALSRKNRNLTYILNTHHHHDHTGGNVELKARYGAKVIGSGTDKERIPGIDIHLNDGDKWMFAGHEVRVMDTPGHTRGHISFYFPGSGAIFTGDTLFSLSCGKLFEGTPQQMLSSLKKIMSLSDDTNIYCGHEYTLNNIKFALSIEPENEELQSYAAQVAYLRSKGLPTIPTTLKVEKACNPFLRTSSAAIRQSLKIAATANDAEALGVIRQAKDNF

>GmGLYII-8 (Accession No: Glyma.14g187700)

MRIHHIACLQDNYSYLIVDESTKEAAAVDPVEPEKVLEVASSHGLTLKFVLTTHHHWDHAGGNDKIKQLVPGIKVYGGSIENVKGCTDKVENGDKVSLGAEITILALHTPCHTQGHISYYVTGKEDEQPAVFTGDTLFIASCGKFFEGTAEQMYQSLNVTLASLPKSTRVYCGHEYSVNNLQFALTLEPDNLRIQQKLTWARNQRQAGQATIPSTIEDELETNPFMRVDLPEIQERVGCKSPVEALGEIRKQKDNWRG

>GmGLYII-9 (Accession No: Glyma.15g028900)

MLSKPSSAMPTFPSSMVRSGLCVWPNVRQLCFRKGILYGFMRLFSTPLKTLRGASRSLRVAQFCSVANMSSSLQIELVPCLKDNYAYLLHDVDTGTVGVVDPSEAVPVIDALSRKNRNLTYILNTHHHHDHTGGNVELKARYGAKVIGSGTDKKRIPGIDIHLNDGDKWMFAGHEVRVMDTPGHTQGHISFYFPGSGAIFTGDTLFSLSCGKLFEGTPQQMLSSLKKIMSLPDNTNIYCGHEYTLNNTKFALSIEPENEELQSYAAQVAYLRSKGLPTIPTTLKMEKACNPFLRTSSAAIRQSLNIAATANDAEALGGIRQAKDNF

>MtGLYII-7 (Accession No: Medtr2g099090)

MLSKASTTAMSAFSSCSRVRTGFSVWPNVRQLCFRKGILYGFMRLFSTPYKTLRGGASRSLRVARFCSVANMSSSLQIELVPCLSDNYAYILHDIDTGTVGVVDPSEATPVIDALSKKNRNLNYILNTHHHHDHTGGNVELKARYGAKVIGSATDKERIPGIDIHLNDGDKWMFAGHEVQVMDTPGHTRGHISFYFAGSGAIFTGDTLFSLSCGKLFEGTPQEMQSSLGKIMSLPDDTSIYCGHEYTLNNTDFALKLEPGNKELRSYAGHVASLRSKGLPTIPTTLKMEKACNPFLRTSNAQIRQLLNIPATADDAEALGIIRQAKDNF

>MtGLYII-12 (Accession No: Medtr5g068440)

MKIYHVPCLEDNYSYLIVDESTKEAAAVDPVEPEKVLEASNSLGLTIKFVLTTHHHWDHAGGNEKIKELVPGIKVYGGSIDNVKGCTNALENGDKVHLGADINILALHTPCHTKGHISYYVTGKEDEDPAVFTGDTLFIAGCGKFFEGTAEQMYQSLSVTLGSLPKPTRVYCGHEYSVKNLQFALTVEPDNLRILEKLTWAQNQRQTGQPTIPSTIGDELESNPFMRVDLPAIQEKMGFNSPVEALGELRKVKDNWRG

> H. sapiens GLYII (Accession No: NP005317)

MVVGRGLLGRRSLAALGAACARRGLGPALLGVFCHTDLRKNLTVDEGTMKVEVLPALTDNYMYLVIDDETKEAAIVDPVQPQKVVDAARKHGVKLTTVLTTHHHWDHAGGNEKLVKLESGLKVYGGDDRIGALTHKITHLSTLQVGSLNVKCLATPCHTSGHICYFVSKPGGSEPPAVFTGDTLFVAGCGKFYEGTADEMCKALLEVLGRLPPDTRVYCGHEYTINNLKFARHVEPGNAAIREKLAWAKEKYSIGEPTVPSTLAEEFTYNPFMRVREKTVQQHAGETDPVTTMRAVRREKDQFKMPRD

>Brassica juncea GLYII (Accession No: AY185202)

MLSKACSLVASSLPRCSSSAAPTIREAAVVAAKRAQENGLGKPLLYGIGTLLVMPLRTLHGVGRMFGAGRFLCNMTSVSSSLQIELVPCLQDNYAYILHDVDTGTVGVVDPSEATPIINALEKRNQNLTYILNTHHHYDHTGGNLELKAKYGAKVIGSAKDRDRIPGIDITLSEGDTWMFAGHQVLVMETPGHTSGHVCYHFPGSGAIFTGDTLFSLSCGKLFEGTPQQMYSSLQKIIALPDETRVYCGHEYTLSNSKFALSIEPGNKDLQEYAANAADLRKRNTPTVPTTIGREKQCNPFLRTSSPEIKNTLSIPDHFDDARVLEVVRRAKDNF
