# Supplementary material for: Genome-wide analysis of glyoxalase-like gene families in grape (Vitis vinifera L.) and their expression profiling in response to downy mildew infection
Source: BMC Genomics. 2019 May 9;20:362. doi: 10.1186/s12864-019-5733-y (PMC6509763; doi:10.1186/s12864-019-5733-y)
Supplement: Supplementary file 15 — Amino acid sequences of DJ-1/PfpI domains and full length sequences of DJ-1 proteins used for phylogenetic analysis. (DOCX 25 kb) [file 12864_2019_5733_MOESM15_ESM.docx]

**Additional file 15:** Amino acid sequences of DJ-1/PfpI domains and full length sequences of DJ-1 proteins used for phylogenetic analysis

**1. DJ-1/PfpI domains**

>VvGLYIII-like1_C

ANGSEEIEVVTVVDILRRAKVDVVVASVEKSLQILASRGIKLIADKSIDNAAESIYDLIILPGGIAGAERLHKSKVLKKMLKEQGSAGRIYGAICSSPTVLHRQGLLKGKRATAHPSVASKLTNEVVEGARVVIDGKLITSRGLATAIEFALAIVSKLFSHARARSVAEGLVFEYPKS

>VvGLYIII-like2_C

GDYMEDYEVMVPFQSFQALECHVDAVCPKKKAGETCPTAIHDFEGDQTYSEKPGHDFTLTATFEDLNIPSYDALVIPGGRAPEYLALNEKVIALVKEFMEAGKPVASICHGQQILAAAGVLKGKKCTAYPAVKLNVVLSGATWLEPEPIDRCFTDGNLVTGAAWPGHPEFISQLMTLLGIQVLF

>VvGLYIII-like3_C

ANGTEEMEAVIIIDFLRRAKANVVVASVEDKLEIVASRKVKLVADVLLDEAVKLSYDLIVLPGGLGGAQAFASSEKLVNLLKNQRESNKPYGAICASPALVLEPHGLLKGKKATAFPALCSKLSDQSEIENRVLVDGNLITSRGPGTSMEFALAIIEKFFGHGKALELAKVMLFSSQ

>AtDJ1-a_C

AEESEEIEAIALVDILRRAKANVVIAAVGNSLEVEGSRKAKLVAEVLLDEVAEKSFDLIVLPGGLNGAQRFASCEKLVNMLRKQAEANKPYGGICASPAYVFEPNGLLKGKKATTHPVVSDKLSDKSHIEHRVVVDGNVITSRAPGTAMEFSLAIVEKFYGREKALQLGKATLV

>AtDJ1-b_C

ADGSEEMEAVAIIDVLKRAKANVVVAALGNSLEVVASRKVKLVADVLLDEAEKNSYDLIVLPGGLGGAEAFASSEKLVNMLKKQAESNKPYGAICASPALVFEPHGLLKGKKATAFPAMCSKLTDQSHIEHRVLVDGNLITSRGPGTSLEFALAIVEKFYGREKGLQLSKATLV

>AtDJ1-d_C

GDYMEDYEVKVPFQSLQALGCQVDAVCPEKKAGDRCPTAIHDFEGDQTYSEKPGHTFALTTNFDDLVSSSYDALVIPGGRAPEYLALNEHVLNIVKEFMNSEKPVASICHGQQILAAAGVLKGRKCTAYPAVKLNVVLGGGTWLEPDPIDRCFTDGNLVTGAAWPGHPEFVSQLMALLGIQVSF

>OsDJ-1B_C

ANGSEEMEALNLIDILRRAGANVTVASVEDKLQVVTRRHKFNLIADIMVEEAAKREFDLIVMPGGLPGAQKLSSTKVLVDLLKKQAESNKPYGAICASPAYVLEPHGLLKGKKATSFPPMAHLLTDQSACDSRVVVDGNLITSKAPGSATEFALAIVEKLFGREKAVSIAKELIFM

>OsDJ-1C_C

GDYMEDYEVMVPFQSLQALGCHVDAVCPDKGAGEKCPTAIHDFEGDQTYSEKPGHDFALTASFDNVDASSYDALVIPGGRAPEYLALNDKVISLVKGFMDKAKPVASICHGQQILSAAGVLQGRKCTAYPAVKLNVVLGGATWLEPNPIDRCFTDGNLVTGAAWPGHPEFISQLMALLGIKVSF

>OsDJ-1D_C

ANGSEEMEIIMLTDVLRRANVNVVLASVEKSTSIVGSQRMRIVADKCISDASALEYDLIILPGGPAGAERLHKSSVLKKLLKEQKQTGRMYGGICSSPVILQKQGLLQDKTVTAHPSIVNQLTCEVIDRSKVVIDGNLITGMGLGTVIDFSLAIIKKFFGHGRAKGVANGMVFEYPKS

>OsDJ-1E_C

ANGTEEMEATMIIDILRRAKANVVVASLEETLEIVASRKVKMVADVLLDDALKQQYDLILLPGGLGGAQAYAKSDKLIGLIKKQAEANKLYGAICASPAIALEPHGLLKGKKATSFPGMWNKLSDQSECKNRVVVDGNLITSQGPGTSMEFSLAIVEKLFGRERAVELAKTMVFM

>GmDJ-1B.1_C

DDHAAKEFNKVEWSVGHHTPSVLVPVAHGSEEIEVVTVVDILRRAKAKVIVASVEKSLEVLASQGTKIVADILIGDAQESAHDLIILPGGTAGAQRLSKSRILKKLLKEQNSAERIYGAVCSSLAILQKQGLLKDKRATAHASTLDKLKDKEINGAKVVIDGKLITSEGLATVTDFALAIVSKLFGNGRARSVAEGLVFEYPKK

>GmDJ-1B.2_C

DDHAAKEFNKVEWSVGHHTPSVLVPVAHGSEEIEVVTVVDILRRAKAKVIVASVEKSLEVLASQGTKIVADILIGDAQESAHDLIILPGGTAGAQRLSKSRILKKLLKEQNSAERIYGAVCSSLAILQKQGLLKDKRATAHASTLDKLKDKEINGAKVVIDGKLITSEGLATVTDFALAIVSKLFGNGRARSVAEGLVFEYPKK

>GmDJ-1D.1_C

HDDEHTFKEFNPVQWTSDNPPKILVPIANGSEEMEAVIIIDILRRAKAKVVVASVEDKLEIVASRKVKLEADMLLDEAAKLSYDLIVLPGGLGGAQTFANSETLVSLLKKQRESNIYYGAICASPALVLEPHGLLKGKKATAFPVMCNKLSDQSEVENRVVVDGNLITSRGPGTSIEFALAIVEKLFGRKLALELAKAVVFARP

>GmDJ-1E.1_C

HDDEHTFKEFNSVQWTSDNPPKILVPIANGSEEMEAVIIIDILRRAKAKVVVASVEDKLEIVASRKVKLEADMLLDEATKLSYDLIVLPGGLGGAQTFANSETLVSLLKKQRESNKYYGAICASPALVLEPHGLLKGKKATAFPVMCDKLSDQSEVENRVVVDGNLITSRGPGTSIEFALAIVEKLFGRKLALELANAVVFARP

>GmDJ-1F.1_C

YMEDYEVKVPFQSLQALGCHVDAVCPSKKAGDTCPTAVHDFEGDQTYSEKPGHTFALTATFDDVDPSGYDALVIPGGRAPEYLALNESVIALVKYFFENKKPVASICHGQQILSAAGVLKGRKCTAYPAVKLNVVLSGATWLEPDPISRCFTDGNLVTGAAWPGHPEFIAQLIALLGIQVSF

>MtDJ-1A_C

ANGTEEMEAVIIVDILRRAKANVVVASVEDKLEIEASRKVKLQADVLLDEAAKTSYDLIVLPGGIGGAQAFANSETLVNLLKKQRESNKYYGAICASPALALEPHGLLKGKKATGFPAMCSKLSDQSEVENRVVIDGNLITSRGPGTSIEFALVIVEKLFGRKLALEIANATVFASP

>MtDJ-1D_C

GDYMEDYEVKVPFQSLQALGCHVDAVCPSKKAGDTCPTAVHDFEGDQTYSEKPGHNFALTATFDDVDPSGYDALVIPGGRSPEYLSLNEAVIALVKHFMENKKPVASICHGQQILAAAGVLKGRKCTAYPAVKLNVVLSGATWLEPDPISRCFTDGNLVTGAAWPGHPEFIAQLMALLGIQVSF

>VvGLYIII-like1_N

MKCLSLSPLLSPPSLSFSSSIKTPFLVALTSTPSKTHTPKRSSKSAKTLFPTTTTSLPPKKVLVPIGYGTEEMEAVILVDVLRRAGANVVVASVEPQLEIEASSGTRLVADTSISTCSDEIFDLIALPGGMPGSARLRDSEILRKITSKHAEEKRLYGAICAAPAITLQPWGLLRRKQMTCHPAFMDKLPTFRAVKSNLQVSGELTTSRGPGTAFEFALALVDQLFGESVAKEVGELLLMRTA

>VvGLYIII-like2_N

MAKSVLILCGDYMEDYEVMVPFQALLAYGVSVHAVCPGKKAGDVCRTAVHQGLGHQTYSESRGHNFTVNATFDEVDASKYDGLVIPGGRAPEYLAMNESVLDLVRKFFSSGKPIASICHGQLILAASGSVRGRKCTAYPAVGPALIAAGAHWVEPETMSACVIDGNLITAATYIGHPGFIQLFVKALGGTITGSDKRILFLCGD

>VvGLYIII-like3_N

MALRHLTPLSPLSPFTRIPPRRCFTQKPFSLSVSASMGSSSRKVLVPIAHGSEPMEAVIIIDVLRRAGADVTVASVEKRLQVDACHGVKIVADALISDCADTGFDLISLPGGMPGAATLRDCGMLESMVKKHAADGQLYAGICAAPAVALGSWGLMKGLKATCYPSFMEQLSSTATTVESRVQQDGKVVTSRGPGTTMEFSVSLVEQLYGKEKANEVSGPLVMCSN

>AtDJ1-a_N

MASFTKTVLIPIAHGTEPLEAVAMITVLRRGGADVTVASVETQVGVDACHGIKMVADTLLSDITDSVFDLIVLPGGLPGGETLKNCKSLENMVKKQDSDGRLNAAICCAPALALGTWGLLEGKKATGYPVFMEKLAATCATAVESRVQIDGRIVTSRGPGTTIEFSITLIEQLFGKEKADEVSSILLLRPN

>AtDJ1-b_N

MASSSLCHRYFNKITVTPFFNTKKLHHYSPRRISLRVNRRSFSISATMSSSTKKVLIPVAHGTEPFEAVVMIDVLRRGGADVTVASVENQVGVDACHGIKMVADTLLSDITDSVFDLIMLPGGLPGGETLKNCKPLEKMVKKQDTDGRLNAAICCAPALAFGTWGLLEGKKATCYPVFMEKLAACATAVESRVEIDGKIVTSRGPGTTMEFSVTLVEQLLGKEKAVEVSGPLVMRPN

>AtDJ1-d_N

MANSRTVLILCGDYMEDYEVMVPFQALQAFGITVHTVCPGKKAGDSCPTAVHDFCGHQTYFESRGHNFTLNATFDEVDLSKYDGLVIPGGRAPEYLALTASVVELVKEFSRSGKPIASICHGQLILAAADTVNGRKCTAYATVGPSLVAAGAKWVEPITPDVCVVDGSLITAATYEGHPEFIQLFVKALGGKITGANKRILFLCGD

>OsDJ-1B_N

MAMAAASASAMARRAASWPRLLLLSRAFAAAAAEPKRVLVPVADGTEPVEAAATADVLNRAGARVTVATADPAGDDRGLLVEAAFGVKLVADGRVADLEGEAFDLIALPGGMPGSANLRDCKVLEKMVKKQAEQGGLYAAICATPAVTLAHWGLLKGLKATCYPSFMEKFTAEIIPVNSRVVVDRNAVTSQGPATAIEYALALVEQLYGKEKSEEVAGPLYVRPQ

>OsDJ-1C_N

MAPKKVLLLCGDYMEDYEAMVPFQALQAYGVSVDAACPGKKAGDSCRTAVHQGIGHQTYAESRGHNFALNASFDEVNINEYDGLVIPGGRAPEYLAMDEKVLDLVRKFSDAKKPIASVCHGQLILAAAGVVQNRKCTAYPAVKPVLVAAGAKWEEADTMDKCTVDGNLVTAVAYDAHPEFISLFVKALGGSVTGSNKRILFLCGD

>OsDJ-1D_N

MLPSSRYLLAPAPLPAMVVRPPPPHPPSRGTSPLARPPLCRAMARAAPSLSAAASTAASSSTTPAKKKVLLPIAMGTEEMEAVILAGVLRRAGADVTLASVEDGLEVEASRGSHIVADKRIAACADQVFDLVALPGGMPGSVRLRDSVILQRITVRQAEEKRLYGAICAAPAVVLMPWGLHKRKKITCHPSFIEDLPTFRTVESNVQVSGELTTSRGPGTAFQFALSFVEQLFGPCKAEDMDNTLLTKVD

>OsDJ-1E_N

MASPPAKKVLVPIASGTEPMEAVITVDVLRRAGADVSVASVDPGSAQVGGAWGVKLAADALLDDLADAEFDLISLPGGMPGSSNLRDCKLLENMVKKHAGKGKLYAAICAAPAVALGSWGLLNGLKATCYPSFMDKLPSEVNAVESRVQIDGNCVTSRGPGTAMEYSVVLVEQLYGKEKADEVAGPMVMRPQ

>GmDJ-1B.1_N

MSLLLLPQPPTPLSTVTFSAAARAPFAAVTPPRPRTLTPKPALSLSAPITTTAPNNAIPPKKVLVPIGLGTEEMEAVIMIHVLRRAGADVTVASVEPQLQVEAAGGTKLVADTDISACSDQVFDLVALPWQGGMPGSARLRDCDVLRKITCRQAEENRLYGAICAAPAVTLLPWGLLKKKKITCHPAFYDRLPRFWAVKSNLQVSRGLTTSRGPGTTYQFALSLAEQLFGDSVANEVAESMFMRTD

>GmDJ-1B.2_N

MSLLLLPQPPTPLSTVTFSAAARAPFAAVTPPRPRTLTPKPALSLSAPITTTAPNNAIPPKKVLVPIGLGTEEMEAVIMIHVLRRAGADVTVASVEPQLQVEAAGGTKLVADTDISACSDQVFDLVALPGGMPGSARLRDCDVLRKITCRQAEENRLYGAICAAPAVTLLPWGLLKKKKITCHPAFYDRLPRFWAVKSNLQVSRGLTTSRGPGTTYQFALSLAEQLFGDSVANEVAESMFMRTD

>GmDJ-1D.1_N

MALRHLRFFPHTLPLTLTPTPNPNNSNRFSFFTPSLSSTTLMATAHKVLVPIADGTEPMEAVITIDVLRRSGADVTVASASDNLAVQALHGVKIIADAPVRDVAATSFDLVALPGGLQGVENLRDCKVLEGLVKKHVEDGRLYAAVCAAPAVVLGPWGLLNGKKATCYPALMEKLAAYAAATSESRVQVDGRVVTSRAPGTTMEFAITLIEQLIGKEKADEVAGPLVMHSN

>GmDJ-1E.1_N

MALRHLRIFPHTLPLTLTPKPKLNNSNRFSFFTSSLSLSSTTLMATAHKVLVPIADGTEPMEAVIIIDVLRRSGADVTVASSSANLAVQALHGVKIIADASVSDVAATAFDLVALPGGLQGDENLRDCKVLEGFVKKHVEDGRLYAAVCAAPAVVLGPWGLLNGKKATCYPALMEKLAAYVAATSESRVQVDGTVVTSRAPGTTMEFAIALIEQLIGKEKAYEVAGPLVMRSN

>GmDJ-1F.1_N

MAPKKVLLLCGDFMEDYEAMVPFQALQAFGLAVDAVCPGKKSGDVCRTAVHVLAGAQTYSETVGHNFSLNATFDEVDAASYDGLWVPGGRAPEYLAHVPGVVELVTKFVSLGKQIASICHGQLILAAAGVVKGRTCTAFPPVKPVLVAAGAHWVEPDTEAATVVDGDLITAATYEGHPELIRHFVKALGGKISGFDKKILFICGD

>MtDJ-1A_N

MALSHIRFFPHTLPSTNFTPKLKLNHNRFFFSPSRSSSSSSSTITAMASNARKVLVPIADGTEPMEAVITIDVLRRSGADVTVASAANRLSVQALHGVKIIADASVSDVVNTAFDLVALPGGVPGVDNLRDSAVLEGLVKKHVEDGKLYAAVCAAPAVVLGPWGLLKGLKATGHPSFMEKLSSYTTSVESRVQLDGRVVTSRAPGTTMEFGVALVEQLLGKEKADEVAGPLVMRSN

>MtDJ-1D_N

MAPKRVLLLCGDFMEDYEGMVPFQALQAFGVSVDAVCPGKKSGDVCRTAVHILSGGQTYTETVGHNFTLNATFDEVDHTSYDGLWLPGGRAPEYLAHIPSVVELVTKFVKSGKEIACICHGHLILAAAGVVEGRKCTAFPPVKPVLVAAGAHWVEPDTMSTTVVDGNLITAPTYEGHPELLRHFLKALGGKISGSDKKILFICGD

**2. Full length protein sequences**

>HumanDJ-1 (Accession No: 1J42A)

MASKRALVILAKGAEEMETVIPVDVMRRAGIKVTVAGLAGKDPVQCSRDVVICPDASLEDAKKEGPYDVVVLPGGNLGAQNLSESAAVKEILKEQENRKGLIAAICAGPTALLAHEIGCGSKVTTHPLAKDKMMNGGHYTYSENRVEKDGLILTSRGPGTSFEFALAIVEALNGKEVAAQVKAPLVLKD

>C.elegansDJR-1.1 (Accession No: NP493696)

MAQKSALIILAAEGAEEMEVIITGDVLARGEIRVVYAGLDGAEPVKCARGAHIVPDVKLEDVETEKFDIVILPGGQPGSNTLAESLLVRDVLKSQVESGGLIGAICAAPIALLSHGVKAELVTSHPSVKEKLEKGGYKYSEDRVVVSGKIITSRGPGTAFEFALKIVELLEGKDKATSLIAPMLLKL

>C.elegansDJR-1.2 (Accession No: NP504132)

MAAQKSALILLPPEDAEEIEVIVTGDVLVRGGLQVLYAGSSTEPVKCAKGARIVPDVALKDVKNKTFDIIIIPGGPGCSKLAECPVIGELLKTQVKSGGLIGAICAGPTVLLAHGIVAERVTCHYTVKDKMTEGGYKYLDDNVVISDRVITSKGPGTAFEFALKIVETLEGPEKTNSLLKPLCLAK

>DrosophilaDJ-1 (Accession No: NP651825)

MSKSALVILAPGAEEMEFIIAADVLRRAGIKVTVAGLNGGEAVKCSRDVQILPDTSLAQVASDKFDVVVLPGGLGGSNAMGESSLVGDLLRSQESGGGLIAAICAAPTVLAKHGVASGKSLTSYPSMKPQLVNNYSYVDDKTVVKDGNLITSRGPGTAYEFALKIAEELAGKEKVQEVAKGLLVAYN

>E.coliHsp31 (Accession No: WP000218214)

MTVQTSKNPQVDIAEDNAFFPSEYSLSQYTSPVSDLDGVDYPKPYRGKHKILVIAADERYLPTDNGKLFSTGNHPIETLLPLYHLHAAGFEFEVATISGLMTKFEYWAMPHKDEKVMPFFEQHKSLFRNPKKLADVVASLNADSEYAAIFVPGGHGALIGLPESQDVAAALQWAIKNDRFVISLCHGPAAFLALRHGDNPLNGYSICAFPDAADKQTPEIGYMPGHLTWYFGEELKKMGMNIINDDITGRVHKDRKVLTGDSPFAANALGKLAAQEMLAAYAG

>MouseDJ-1 (Accession No: NP476484)

MASKRALVILAKGAEEMETVIPVDIMRRAGIKVTVAGLAGKDPVQCSRDVVICPDTSLEEAKTQGPYDVVVLPGGNLGAQNLSESALVKEILKEQENRKGLIAAICAGPTALLAHEVGFGCKVTSHPLAKDKMMNGSHYSYSESRVEKDGLILTSRGPGTSFEFALAIVEALSGKDMANQVKAPLVLKD

>VvGLYIII-like1 (Accession No: XP_002282255)

MKCLSLSPLLSPPSLSFSSSIKTPFLVALTSTPSKTHTPKRSSKSAKTLFPTTTTSLPPKKVLVPIGYGTEEMEAVILVDVLRRAGANVVVASVEPQLEIEASSGTRLVADTSISTCSDEIFDLIALPGGMPGSARLRDSEILRKITSKHAEEKRLYGAICAAPAITLQPWGLLRRKQMTCHPAFMDKLPTFRAVKSNLQVSGELTTSRGPGTAFEFALALVDQLFGESVAKEVGELLLMRTAEDNHKKEEFNEVEWSVDHSPHVLVPVANGSEEIEVVTVVDILRRAKVDVVVASVEKSLQILASRGIKLIADKSIDNAAESIYDLIILPGGIAGAERLHKSKVLKKMLKEQGSAGRIYGAICSSPTVLHRQGLLKGKRATAHPSVASKLTNEVVEGARVVIDGKLITSRGLATAIEFALAIVSKLFSHARARSVAEGLVFEYPKS

>VvGLYIII-like2 (Accession No: XP_010648913)

MAKSVLILCGDYMEDYEVMVPFQALLAYGVSVHAVCPGKKAGDVCRTAVHQGLGHQTYSESRGHNFTVNATFDEVDASKYDGLVIPGGRAPEYLAMNESVLDLVRKFFSSGKPIASICHGQLILAASGSVRGRKCTAYPAVGPALIAAGAHWVEPETMSACVIDGNLITAATYIGHPGFIQLFVKALGGTITGSDKRILFLCGDYMEDYEVMVPFQSFQALECHVDAVCPKKKAGETCPTAIHDFEGDQTYSEKPGHDFTLTATFEDLNIPSYDALVIPGGRAPEYLALNEKVIALVKEFMEAGKPVASICHGQQILAAAGVLKGKKCTAYPAVKLNVVLSGATWLEPEPIDRCFTDGNLVTGAAWPGHPEFISQLMTLLGIQVLF

>VvGLYIII-like3 (Accession No: XP_010644129)

MALRHLTPLSPLSPFTRIPPRRCFTQKPFSLSVSASMGSSSRKVLVPIAHGSEPMEAVIIIDVLRRAGADVTVASVEKRLQVDACHGVKIVADALISDCADTGFDLISLPGGMPGAATLRDCGMLESMVKKHAADGQLYAGICAAPAVALGSWGLMKGLKATCYPSFMEQLSSTATTVESRVQQDGKVVTSRGPGTTMEFSVSLVEQLYGKEKANEVSGPLVMCSNLGDKFIMAELNPIDWKCDNPQILVPIANGTEEMEAVIIIDFLRRAKANVVVASVEDKLEIVASRKVKLVADVLLDEAVKLSYDLIVLPGGLGGAQAFASSEKLVNLLKNQRESNKPYGAICASPALVLEPHGLLKGKKATAFPALCSKLSDQSEIENRVLVDGNLITSRGPGTSMEFALAIIEKFFGHGKALELAKVMLFSSQ

>AtDJ-1A (Accession No: AT3G14990)

MASFTKTVLIPIAHGTEPLEAVAMITVLRRGGADVTVASVETQVGVDACHGIKMVADTLLSDITDSVFDLIVLPGGLPGGETLKNCKSLENMVKKQDSDGRLNAAICCAPALALGTWGLLEGKKATGYPVFMEKLAATCATAVESRVQIDGRIVTSRGPGTTIEFSITLIEQLFGKEKADEVSSILLLRPNPGEEFTFTELNQTNWSFEDTPQILVPIAEESEEIEAIALVDILRRAKANVVIAAVGNSLEVEGSRKAKLVAEVLLDEVAEKSFDLIVLPGGLNGAQRFASCEKLVNMLRKQAEANKPYGGICASPAYVFEPNGLLKGKKATTHPVVSDKLSDKSHIEHRVVVDGNVITSRAPGTAMEFSLAIVEKFYGREKALQLGKATLV

>AtDJ-1B (Accession No: AT1G53280)

MASSSLCHRYFNKITVTPFFNTKKLHHYSPRRISLRVNRRSFSISATMSSSTKKVLIPVAHGTEPFEAVVMIDVLRRGGADVTVASVENQVGVDACHGIKMVADTLLSDITDSVFDLIMLPGGLPGGETLKNCKPLEKMVKKQDTDGRLNAAICCAPALAFGTWGLLEGKKATCYPVFMEKLAACATAVESRVEIDGKIVTSRGPGTTMEFSVTLVEQLLGKEKAVEVSGPLVMRPNPGDEYTITELNQVSWSFEGTPQILVPIADGSEEMEAVAIIDVLKRAKANVVVAALGNSLEVVASRKVKLVADVLLDEAEKNSYDLIVLPGGLGGAEAFASSEKLVNMLKKQAESNKPYGAICASPALVFEPHGLLKGKKATAFPAMCSKLTDQSHIEHRVLVDGNLITSRGPGTSLEFALAIVEKFYGREKGLQLSKATLV

>AtDJ-1D (Accession No: AT3G02720)

MANSRTVLILCGDYMEDYEVMVPFQALQAFGITVHTVCPGKKAGDSCPTAVHDFCGHQTYFESRGHNFTLNATFDEVDLSKYDGLVIPGGRAPEYLALTASVVELVKEFSRSGKPIASICHGQLILAAADTVNGRKCTAYATVGPSLVAAGAKWVEPITPDVCVVDGSLITAATYEGHPEFIQLFVKALGGKITGANKRILFLCGDYMEDYEVKVPFQSLQALGCQVDAVCPEKKAGDRCPTAIHDFEGDQTYSEKPGHTFALTTNFDDLVSSSYDALVIPGGRAPEYLALNEHVLNIVKEFMNSEKPVASICHGQQILAAAGVLKGRKCTAYPAVKLNVVLGGGTWLEPDPIDRCFTDGNLVTGAAWPGHPEFVSQLMALLGIQVSF

>OsDJ-1B (Accession No: LOC_Os01g11880)

MAMAAASASAMARRAASWPRLLLLSRAFAAAAAEPKRVLVPVADGTEPVEAAATADVLNRAGARVTVATADPAGDDRGLLVEAAFGVKLVADGRVADLEGEAFDLIALPGGMPGSANLRDCKVLEKMVKKQAEQGGLYAAICATPAVTLAHWGLLKGLKATCYPSFMEKFTAEIIPVNSRVVVDRNAVTSQGPATAIEYALALVEQLYGKEKSEEVAGPLYVRPQPGVDYVIDEFNSVEWKCSGTPQVLVPVANGSEEMEALNLIDILRRAGANVTVASVEDKLQVVTRRHKFNLIADIMVEEAAKREFDLIVMPGGLPGAQKLSSTKVLVDLLKKQAESNKPYGAICASPAYVLEPHGLLKGKKATSFPPMAHLLTDQSACDSRVVVDGNLITSKAPGSATEFALAIVEKLFGREKAVSIAKELIFM

>OsDJ-1C (Accession No: LOC_Os04g57590)

MAPKKVLLLCGDYMEDYEAMVPFQALQAYGVSVDAACPGKKAGDSCRTAVHQGIGHQTYAESRGHNFALNASFDEVNINEYDGLVIPGGRAPEYLAMDEKVLDLVRKFSDAKKPIASVCHGQLILAAAGVVQNRKCTAYPAVKPVLVAAGAKWEEADTMDKCTVDGNLVTAVAYDAHPEFISLFVKALGGSVTGSNKRILFLCGDYMEDYEVMVPFQSLQALGCHVDAVCPDKGAGEKCPTAIHDFEGDQTYSEKPGHDFALTASFDNVDASSYDALVIPGGRAPEYLALNDKVISLVKGFMDKAKPVASICHGQQILSAAGVLQGRKCTAYPAVKLNVVLGGATWLEPNPIDRCFTDGNLVTGAAWPGHPEFISQLMALLGIKVSF

>OsDJ-1D (Accession No: LOC_Os05g44330)

MLPSSRYLLAPAPLPAMVVRPPPPHPPSRGTSPLARPPLCRAMARAAPSLSAAASTAASSSTTPAKKKVLLPIAMGTEEMEAVILAGVLRRAGADVTLASVEDGLEVEASRGSHIVADKRIAACADQVFDLVALPGGMPGSVRLRDSVILQRITVRQAEEKRLYGAICAAPAVVLMPWGLHKRKKITCHPSFIEDLPTFRTVESNVQVSGELTTSRGPGTAFQFALSFVEQLFGPCKAEDMDNTLLTKVDDNLERSIEVNEIEWSSDHNPHVLIPIANGSEEMEIIMLTDVLRRANVNVVLASVEKSTSIVGSQRMRIVADKCISDASALEYDLIILPGGPAGAERLHKSSVLKKLLKEQKQTGRMYGGICSSPVILQKQGLLQDKTVTAHPSIVNQLTCEVIDRSKVVIDGNLITGMGLGTVIDFSLAIIKKFFGHGRAKGVANGMVFEYPKS

>OsDJ-1E (Accession No: LOC_Os06g34040)

MATRPLAASTLLPPLRFCSPLKTPPPSPPPPHLRRLQTLTRALASSSSAMASPPAKKVLVPIASGTEPMEAVITVDVLRRAGADVSVASVDPGSAQVGGAWGVKLAADALLDDLADAEFDLISLPGGMPGSSNLRDCKLLENMVKKHAGKGKLYAAICAAPAVALGSWGLLNGLKATCYPSFMDKLPSEVNAVESRVQIDGNCVTSRGPGTAMEYSVVLVEQLYGKEKADEVAGPMVMRPQHGVEFSLKELNSTSWNVGETPQILVPIANGTEEMEATMIIDILRRAKANVVVASLEETLEIVASRKVKMVADVLLDDALKQQYDLILLPGGLGGAQAYAKSDKLIGLIKKQAEANKLYGAICASPAIALEPHGLLKGKKATSFPGMWNKLSDQSECKNRVVVDGNLITSQGPGTSMEFSLAIVEKLFGRERAVELAKTMVFM

>GmDJ-1B.1 (Accession No: Glyma.07G213200)

MSLLLLPQPPTPLSTVTFSAAARAPFAAVTPPRPRTLTPKPALSLSAPITTTAPNNAIPPKKVLVPIGLGTEEMEAVIMIHVLRRAGADVTVASVEPQLQVEAAGGTKLVADTDISACSDQVFDLVALPWQGGMPGSARLRDCDVLRKITCRQAEENRLYGAICAAPAVTLLPWGLLKKKKITCHPAFYDRLPRFWAVKSNLQVSRGLTTSRGPGTTYQFALSLAEQLFGDSVANEVAESMFMRTDDDHAAKEFNKVEWSVGHHTPSVLVPVAHGSEEIEVVTVVDILRRAKAKVIVASVEKSLEVLASQGTKIVADILIGDAQESAHDLIILPGGTAGAQRLSKSRILKKLLKEQNSAERIYGAVCSSLAILQKQGLLKDKRATAHASTLDKLKDKEINGAKVVIDGKLITSEGLATVTDFALAIVSKLFGNGRARSVAEGLVFEYPKK

>GmDJ-1B.2 (Accession No: Glyma.07G213200)

MSLLLLPQPPTPLSTVTFSAAARAPFAAVTPPRPRTLTPKPALSLSAPITTTAPNNAIPPKKVLVPIGLGTEEMEAVIMIHVLRRAGADVTVASVEPQLQVEAAGGTKLVADTDISACSDQVFDLVALPGGMPGSARLRDCDVLRKITCRQAEENRLYGAICAAPAVTLLPWGLLKKKKITCHPAFYDRLPRFWAVKSNLQVSRGLTTSRGPGTTYQFALSLAEQLFGDSVANEVAESMFMRTDDDHAAKEFNKVEWSVGHHTPSVLVPVAHGSEEIEVVTVVDILRRAKAKVIVASVEKSLEVLASQGTKIVADILIGDAQESAHDLIILPGGTAGAQRLSKSRILKKLLKEQNSAERIYGAVCSSLAILQKQGLLKDKRATAHASTLDKLKDKEINGAKVVIDGKLITSEGLATVTDFALAIVSKLFGNGRARSVAEGLVFEYPKK

>GmDJ-1D.1 (Accession No: Glyma.12G228600)

MALRHLRFFPHTLPLTLTPTPNPNNSNRFSFFTPSLSSTTLMATAHKVLVPIADGTEPMEAVITIDVLRRSGADVTVASASDNLAVQALHGVKIIADAPVRDVAATSFDLVALPGGLQGVENLRDCKVLEGLVKKHVEDGRLYAAVCAAPAVVLGPWGLLNGKKATCYPALMEKLAAYAAATSESRVQVDGRVVTSRAPGTTMEFAITLIEQLIGKEKADEVAGPLVMHSNHDDEHTFKEFNPVQWTSDNPPKILVPIANGSEEMEAVIIIDILRRAKAKVVVASVEDKLEIVASRKVKLEADMLLDEAAKLSYDLIVLPGGLGGAQTFANSETLVSLLKKQRESNIYYGAICASPALVLEPHGLLKGKKATAFPVMCNKLSDQSEVENRVVVDGNLITSRGPGTSIEFALAIVEKLFGRKLALELAKAVVFARP

>GmDJ-1E.1 (Accession No: Glyma.13G271200)

MALRHLRIFPHTLPLTLTPKPKLNNSNRFSFFTSSLSLSSTTLMATAHKVLVPIADGTEPMEAVIIIDVLRRSGADVTVASSSANLAVQALHGVKIIADASVSDVAATAFDLVALPGGLQGDENLRDCKVLEGFVKKHVEDGRLYAAVCAAPAVVLGPWGLLNGKKATCYPALMEKLAAYVAATSESRVQVDGTVVTSRAPGTTMEFAIALIEQLIGKEKAYEVAGPLVMRSNHDDEHTFKEFNSVQWTSDNPPKILVPIANGSEEMEAVIIIDILRRAKAKVVVASVEDKLEIVASRKVKLEADMLLDEATKLSYDLIVLPGGLGGAQTFANSETLVSLLKKQRESNKYYGAICASPALVLEPHGLLKGKKATAFPVMCDKLSDQSEVENRVVVDGNLITSRGPGTSIEFALAIVEKLFGRKLALELANAVVFARP

>GmDJ-1F.1 (Accession No: Glyma.18G045900)

MAPKKVLLLCGDFMEDYEAMVPFQALQAFGLAVDAVCPGKKSGDVCRTAVHVLAGAQTYSETVGHNFSLNATFDEVDAASYDGLWVPGGRAPEYLAHVPGVVELVTKFVSLGKQIASICHGQLILAAAGVVKGRTCTAFPPVKPVLVAAGAHWVEPDTEAATVVDGDLITAATYEGHPELIRHFVKALGGKISGFDKKILFICGDYMEDYEVKVPFQSLQALGCHVDAVCPSKKAGDTCPTAVHDFEGDQTYSEKPGHTFALTATFDDVDPSGYDALVIPGGRAPEYLALNESVIALVKYFFENKKPVASICHGQQILSAAGVLKGRKCTAYPAVKLNVVLSGATWLEPDPISRCFTDGNLVTGAAWPGHPEFIAQLIALLGIQVSF

>MtDJ-1A (Accession No: Medtr2g078060)

MALSHIRFFPHTLPSTNFTPKLKLNHNRFFFSPSRSSSSSSSTITAMASNARKVLVPIADGTEPMEAVITIDVLRRSGADVTVASAANRLSVQALHGVKIIADASVSDVVNTAFDLVALPGGVPGVDNLRDSAVLEGLVKKHVEDGKLYAAVCAAPAVVLGPWGLLKGLKATGHPSFMEKLSSYTTSVESRVQLDGRVVTSRAPGTTMEFGVALVEQLLGKEKADEVAGPLVMRSNHADEYTFLELNSVQWTFDNPPKILVPIANGTEEMEAVIIVDILRRAKANVVVASVEDKLEIEASRKVKLQADVLLDEAAKTSYDLIVLPGGIGGAQAFANSETLVNLLKKQRESNKYYGAICASPALALEPHGLLKGKKATGFPAMCSKLSDQSEVENRVVIDGNLITSRGPGTSIEFALVIVEKLFGRKLALEIANATVFASP

>MtDJ-1D (Accession No: Medtr3g064140)

MAPKRVLLLCGDFMEDYEGMVPFQALQAFGVSVDAVCPGKKSGDVCRTAVHILSGGQTYTETVGHNFTLNATFDEVDHTSYDGLWLPGGRAPEYLAHIPSVVELVTKFVKSGKEIACICHGHLILAAAGVVEGRKCTAFPPVKPVLVAAGAHWVEPDTMSTTVVDGNLITAPTYEGHPELLRHFLKALGGKISGSDKKILFICGDYMEDYEVKVPFQSLQALGCHVDAVCPSKKAGDTCPTAVHDFEGDQTYSEKPGHNFALTATFDDVDPSGYDALVIPGGRSPEYLSLNEAVIALVKHFMENKKPVASICHGQQILAAAGVLKGRKCTAYPAVKLNVVLSGATWLEPDPISRCFTDGNLVTGAAWPGHPEFIAQLMALLGIQVSF
