# Supplementary material for: Genome-wide analysis of glyoxalase-like gene families in grape (Vitis vinifera L.) and their expression profiling in response to downy mildew infection
Source: BMC Genomics. 2019 May 9;20:362. doi: 10.1186/s12864-019-5733-y (PMC6509763; doi:10.1186/s12864-019-5733-y)
Supplement: Supplementary file 16 — Other sequences and their accession numbers used in this paper. (DOCX 58 kb) [file 12864_2019_5733_MOESM16_ESM.docx]

**Additional file 16:** Other sequences and their accession numbers used in this paper.

**1. CDs sequences of glyoxalase genes from grape**

>VvGLYI-like1 (Accession No. XM_002283932)

ATGAGGACGCTTCCAATGGCAACAACGCCGTTTCAACACCTCTCTTCATCCCCACCTTTTCTCTCCTCTCTCAGATTCCCCACTTCTTCCTTCGCTTCTTCTCTCACTGGTTTCACCTCTTCTCGTCGACTCGCGCTTTTCCACCTTGGCACTGCTATACCTCAATCAGAGTTGTTGGGTGGAAAAACATTGAAGTTATTTAGAATGGAAGGAAACATGCTAGAGGCTGGCACAGCTGGAAATATGGCACAAGCTGCCGTTTCAGAGGGAAATGTACTGGAGTGGGCCAAAACTGACAAGCGAAGAATGCTTCACGTTGTTTACCGTGTGGGAAACTTGGACAAGACTATGAAATTCTATACTGAGTGCTTGGGGATGAAACTATTGAGGAGATGTGATATACCAGAGGAGAGATATGCGAATGCTTTTCTTGGATATGGGCCCGAGGATTCCCATTTTGTTGTTGAACTTACTTACAATTATGGAGTTGACAAAATCGACATTGGAACAGGTTTTGGCCATTTTGGTATTGCAGTTGAAGATGTCGCCAAAACAGTGGATCTGGTAAAGGCAAAGGGCGGAAAAGTTACCAGGGAGCCTGGTCCTGTTAAAGGTGGCAAAACAGTAATTGCATTCGTTGAAGATCCAGATGGTTATAAGTTTGAACTTTTGGAAAGAGAGCCTACACCAGAGCCCTTATGTCAAGTAATGCTTCGGGTGGGCGATCTTGATCGCTCCATAAAGTTTTATGAGAAGGCTTTTGGCATGGAGCTTCTCCGCAAAAGAGATAATCCTGAATACAAGTATACAATTGCTATGATGGGCTATGGTCCTGAAGATAAGAGTGCAGTGCTGGAATTGACTTATAACTATGGGGTCCTGGAATATGACAAAGGAAATGGCTATGCACAGATTGCAATTGGCACAGATGATGTTTATAAGACTGCAGAAGCAATCAGATTGTGTGGGGGGAAGATTACTCGTGAACCTGGGCCCTTGCCAGTTATCAACACTAAGATCACTGCCTGCTTGGACCCTGATGGATGGAAATCGGTCTTTGTTGATAATGCTGATTTTCTCAAGGAATTAGAGTGA

>VvGLYI-like2 (Accession No. XM_002276240)

ATGGCTTCCTGCTCTATTGCCACTTCTCTATCGCGCCTCTCTCTCCTCCGTCTAATCCCCAAACCCTCTTCTTCTTATTCTTCTTCAATTCCTCTTTTTCCTACCACTACTCGGAAGGATCCGAGTAGGTTTCGGTTGTTTTCGGCGTCAATGGCTTCTGAACCCAAGGAATCTCCCTCTAACAATCCGGGCCTCCATTCCAGCCCTGATGAGGCCACTAAAGGATACTTCATGCAACAGACTATGTATCGGATTAAGGACCCTAAAGTCAGTCTCGATTTCTATTCTCGCGTATTGGGAATGTCGCTACTTAAACGGTTGGATTTTCCAGAGATGAAGTTTAGCCTGTACTTTATGGGGTATGAGGATACAGCATCAGCTCCCAGTAATGAAACTGAAAGAATCGTCTGGACCTTTAGTCAAAAGGCTACTATTGAGCTCACACACAACTGGGGTACTGAGAGTGATCCTGACTTCAAAGGTTACCACAATGGGAACTCAGAACCTCGTGGATTTGGACACATAGGCATCACAGTCGATGACACATACAAGGCATGCGAAAGATTTGAACGTCTAGGGGTTGAGTTTGTAAAAAAACCAGATGATGGTAAAATGAAAGGGTTAGCATTCATTAAGGATCCTGATGGCTATTGGATTGAAATCTTTGACCTTAGAAGAATTGGAACTGTGAGTACCACTGCTGCTTGA

>VvGLYI-like3 (Accession No. XM_002273310)

ATGGCTGAGGCTGCACCTGTTGTTCCAAGTGATGAGTTGTTGGAATGGCCAAAGAAAGATAAGCGCCGGTTTCTTCATGTTGTGTATCGAGTTGGTGATCTTGATCGCACCATCAAGTTTTATACGGAGTGTTTCGGGATGAAGCTTTTGCGCAAAAGGGACATTCCAGAAGAGAAATACACCAATGCCTTTCTCGGGTTTGGCCCTGAAGAGACTAATTTTGTGGTGGAGTTAACATACAACTATGGAGTGGACAAGTATGATATTGGAACAGGTTTTGGGCATTTTGCAATTGCAACTCAAGATGTTTATAAGATGGTTGAGGACATTCGGGCCAAGGGTGGGATTATCACAAGGGAACCTGGTCCAGTTAAAGGTGGAAAGAGTGTCATTGCCTTCGCGAAGGATCCTGATGGCTATATTTTTGAGCTCATCCAAAGAGGCCCAACGCCTGAACCACTCTGTCAAGTAATGCTTCGTGTAGGTGATCTGGAAAGGTCTATCAAGTTCTATGAAAAGGCCTTGGGGATGAAGATGGTAAAGAAGACTGACAGACCTGAATACAAGTACTCCATAGCCATGATGGGATATGCAGAGGAACATGAAACAACTGTTCTGGAGTTGACATACAATTATGGTGTCACTGAGTATACCAAAGGAAACGCATATGCACAGGTTGCTATCAGTACTGATGATGTATACAAGAGTGCTGAGGTTGTCAACTTGGTTACCAAAGAGCTGGGAGGAAAGATAACTAGGCAACCAGGACCAATTCCTGGACTCAACACCAAGATCACCTCTTTTCTGGATCCAGATGGCTGGAAGACAGTTCTGGTTGATAATGAAGATTTTCTCAAAGAACTGCACAAGGAAGAGTAA

>VvGLYI-like4 (Accession No. XM_003633073)

ATGGTGAGGATAATCCCCATGGCGACGTCGTTTCGACCTTCTCTTTCCTCTTTCGGATTTTCTACTTCTTCTCGACTTGGTTTTCCGCTTTCCACATTCAATATTTCTCGAACGGTCACTTCGCTACACGTCGGCAGCGCTATTCCGCAGTCCCAAATTTTTGGTCTAAATGCTTCTAAATTGTTAAGAGGAGGAGAGGGCAATGCCATGGGATTTAATGCAACCGGAAATATTGCTCACGCAAGCACTAGTGCTGCCCAAGAAAATGTTCTAGAGTGGGTGAAGAAGGACAAGCGAAGAATGCTCCATGTTGTGTATCGTGTTGGGGATTTGGATAGGACCATAAAATTCTACACAGAGTGCCTAGGGATGAAGCTTTTGCGAAGGCGAGACATACCAGAGGAAAGATACACAAATGCCTTTCTTGGATATGGGCCAGAAGATTCGCACTTTGTTATTGAACTCACATACAATTATGGAGTTGACAAGTATGATATTGGAGCTGGTTTTGGCCATTTTGGTATTGCAGTTGAGGATGTTACTAAGACTGTGGATCTCATAAAGGCTAAGGGTGGCAAAGTAACCCGGGAACCTGGTCCTGTTAAAGGTGGCAGTACAGTTATTGCTTTTATTGAAGATCCTGATGGCTATAAGTTTGAACTTTTGGAAAGGGGACCTACACCTGAGCCATTGTGTCAAGTTATGCTTCGAGTTGGTGATCTTGATCGTTCCATAAATTTTTACGAGAAGGCTTTTGGCATGGAGCTTCTCCGTAAAAGAGATAATCCTGAGTACAAGTATACAATAGCAATGATGGGCTATGGTCCTGAAGATAAAAATGCTGTTCTGGAGCTGACTTACAACTATGGGGTCTCTGAATATGACAAAGGAAATGGTTATGCTCAGATTGCAATCGGCACAGATGATGTCTATAAAACTGCAGAAGCTATTAAACTCTCTGGTGGGAAGATTACCCGAGAACCTGGACCATTACCTGGCATTAACACCAAGATTACTGCATGCGTGGATCCTGATGGTTGGAAGTCGGTTTTTGTTGATAATATTGATTTCCTCAAGGAATTGGATTGA

>VvGLYII-like1 (Accession No. XM_002271759)

ATGCAAGTCATCTCCAAAGCACCCTCAGCCATGGCCTCCTTCTCTTGCTCCTCTAGGGGGGGTAATGGGCTTGGTGTATGGCCAGGCATGAGGCAACTGTGCCTTAGAAAAAGTCTTTTGTATGGACTCATGCGCCTTTTATCAACACCATTCAAAACTTTGCGCACAGCTGGTCGCACACTCAAAGTTGCACAATTTTGCAGTGTTTCCAATACGTCTTCCACATTGCAAATTGAATTGGTACCATGTCTGAAGGACAACTATGCATATCTTTTACATGACGTCGATACAGGCACAGTTGGAGTTGTTGATCCTTCTGAAGCCGTACCTGTTATAGATGCCCTGAGTAGGAAAAACCGAAACCTAACCTATATCCTGAACACTCATCACCATCATGATCACACTGGTGGGAATGCTGAGTTAAAAGCAAGGTACGGTGCAAAGGTAATTGGTTCTGGAATAGACAAGGATCGAATTCCTGGAATTGATATAGTTTTGAAAGATGGAGACAAGTGGATGTTTGCAGGCCATGAGGTGGTTGTAATTGAAACTCCTGGTCATACCCGAGGCCACATTAGCTTCTACTTTCCGGGATCAGGGGCAATTTTTACAGGGGACACTTTGTTCAGTCTATCATGTGGCAAACTTTTCGAAGGAACTCCTGAACAGATGCATTCTTCCCTTAGTAAGATTATGTCTTTGCCAGACGAAACAAATATCTATTGTGGTCATGAATATACATTGAGCAATTCAAAGTTTGCATTATCTATCGAACCCAAGAATGAGGTCCTCCAGTCCTATGCCACCCATGTTGCCCATCTCCGTAGTAAGGGCTTGCCAACGATCCCAACTACACTGAAGATGGAAAAGATGTGCAACCCATTCCTTCGCACTTCAAGCCCAGAAATACGGAAATCGTTAAAGATTACAGCCACAGCAGATGACTCAGAAGCGCTGGGGATCATCCGTGAAGCAAAGGACAACTTTTAA

>VvGLYII-like2 (Accession No. XM_002267435)

ATGAAGATCGTCCCAGTTCCTTGCTTGGAAGACAATTACTCTTACCTGATCATCGATGAGAGTTCTAAAGAAGCAGCAGTTGTTGATCCAGTAGAGCCCCAGAAGGTTCTTCAAGCTGCTTATGAATATGGCGTCCATCTCAAGCTCGTCCTCACCACTCACCATCACTGGGATCACGCCGGCGGAAACGAAAAGATAAAGCAGCTGGTGCCCGGAATCGAAGTGTACGGTGGGTCTGTCGACAACGTCAAGGGTTGCACCCATCCACTCCAAAATGGTGATAAGCTGTCGCTTGGCTCTGATCTCGCCGTTTTGGCTCTTCACACTCCTTGTCATACAAGGGGTCACATAAGTTACTATGTGACAGGCAAAGAGGAAGATGTCCCGGCTGTTTTCACTGGAGACACATTGTTTGTTGCTGGTTGTGGGAAATTTTTTGAAGGCACAGCAGAACAGATGTATCAATCGCTATGTGTAACATTGGCCTCATTGCCAAAGCCAACTAGAGTATACTGTGGCCATGAGTACACAGTTAAGAACTTGCAGTTTGCTCTGACTGTTGAACCAGATAATGTGAGGGTGGGGCAAAAGCTGTCATGGGCACAACATCAACGACAAGCTGGCCTCCCCACCATTCCCTCAACAATTGATGAAGAGATGGAGACTAACCCATTCATGCGGGTTGACCTACCAGAGCTTCAGGAGAGGGTTGGTTGCCAGTCGGCCATAGATGCTCTACAAGAGATAAGGCGGCAGAAGGACAACTGGAGAGGCTGA

>VvGLYIII-like1 (Accession No. XM_002282219)

ATGAAGTGTCTGAGTCTATCGCCGCTTCTCTCACCACCTTCTTTGAGCTTCTCTTCTTCCATCAAGACCCCATTTCTGGTGGCTCTCACCTCCACGCCCTCAAAAACACATACTCCCAAACGCTCCTCGAAATCCGCTAAAACTCTCTTTCCAACCACCACCACCTCTCTTCCTCCCAAAAAGGTTCTGGTGCCTATTGGATATGGAACGGAGGAAATGGAAGCGGTTATTCTAGTCGACGTTCTGCGGCGAGCTGGTGCGAACGTGGTTGTGGCGTCGGTGGAACCCCAGCTCGAGATTGAGGCTTCTAGCGGCACCAGACTGGTTGCTGATACCTCCATCTCAACATGCTCTGATGAAATTTTTGATCTCATAGCTTTGCCGGGAGGAATGCCGGGCTCTGCACGATTGAGGGATAGTGAAATTCTCCGGAAAATTACTAGCAAACACGCTGAGGAAAAAAGGCTGTATGGTGCTATATGTGCTGCTCCAGCCATTACACTTCAACCATGGGGCCTTCTGAGAAGAAAACAGATGACTTGTCACCCAGCATTCATGGACAAGCTTCCAACCTTCAGGGCTGTTAAATCAAATCTACAAGTTTCTGGGGAGCTCACAACAAGCAGAGGCCCCGGAACTGCTTTTGAGTTTGCTTTAGCATTAGTTGATCAACTTTTTGGAGAGTCTGTAGCCAAGGAGGTTGGAGAATTGTTGCTGATGCGTACTGCTGAGGACAATCATAAAAAGGAAGAGTTTAATGAAGTTGAATGGTCAGTTGATCATAGTCCTCATGTTCTTGTCCCAGTTGCAAATGGTTCTGAAGAAATTGAAGTGGTTACAGTTGTAGATATTCTACGGCGAGCAAAGGTGGATGTTGTGGTTGCTTCAGTTGAGAAATCTTTGCAGATTTTAGCATCTCGAGGCATTAAACTTATAGCTGACAAGTCAATTGATAATGCTGCTGAATCAATTTATGACTTGATCATTCTTCCGGGGGGGATTGCTGGTGCTGAGCGGCTGCACAAATCCAAGGTTCTGAAGAAGATGCTCAAAGAACAAGGATCAGCAGGAAGAATATATGGAGCAATCTGCTCCTCGCCAACAGTCTTACATAGACAAGGGTTACTCAAGGGTAAAAGAGCCACCGCTCATCCCTCTGTAGCTAGCAAGCTCACCAATGAAGTAGTGGAGGGAGCTAGGGTAGTTATTGACGGCAAGCTGATTACAAGCAGGGGGCTTGCAACTGCAATAGAATTTGCATTGGCAATTGTTAGCAAGCTTTTTAGTCATGCAAGAGCAAGGAGTGTAGCTGAAGGTCTTGTTTTTGAGTACCCTAAGAGCTAG

>VvGLYIII-like2 (Accession No. XM_010650611)

ATGGCCAAGAGCGTTCTCATCCTCTGCGGCGATTACATGGAAGACTATGAGGTGATGGTTCCCTTTCAAGCGTTGCTGGCCTACGGAGTCTCCGTCCACGCCGTTTGCCCTGGAAAGAAAGCCGGCGACGTCTGCCGGACTGCTGTTCATCAGGGCCTTGGTCATCAGACTTATTCTGAGTCACGGGGTCACAACTTCACAGTCAATGCAACCTTTGATGAGGTTGACGCAAGCAAATATGATGGACTTGTTATTCCAGGAGGGCGGGCTCCAGAATATCTTGCAATGAATGAATCTGTTTTGGACTTGGTACGCAAATTTTTTAGCTCTGGAAAACCAATTGCCTCTATTTGCCATGGGCAGTTAATCTTGGCAGCTTCAGGCTCAGTCAGAGGACGGAAGTGCACAGCTTACCCTGCTGTGGGACCTGCACTCATTGCTGCAGGAGCTCATTGGGTAGAACCTGAGACCATGTCAGCATGTGTTATTGATGGTAACCTTATTACTGCAGCAACTTACATAGGTCATCCTGGGTTCATCCAGCTTTTTGTAAAAGCACTAGGAGGCACCATAACTGGTTCAGATAAACGGATTCTATTTTTGTGTGGGGATTACATGGAAGACTATGAGGTAATGGTCCCTTTTCAATCCTTTCAAGCTCTTGAATGCCATGTTGATGCGGTTTGCCCGAAGAAGAAGGCCGGTGAGACCTGTCCAACTGCTATTCATGATTTTGAAGGTGATCAAACTTACAGTGAGAAGCCAGGCCATGATTTCACTCTAACAGCCACTTTTGAAGATCTAAACATCCCAAGCTATGATGCTCTTGTTATACCTGGAGGCCGAGCCCCAGAATATTTGGCATTAAATGAGAAAGTAATTGCCTTAGTGAAGGAATTCATGGAGGCAGGAAAGCCAGTTGCATCCATTTGCCACGGACAACAGATTTTAGCTGCTGCTGGAGTTCTCAAGGGAAAGAAATGCACTGCATATCCTGCTGTGAAACTCAATGTAGTCTTGTCTGGGGCTACATGGTTAGAACCTGAGCCAATAGATCGCTGTTTCACAGATGGAAATTTGGTAACTGGAGCTGCTTGGCCGGGCCATCCAGAGTTCATCTCTCAGTTGATGACGTTGCTCGGTATCCAGGTCTTGTTCTAG

>VvGLYIII-like3 (Accession No. XM_010645827)

ATGGCTTTACGCCACCTCACTCCACTGAGTCCACTCTCTCCCTTCACACGCATACCTCCACGACGTTGTTTCACTCAGAAGCCCTTCTCCCTCTCCGTCTCGGCATCCATGGGTTCTTCTTCTCGTAAGGTTTTGGTTCCGATCGCCCACGGCTCGGAGCCGATGGAGGCGGTGATCATCATCGATGTGCTCCGGCGAGCTGGCGCCGATGTGACCGTCGCTTCTGTGGAGAAGCGGCTTCAAGTTGATGCGTGCCATGGCGTGAAGATTGTTGCGGATGCCCTAATTTCTGACTGCGCTGATACTGGTTTCGATCTCATTTCGCTGCCGGGAGGAATGCCAGGTGCTGCCACTCTCAGAGATTGTGGCATGCTGGAAAGCATGGTAAAGAAGCATGCTGCTGATGGGCAGCTCTATGCTGGAATCTGTGCGGCACCTGCTGTGGCACTTGGATCATGGGGCTTGATGAAAGGTTTGAAGGCAACCTGTTATCCATCATTCATGGAGCAATTGTCTTCCACTGCAACTACAGTTGAATCAAGAGTCCAACAGGATGGCAAAGTTGTGACGAGTCGTGGACCAGGCACTACTATGGAGTTCTCAGTCTCTTTGGTTGAGCAATTATATGGAAAAGAGAAAGCTAATGAAGTTTCTGGGCCACTGGTGATGTGTTCCAATCTTGGGGATAAATTCATCATGGCTGAGCTAAATCCGATTGATTGGAAGTGTGACAATCCTCAGATTCTTGTGCCTATTGCTAATGGCACAGAAGAAATGGAAGCTGTTATAATCATTGATTTTCTGCGTCGAGCAAAGGCAAATGTTGTGGTGGCCTCTGTTGAAGACAAATTAGAAATTGTGGCTTCACGAAAAGTGAAACTAGTGGCAGATGTGCTTCTTGATGAAGCTGTTAAACTTTCATATGACCTAATCGTCTTGCCAGGTGGACTAGGTGGTGCCCAAGCATTTGCCAGCTCAGAAAAACTGGTGAATTTGCTAAAGAATCAGAGGGAGTCAAATAAACCATATGGAGCAATATGTGCATCCCCAGCTCTCGTCCTGGAGCCCCATGGCTTACTCAAGGGCAAGAAGGCCACAGCTTTTCCTGCACTATGTAGCAAGCTGTCCGATCAGAGTGAAATCGAGAACAGGGTTCTGGTTGATGGCAACCTCATTACCAGCAGAGGCCCCGGAACTTCCATGGAGTTTGCACTGGCAATCATTGAGAAGTTCTTTGGCCACGGTAAAGCACTAGAGCTTGCAAAGGTAATGCTTTTCTCAAGCCAGTAA

**2. UTR sequences of glyoxalase genes from grape**

>VvGLYI-like1

attataactaacggagccggcgccggccacttctctcccaaaaacgatatctatattaccaagagccaagaccaccaccacacacacccacccacctgcaaacacacgcagaggtagacaATGAGGACGCTTCCAATGGCAACAACGCCGTTTCAACACCTCTCTTCATCCCCACCTTTTCTCTCCTCTCTCAGATTCCCCACTTCTTCCTTCGCTTCTTCTCTCACTGGTTTCACCTCTTCTCGTCGACTCGCGCTTTTCCACCTTGGCACTGgtacgctccatttccagaaacctacattttattttcttctccttctttatttttctctacattaaatcatgaaatttcacgttttctctattcggttgttcattggtcgggcgcggattacaaaagcgttactttttttttttccttttaatttttattcgtgctctgttttctttgctgagaaaatgtggaaagagaaaagtaaacttattgaaaatttaatttaagttttcaaaactgaaaactcaactgagctgaggttttcacttatttttaggcttgcctgagcgaggaattgaaaataaaaataaaaataaaaataatctccttcacctttttccaacattttctcgggaaccaaacggagcattattgaactgaggccacgggatcagattgtgtagagactgggtcattgggaaatttgagctcctttgtgaaacagtgcgttttaatcagaatgatgccttgtacaaacaatgttaaggccaatggttaacttgtaaatagttggcttagttgttaggttgtttgaacaaatctagaagttaagaaagtagtttcacagtctgttataagcttctatttaaagaagctcttgtaatcaatttcagcagtgtgtgaataacaagtcctctgttttcttcaatctaaaatcccctattttcctttgttttcattaatcctaataaccaaaaaggaaaacggagaaagtctggatccgttttgagaagttacatgcacaacgatgtcgttttaatcatgtgggtggagcatggaccgagaaacctgtctgtcagtttacaaggaaagacaaaacgaagaaaatgataatgatggcttttacagtagaggcaaaagtatatgaattttttttcctgttcaaaaatggaagatttggtctttaaatgattttctctgcaaccaaaccttggaatttttgaatgatgcactaatggattggatttgcttggtttacccagCTATACCTCAATCAGAGTTGTTGGGTGGAAAAACATTGAAGTTATTTAGAATGGAAGGAAACATGCTAGAGGCTGGCACAGCTGGAAATATGGCACAAGCTGCCGTTTCAGAGGGAAATGTACTGGAGTGGGCCAAAACTGACAAGCGAAGAATGCTTCACGTTGTTTACCGTGTGGGAAACTTGGACAAGACTATGAAgtatgattatttagaataacattgatgaattcagcatttggtgatgtgtacaaagattattaattagcttgtgaggattgcagATTCTATACTGAGTGCTTGGGGATGAAACTATTGAGGAGATGTGATATACCAGAGGAGAGATATGCGAATGCTTTTCTTGGATATGGGCCCGAGGATTCCCATTTTGTTGTTGAACTTACTTACAgtaagccaatgcttaaacttccaatctcatttcctttttgaatgctaaaatgtgcatgatgcccttctatgaaacctacagtgatgttaatgatggcttctcgtttggggtttttatttatttgtttgtttaaatgatggcagATTATGGAGTTGACAAAATCGACATTGGAACAGGTTTTGGCCATTTTGGTATTGCAGTTGAAGATgtaagtaactactacctgaaatggatagactttctctttttgtcattacatcaagtcaagcaatagaacatgtcttaattttatgtcatagaaattaatttcctctctgtagccatttcttttttgactaaaagtaggaaacatgcttctccataatcttctatcttaatcttgagagttttgtgctgtaagaatcgcttttggcttccatatgcttcaaagttccttaggcacaaaatcttctctaggtgcacaatggcaaatctaaaaatttagcccatttcaatttggcagtaattagacatgtaaagcaactgtgatgggattagtcatgcataatatgggtatgaaatgacttttgacctttgtattcacatggggatctgatgatttgtaattttgtagtaactcattctggctcagatgtcttatatgtactctgccaacatcaagatgtcttataagcaacttttgaaattctctgtagGTCGCCAAAACAGTGGATCTGGTAAAGGCAAAGGGCGGAAAAGTTACCAGGGAGCCTGGTCCTGTTAAAGGTGGCAAAACAGTAATTGCATTCGTTGAAGATCCAGATGGTTATAAGTTTGAACTTTTGGAAAGAGAGCCTACACCAGAGCCCTTATGTCAAGTAATGCTTCGGGTGGGCGATCTTGATCGCTCCATAAAGTTTTATGAGAAGgtgagcagtgttccatgctgtatgtatgttgctttcttcatgaaaattttaacagtatttattggtagttctgctgtcatgatcatgaaatatctgggtttttaaatccttgtctttcctgaatctttagGCTTTTGGCATGGAGCTTCTCCGCAAAAGAGATAATCCTGAATACAAGgtaatttcacaaattctgctcattggctaaatggataaacttttgcaattctccagctatgcatatatacctttggaattaaaaaaaaaattagagttcttttcctatttgcttagttcagatgtataggtgtacaacacctacccaagaattatagaaagaaaaaggaaagaataaagtacagctataaaatctgatcctatcgatagagcctaaaaactggaagtcgatatcttaattgtttatgtgattaattccccaaaatctgctatatacaaatagctaaaaaaacaaaaaaaaggaaagaaattaaatgccaactggtacatactttcagttcatgtattccagcattttccatatgaatcaatttgtaggttaatgttatcacatgtggatgagtttgtaaggctgcataattgatgaatttgtgtattcctcatacacatcctgatcgaagtgcagccagcatttgatgatatctgccaaaacctggagtttgatttgtgaactcgcgtacaccagttgatttgaataggggtttgatttttctgatctcaaaatcttttgacatggctactctggtgatctgttgtgcaaaatgctagtttggatatgtggttgattatgttttttgtatatggtcatcctctgttgctccctttcatcgaatcagtaataggttgtgtcaggcaactgctgtgtaggactgtatctttacccaaacatgtgtcttatcgtgttgacattggttgtcctgtgtcttcaaagtggttgtatataacaggggtttggaaggagattttatatattgtaagcttgtgtcatcttacgacatgtatatgttagtgtatgaaaatttagaactgaatgctgttatctgacatgctgagacagactctcatgctgaaccccagtgctcagaattgcagggaaactaaattaatggctaagccatcaaattcatggtttacccatcatgagtccaccatatttactaggaaatattttattaacaaatctgctctttagactctcatcattgttaatgaaagaaattttaccttgtgaaagctttctttgcttttcatatttgttattttctttattgaagtgcatggaggaaaaaacacaattcttatgtgagcattgcttgatgttgtttgcatatctctactccattgatgttttattgtgtgacttgtgtctatgtaccagTATACAATTGCTATGATGGGCTATGGTCCTGAAGATAAGAGTGCAGTGCTGGAATTGACTTATAACTATGGGGTCCTGGAATATGACAAAGGAAATGGCTATGCACAGgtgaattctcaacctttcccaaaacgtgttttactgtatttcacaggcatatttaaatttcaggtcagtttgtgaatagtgttatcagaaacctttttggcgcttcaccgggcttactctactcaactggtttcaactaagacttgatatcaactacttgtggccagtactcaccaggcttgccctattttattaatttgcttttgacttattctttatattttaccagATTGCAATTGGCACAGATGATGTTTATAAGACTGCAGAAGCAATCAGATTGTGTGGGGGGAAGATTACTCGTGAACCTGGGCCCTTGCCAGTTATCAACACTAAGATCACTGCCTGCTTGGACCCTGATGGATGGAAATCGgtatggcttttgaccactaaaattttagatcatttaagatattgttcctaaatttgcatatttttagcctgatgtttgcttctctaaattggatgaattcatcggtttgctaatgatagttattttgaaagagtagggcatccaaaataacaatcaataagcaacaatatgaaggatgtacatatgcaggaactgcattcttgtgtatttatccttaatttgatttatcttttacctatcccctgacatgatcaccaccaccccatagaaaagaaaaaggtggaagaaaattcaccaactcgagctttccttggacttttacccattgtgttctgagtttaaacttgagtaaccaaatcttccaagagacctaggttttttgcccattctcatttttcgttatggtggattggaaccaagtcattctctaaccatttactcccataaagacgggatgcaggtcccacctattcactttaacaatttcccttatactctttaaagttttgaatgttaagggtgtgcttgggtcaaaagaacacattcttttggttgcctaccattaaaactgcaacaattccccccttatgtttactttcttttatgctaaatgatttattatattatatcaacatgtattcatatgcactgtggagtctgaaccttaaggcatttctaaaattgccagtagcattcttacttccattatttcatgtctttcttacacaaagttacttaaacttcaaaaatctcaaatttggaaattggtgcactacattttctgccaaactttgctgtcttggtttgtgtatccatgtttgatttcttgttggcagactcctatcagaaaaatgaaatatgtcctattcacaaaaaaaaaggtcccctagctaatagccctagattttaaaccttttcaataacataattatatgatatattgtgagggttttatgcatgaaaattatttctgcaatcttttctttgagaaaggtcgtgttttatttatgcagtgatagcattaatccacgtggtttgattccccaccaagtccttcttatgtttcccttgtgcagGTCTTTGTTGATAATGCTGATTTTCTCAAGGAATTAGAGTGAtcaaagcatcatcaaagctgccatgtgacttgactgaatccttgaaaggattcattacaattttgtttattaaaattatctccttgagccacttttcttacaaaagctcctgaaatgaaa

>VvGLYI-like2

atttctaattataatgataagccggggccgggagaagacagccactgcatctcccagttaaaatcgcgacaaggtgacctctctctctttctccttcttctccttctgtggccagtggccATGGCTTCCTGCTCTATTGCCACTTCTCTATCGCGCCTCTCTCTCCTCCGTCTAATCCCCAAACCCTCTTCTTCTTATTCTTCTTCAATTCCTCTTTTTCCTACCACTACTCGGAAGgttcttattgttggcagtggtggtttcaaaatttggcttatcgaattttttttttaattttttttttgtctccttgttcgtgttctttgcagGATCCGAGTAGGTTTCGGTTGTTTTCGGCGTCAATGGCTTCTGAACCCAAGGAATCTCCCTCTAACAATCCGGGCCTCCATTCCAGCCCTGATGAGGCCACTAAAGGATACTTCATGCAACAGACTgtaccctctctctctatttatattttctgtagttatgtagctgttgaagttgatttattttgttttcttttaatttaatttaatttttaatggcagATGTATCGGATTAAGGACCCTAAAGTCAGTCTCGATTTCTATTCTCGCGTATTGGGAATGTCgtaagtattttgatttttttttggaattcctagttaattgacgagtgtgatacagcagtttatgatattaatgctatccaaattactgaaaatatatggataataagtgtgtataataaatatacaaataaaatgaagtagaaaactgaaactcacaaataaggttgatcggggttagagtttgactctatgttcttaaaacggatttgtcccactcaagtgcttacggtttattgacaattgtctcccaggatacaacaatcactttcttatgataatagcatttaaaatcacaagaaacaacgaatcacttggtgatttgtactctcttgcataccaaatatttgatagaaaagagaaatgactagacttgaagttaaggaggacttaaatgacaagaaaagagaaagagtggagagaatggatgaatgaatggaatgctatgggggctccctatttatatatttgttggtgacttattagaagagacatgtctttaaaatactagacacatcatttgaaacaagtggtatcgattaggaaaagatgtgtcctaatttccattcacctatataaatattccaacaatcccccaaatgaatgaaaattgtcttaaaaaatgcaaatgacaagttaatcaatcaagagagtacaacttgaaaagtaattgcgtcaagataggtagcttgtgactttgaaccttccttagtaaaatattattggattagctaatcagtgaacgtgatgtcttaaactatttagccgttgatgtaaatcaagacaataatattcacacaattccctttcgaatcaccattgattttcatggttttgttcattttgaccctaaacagatcctggtttcatgagtgctataaaaaatttcagccttgaaaattctcatagaaatggccccatttcacactcacataggtgattcctattaagagtatcctgtcataccccacttgttttccaagctataagactcattaaaagcaaagcttaacctagttcatgctatagaaaacactatttcatcataggaataggtaagagattatctctttagtgtttatcttagtcattcataacttagttgtccttttgaacctagatattgggatctccagtcaactaggttgggttgccattatgaagattttttggtcataggctttagtcccattccccttgataagtagttaacttgatctcttggcaaacctttagtaagtggatccgcaaggttgtcctttgatttgatgcaatcaatcgaaatcactccatttgaaattaattgtcttgtattttttctctaaggactctcaaagttccttggcagttcttgttggactatacgcattgtataatgtgttgtccaaactattgaggatgtaattcttgtatagaaagtcagaatgattccaagctcccatagttgcttgcacttgtttatcggtttcaccctcctttaacatagggtcatcctcatataggacccgagcaagattcagagttgtaaggtagaacaacattttttgttgtcacctcttgagatctgtcccagtgaacttcttaggtttctcaccatttgcagcaataggaggtataatagaagatgtagaagccatcttcactaaaaaaaatcaattgtaataagattagaaataaaagaaaatttagcttaagcttgctatccaatttactaaaaatatatggataataagcgtgtatgataaatatacaaataaaatgaagtaagtagaaaattgaaactcataaataaggttgatcgaggttagagtttgactctatgtccttaagacagatttgtcccactcaagtgcttatggtttatcgacaatcgtctcctaggatacaacgatcactttcttatgataataacacttcaaatcacaagaaacaacgaatcacttggtgatttgtactttcttgtataccaaatatttggtagaaaagagaaatgactaagcttgaagttaaggaggacttgaatgacaataaaggagaaagagtggagagaatggatggatgaatgaatgaaatgctatggggggggtccttatttatagatttgttggtgacttattagaagaaatgtgtatttaaaatactagatacatcatttgaaataagtagtattgattaggaaaagatgtgtcctaatttccattcacctatactagacacatcctttgaaataagtagtatccattaggaaaagatgtgttctaatttccattcacctatattaaacacatcctttgaaataagtagtatccattaggaaaagatgtgtcctaattttcattcacctatatacatattttctttttgataagtaagaaaagtattgtattatgaagaggtgttaaaataacaacttaaaatgtacaaaacagaaagggaaatactcaccctctaacaagtgaaacaaaaaactgtccacaatgatatacaaaaaacaaacccttcccttaataggaacacgcccaatcaataaactccaaaaaggtcgatggaccatcttttataaacaatttagtctccaaccaaagtgagttaagaaaagaagttttcagcatttggatggatgacacctcatcttcaaaggcaattttgttccttgtctttcaaactgtccaaaaaatgcaaagaggacccgctctccaaactatcttatgccttcttcccacaaagaatccgttccaacccaatagggtttccttaactgaagatggaaacacccaagacactctaaaaagagtgaaaagcatttcccgtaatgcccttgtcttctcacaatgaataaggagatggtcaatggattcctcttccgcttgacaaaaataacatctatttgctaaagcccagtctctctttttaacttggtccaaggttaaaactttgcccaagatgcctcccaagcgaagatatttacttttggtcgtacacatgatatccaaatgttcttcatcggaaataaaattgaagagcccgactctagggctttgtaaagtgactttgtcgagaaaattccatcctttgtttccatccacttcatcctatcctcctcctccaagatcagattctgaccacacaagcgtaatagaaggccctccaactccccctccccccctattgtcccgttccattcatccttcacccaagtttccttaggattagctagggcaaataaggaagggaaagaagcacataaaggtgtatccccacaccaagtatccttccaaaagctcaccatttggccattccccaccacaaaggatagtctactactaacgatgtgcctaaccttcctaattgctttccacaaccctacaccataccctctcttacatcccgagacaaccaccctcctcattcctccccatcacctatataaatattccaacaattaaaagttgggttctgaggttagttgttggttgatgtggggtgattgtgttttgggggttgtgggatgaattgtattggcataattgtattattgaagagagcaaggttttgggagtccatttctttctgaataatgtcaaatagttgagtttcttgagtgtatgacgggtgagaatatcccttggtactaatcctctttctcatggaaaagtaccatataaaatgaggagagtatatccaggacacaaaggatgctttgtggctgtgttcccaccacaatttcaagattttatcttggtggaaagctcgaaacatttgtgcggaaaaaaaaattccagtagtttatgctgcagtaagctgcacttattttattgtattttattatcattgtgtatgattagcattctacacaaaatcacctatcaaatgatagaataatgtcatgcaagggtgattttgtatcttggaggcatgtccatttgtcacatgttttacttttatccttccttcccttggccctttctaaagagctgtgcatgtttggtttctattttcttaatgagtaccatggtttagacaccaaatacaggtagttgccacttagcaaggccttttagttattagcttatggtcatagtataatgatgctgcatgcatatttgtggtgtgctgaatttcttgtggcttatattggtattagttgaaatatattattgtaatttacatctattatatttatttaatctgcatattctccttatcttagccttgaaaaattccatatctggcaaatttgagttaatatagagttgtgggtcatactacagactttttttccttttgctattagattctttaaattgaaaattaatggttgtagcttttttttccatgttttcttgaatgttccagGCTACTTAAACGGTTGGATTTTCCAGAGATGAAGTTTAGCCTGTACTTTATGGGGTATGAGgtaaaacttcttatgccacttctacaagaatcttttattattattattattattaattccttgataattgttcatgttgacttatttgattttttttgtatgttgcaataatatatttatttgttatcagtgcatttcctgattttttttattcattagacaaatagtaaagataccattttttggtgtagGATACAGCATCAGCTCCCAGTAATGAAACTGAAAGAATCGTCTGGACCTTTAGTCAAAAGGCTACTATTGAGCTCACACAgtaacaattatcatttattttgatgtcctctctatctgcaaaacaatctttctggcttacccatccctagttttgctgacaacgtgtaatgttccaaaaaattgttacagCAACTGGGGTACTGAGAGTGATCCTGACTTCAAAGGTTACCACAATGGGAACTCAGAACCTCGTGGATTTGgtatgtatagctgaaatattgacttaagaatgtcttggaacttgacaatcatgtggtgatatatttgtctcttccttaacatatttgttgatagtcttctcgatgacaaatggtgagttttatttttatcctcttttgtggcatttatggaggtgtcaggacaattagggttgttgatttgagagtattgcataccatatgaatcattctgatctataatgtggtattgatgagaggggtgctgaaagccttagcttagatggtatttgggtgggaggagtggggctttggttggtttagatttagatcccgatgggaagttgtattgtgtaacattgtaccagtaagtggaaaaaggaacgaggcagaaacgcacctacttccttttaagtagcctcccaaaatagagaggaagctgcttccttcattccaagggactaaaaatttagtatcccagagtttctagtacatgtttgctgcaaaggtgatatggtggtgtcaactttctgcaaccatatttgttatttcgattgctgtaccaccaaacagagatgtacttgttcagagatgcaactacttggcggactaaatatatctccaagctactgggtgttactggagattgaatctgttatatgtataatctttcttcccttgacctttatataacatggtatccaaatcatcaagtgttgtctgataggaaaaaaattgaaatcgtgattttctgcattttggttcttctccaatatttcaaacactaacgtggctcattttgtttcagGACACATAGGCATCACAGTCGATGACACATACAAGGCATGCGAAAGATTTGAACGTCTAGGGGTTGAGTTTGTAAAAAAACCAGATGATGgtaactcacctttttttaattccatttttcattgagcactatcatgtctccttaatgaacatcggtttcttgggtttaaattagtaggtgggggtaactggagatagttcagttcccgaatctgctagtatgcctgtggatctgttctttagatggatcatcaatgtccctttcaggttatattttggtttgagatgtgggttgcatgcatgaccattaaaaaacctcaagtctgtgtatatgtgcatcgaaaagaatagatgcacaacaggaacgtgagaatccttgctgaaaggtaataacataaagaaaacaagaatgggaaacccaactataaaattgaacttcgaaaagattttgagttttgaactcctcttagttagcaggttggccaccatgcttgcaatcttaggtgttttaaattgcattactttagttaaggtttttaattctttgacatcatgagaaatctctctgccaccaataattaatgaatggcagatacctctactgacactatttagtctctcaggaatttggcatcaatttattcaaaggtggttgatgcaaggttgaaagctggttgtaagataaatcttaaaccctgatgggtcatactttgatccatgaagctgacaccaaagaagttggttaacaaagctttatggttttggttctggattctttgcatctaattcaaggttatgctgttgggatagcataacaaacttttgtttgggttcatgttccatgatgcatcctctatttgtctgaccacctgtttgggtgctatattgaaaaaaagagaacatcttcttcaactatgatttaaaaaaaaaaaaaaatagttgcttttatattatctgtcatgctggctgcagGTAAAATGAAAGGGTTAGCATTCATTAAGGATCCTGATGGCTATTGGATTGAAATCTTTGACCTTAGAAGAATTGGAACTGTGAGTACCACTGCTGCTTGAgaaaatgagacaagaatttcaggtaaacttttggcattcttttattcctattttttattaaatctagatttgcttatacatcaaaaagtatagttatttggctgaatagattttctgttg

>VvGLYI-like3

acaaactcgccaagaatttaataatccggtggctttggataggacaaaaaattgaaggcgaaaaatgtttacttatgtatttgtaaattggaaacagtgtgattgaagattcattggaatATGGCTGAGGCTGCACCTGTTGTTCCAAGTGATGAGTTGTTGGAATGGCCAAAGAAAGATAAGCGCCGGTTTCTTCATGTTGTGTATCGAGTTGGTGATCTTGATCGCACCATCAAgtatgcctttatagaatttgacaattgttttggtgtataaattataattgtactgtgattcttggagtttcctactttcacttccaatattgtcagGTTTTATACGGAGTGTTTCGGGATGAAGCTTTTGCGCAAAAGGGACATTCCAGAAGAGAAATACACCAATGCCTTTCTCGGGTTTGGCCCTGAAGAGACTAATTTTGTGGTGGAGTTAACATACAgtatgtttagacttttcatctttatacctgaaaatcctatttttgtaattttaatcctgtattcttgccagttatttgagaagtttaatccctaattacacatcagtggatagaattttgagagtgattttgattattattattattttttagtttgtagaacttttcaaaagtggtttttggaataatcaccaatcaagggatttttaaaagtttgtgaaacacctaatttatccgggaaaggatttccaagtactaagaagtatttataagtttctgggaatcacttcaaatggattcttaagacacctattttatatttatattcactgttctctctaggttctgttggaaatattgttctacctaggccactcatcaaagtacccgaagatgctaaaatgttaattcatctaaagatactacaatatcagttccggtggtgaaatgatatgcttaactctcaaatcacaaagatgttagggctctattagttgggaaacctttagaaaaatgttaggagactgaatcaatgtcctatcgaattctcaatcttcatcataaatgtgtgtggtgaaataaggaccattagatggaagtataaaggaactaaaattcatacgttacacgatgtgattgattctgctctttcatggataccgatttgatactgtagcggaaccaacttgatataggggacgattatataggcaaagggaatacgccgtgcctaaaatcatcccaagcgctgctgatgtaacagtaagttgaagagaataaaatcagacgtaagatttgtctactggccttctctagtttggcatattaaagctagtctaagactcgttccagccgtccccgaattcattaaaggaggtccccaccaccccacccccacctcattttttcccccctcctttttctttctggaaaggaaggaggatctggcaatatagtgatgggtttcaacttaacaaagccagcactctagagagagcttttactatctaatcaaggttttgagcggcaactatcgggttaactcaccaattcctccaaattgattctctattaactaataaatctctaccttggtcaatcacaattaatgtgttgtagtcctctttgccattatggtccttctcatgatctcttcttatcatgagtttgtgttgggtcctgtcctatgatgtcattaggcttcaatgaggaacaagcaattggaaccctatataaaacattttattgcaataattccatctacttatgttccccacttactaaccttatgttaatatgttgatagatgtgcctatgcacatacatgtactttgtacgtagatttttatgcattgtcaagatttgctatgctggtgtacctgtattttgggggtgggcttttaccatatctgatgaatttatttgctgcttgttcacagACTATGGAGTGGACAAGTATGATATTGGAACAGGTTTTGGGCATTTTGCAATTGCAACTCAAGATgtaagtgtcattctactgttttgataaccatatgacttgatttcagtggtgaacttggtttccttttcccatggaattttagGTTTATAAGATGGTTGAGGACATTCGGGCCAAGGGTGGGATTATCACAAGGGAACCTGGTCCAGTTAAAGGTGGAAAGAGTGTCATTGCCTTCGCGAAGGATCCTGATGGCTATATTTTTGAGCTCATCCAAAGAGGCCCAACGCCTGAACCACTCTGTCAAGTAATGCTTCGTGTAGGTGATCTGGAAAGGTCTATCAAGTTCTATGAAAAGgtaatttgatatccagttgatcttgtgtttgaattctgtttgtgcattgaattttctatccaatcttgtacattttaattttggataagcaaataaagattatggattgaaagcgccgagcaaaattggggtgcagctactaaagaatttctatcaaagtaaatggatactttactttcatatatcagaaaaaattgttataaaatgtttttcctacctctttcctgtgaagaaaattcctccaatagaagttgtgggtgtagattcatcctctgaagctttagcccctaatgattctttactgcagttacttcacatactagtcatcctgtgatgtgaaaaaggaaaaagcctactagatggaggaaagactcccattgttagcaggtgcctgtcctcatggtaacctttgaaatgaaaagagatgaagcattgaccatattatgctgcaagttgagaaaggcatgtgtttgcttgaatccattatggaaccacttatcagatgaagctgaagattaaagccacaccacccaccccccaaacacacatcgtaataatatatgtttgttcctgtcttaatctgaataaacacacacagccaccacccccacatataaatttgttcctatcctaatctgaataatttctctcatcaaatccatctagccatttgatcctttgatgtttctgcccgttctgtttttccccttatgcagGCCTTGGGGATGAAGATGGTAAAGAAGACTGACAGACCTGAATACAAGgtaaacaacgtttcttcatgtgttctcatgtaaaagtattcctttggaaacacagttatttttgaggtttgtgaaatcccttcacttgagctgcatatattttacagTACTCCATAGCCATGATGGGATATGCAGAGGAACATGAAACAACTGTTCTGGAGTTGACATACAATTATGGTGTCACTGAGTATACCAAAGGAAACGCATATGCACAGgttagttcagtacctctctttcggaaaccttgaacatatttatcttcatttcagtgcacttctgctttccccactgatgcatgttgtagttgtgtccattcccacttctgtttttccagtaatgataaatgttatacccattctagGTTGCTATCAGTACTGATGATGTATACAAGAGTGCTGAGGTTGTCAACTTGGTTACCAAAGAGCTGGGAGGAAAGATAACTAGGCAACCAGGACCAATTCCTGGACTCAACACCAAGATCACCTCTTTTCTGGATCCAGATGGCTGGAAGACAgtaagtttttctcttgtactagtaacttaacttatattcttgctttctgtatgtttttcaacaaactgctgcttttaaaagatgtaacacacatctatatagcaattgcctatcaacataaatactaaaataaagacttgggttgctgaaattggttcaagcattaaaagcccatttgtgagtgattttgaaagagtctaatgtttaacaaaaacgagatatttggcaagacaccattccaaccttgtaaagtttgattgcctgtaattcatatgtttaggtgggtttttctgcatcatctttggtctcgccttatgtttcttttccccttatacgtgtagGTTCTGGTTGATAATGAAGATTTTCTCAAAGAACTGCACAAGGAAGAGTAAatagtaaatacgaaagcttccgggtgatgtcccgagcttttagatccaccatctaatgaattacgatagtatctgcttttattagtatgtgtgttttggtatatatgctttaaaccctgt

>VvGLYI-like4

ccttgtttccaaacagcgccgatattatcagaacctcggacgctctcgccagtgtccagacgcacatacttgatactcgtaggaaaattcaagtgagagaaggcagaggaggtaggagagATGGTGAGGATAATCCCCATGGCGACGTCGTTTCGACCTTCTCTTTCCTCTTTCGGATTTTCTACTTCTTCTCGACTTGGTTTTCCGCTTTCCACATTCAATATTTCTCGAACGGTCACTTCGCTACACGTCGGCAGCGgtaaatcttcattccatgccctagaattttgtttttcttcttcttttattttatttttattttgtgttgctcaatcggcaatggacaattctggatttttgtcattttttgtttgtttttgtttttgtttttttttaaagattttattggatagaatttttatgttttgccttaatcaacctggaagaataaagaagataatttttggttacacgaggaatagaatcaggatcggtcaaaatgtttctcctgtggaaattgaattggtttattgtgtcttagactcctacacttttcatggaattctacgactgattgtatggcattggttgtagtaaagatttgcatttttttggatgttccaattatagatgatggatttgtattctggatcttcaaatgttatgtaaggaaaaatggagccattcaatttctgtagatgtttcattgtagacgatggattcttgctaatttgcctgagtgtggaagtcagtttgagcctcaaatctaataccccaagacccacttcgcttttgagcagtgggctagtatgactacatgagtttttttttttctttactatgtttggtttgtgcactgacttacaagacactttggatttgagttgggagttgttttgtaagggaaccaagtttgagcaactatcatctcaactaaactccggaaagaagtgttttgtgactgtaatatggcaccttaataaatttaccgaaagaatgtgatatcagtgtggtaaagaatttcaaagaattgaatcagtgttaggactgctatatcactgattcttgatgtgttttttcacaaaaatcctcactactcatgctaaagcatttgaaaaggcagtcttgttacacatgtgacacttttttttttactttatattttaatttatagggtattttttccatctatcttttcaaatgtaggagttcttgggtatctctataagtttaggtgttgtatattcaattagtgtactgagccaaaccaagtttgaatatcactatttcaagtcacttcccttacaaaaacaccaaaaacacacttctttcggttcatcttcaactgagattttcttgatcgtagaccaacctttgaagagcttctggatgtccttcttctaggatctgctggatgacatggcattcttgttttaatataggctgccgtactttgatggttcccaaggatccaatataaccacttaggtctatgttgcatgatattgttttatggaagttggcgagctgcatggttgttaactcgtaaaatgtatcttgtattgtttttttatttttctgtattgtgtatcgtaagtttttttatatagtaaaaaataattaaaaaaaaatgtatatgtgtgcatatctattgaaagtatgaagtatagagtaatatataaccaaattttaaggaaaatagtaggatttaaagagttaaactttatcatatcaaatgaaaacattgaatttaaagttttaacaagttcaaattaacaaaatatgactttaatatatagattcatcacataattcacaaaaattaacttcttcaatttttagctacaatttttagttccttattctaactagacatatgcccttattgtgttgtatatatatgcctttatgtgtcatctttaaagtttcagttgttcaagctcattatcattccttttccaccaaaaaagaaataaaaaaaaactaatatattaagtagaaatttttagtcgctaatcaccatcattttcattgtcaacagttaaaccattcaaatgaaaattcttcaatattcatcttaaaaataaactcacttgacttataatataatacttgacaacaagaatgtagcttttcatataagactattttacctatttctcatttttatgatcaatttttaaccttttagtatattttaaacaattaatataataaattatttaaataataataataatttgattttttttttaaatcatgaaaaatagttaaagatgtatgtatcattgcatcgtaatatcacgtatcgttttgttgtatcgtaatatcatgtattgtatcttgtatgtatcttattcttttaaaataaaatacagattgcgatacgtattgattttcataaaaaatgtatcgtatcattgtatcatatcatgtatcgtaagcttttaactactatggcgggctagtagaagtttggcctagtttgctttggtgctataggggagggattgacctttctaacgcatattcagtcgtaccagcatggccccactgatatgttttcaatcggaagattcggaacatcaagcagtgcaacccaattctacttgactaagccttgtgctagtccaaggcaaaaagcctttttttgataacaaggcagaaagccccaacccgacattaatgttattcattaatgtattaattattaattaatgaatccctagatttctttctttatcatcaattgaatcactgtttctaacccaaaattttattttattaccaatccaatcaccattcacatttttccttttttcttatcaatgaataaatctttttactttttatttttatttttttataggtaaattaggagaatatattaaaaaatagatctcttaacttatatgactaagacgtctcgaatttcttgaaaaagtgatttgattgataggatttggtatgatacttatgagatcgatgatatttatgagcagcctcctacataatttttgttgcatagcaatataattttcaacttatagatagggtgagcatcgcacgagcttgtaagttgacaaatgtggaggagtcttgtctatgaatcctgtcttgcttgcttttggcaaagcttctagtgaagcatctagcattagataattggaattaaggtatgataggaataccactagaccgacagagtgatagcaaagctaagtcatataaaagtgagcagccctaatagcgatgtaggagtttttgggtatctctataaacgcaagtgttatatattcaattagtgtactgagccaggctagtgtataagcttgtactcagccaaaaccaagcttgaagccttaatatcaatatttcagcttgagctaagttgaagctatctgagtaaatcaatagccagagcgttccacctctcagttttcgacttgcttaaccaacatgcattacattcacaaaacagaagtactattttagcattagtgtactcattttattctttttcccttatattgccattttcttattccttttaaataggttttctagtttaacctagttttcttttgggctttatttcataatgacaaatatcatgtttctgatttgcattgaaatatttatattgctgtttcagagatgatatgttgcattgtagctgatataaattacattctatcagtctgacagttgagttgtgtatctgttataattaattttaataattgattgaagaattttgaggactaagacttctattttgaggctcccatcctggaggttgtaattgttggaggttctaggtctcctaacatgcatttcatttgaaaatactgtttgatgcttgagatggtactgctatgaattcttcaaagacttgatgattttgttttgttatccagCTATTCCGCAGTCCCAAATTTTTGGTCTAAATGCTTCTAAATTGTTAAGAGGAGGAGAGGGCAATGCCATGGGATTTAATGCAACCGGAAATATTGCTCACGCAAGCACTAGTGCTGCCCAAGAAAATGTTCTAGAGTGGGTGAAGAAGGACAAGCGAAGAATGCTCCATGTTGTGTATCGTGTTGGGGATTTGGATAGGACCATAAAgtatatctattgaaacaccatgggctgctttgctgatcttgcgctttttttttttttcatttattctgaagacgaggatttgatcaaaatttatgtctggctattgcagATTCTACACAGAGTGCCTAGGGATGAAGCTTTTGCGAAGGCGAGACATACCAGAGGAAAGATACACAAATGCCTTTCTTGGATATGGGCCAGAAGATTCGCACTTTGTTATTGAACTCACATACAgtaaactaatcgtttcactttttttttattttgtcagcatgtcattgaatctagaacttcatataaatcttttggagattgctaaacatgcactgtactttgtatgttcatggacttattagtaaacctctctagactaaacggtgtctcatagatttttgtttaatcgtgctaccagATTATGGAGTTGACAAGTATGATATTGGAGCTGGTTTTGGCCATTTTGGTATTGCAGTTGAGGATgtaagttcaattccttaaattttgtgtatgttttcttaaccgtctctattcatacattaatattggtaagtgcttctttttctagtatctgtttttggcaacatatcttcaaggttgagtcacggaatatcaacttatttttcttcatcagcagattttcttttcaaaacactttctgcacctgaatgaattgtgctgcatgagattgcttttatttattattttaaataggagcacatctatgcataatgtgatcttgtaagctctcgcaataatgggctcctgaaatcatagacctataattttttgtaaaacctatactttggataatgtcctagatctattgttgtactattgtccttagtcgatttcattgattggttggggttggattgagggagggcgtattttgttttccttattctttttggcactctttggtggccgttgtatacattgtgtgtaatttggtgcaccccttttggcactttttattaatactattgccatttgcttatccaaaaataaataaaaataaattgttgcactattggtactcacagacatgtaatagggtcttgatttaatttctcttctgactgtagGTTACTAAGACTGTGGATCTCATAAAGGCTAAGGGTGGCAAAGTAACCCGGGAACCTGGTCCTGTTAAAGGTGGCAGTACAGTTATTGCTTTTATTGAAGATCCTGATGGCTATAAGTTTGAACTTTTGGAAAGGGGACCTACACCTGAGCCATTGTGTCAAGTTATGCTTCGAGTTGGTGATCTTGATCGTTCCATAAATTTTTACGAGAAGgtgagagttgccttaggcaagattgagaaagggcatacttttggcgataagctgttctattgctaatattttatttactttgctctgttctgcatttcataaagttttgattatgtacctctatttgttttttagGCTTTTGGCATGGAGCTTCTCCGTAAAAGAGATAATCCTGAGTACAAGgtatctcatctttcattagatcatcaattgccttgtactttcttatgtcgattttggatgcacttatcaatacccaaggttgtgagggttgggattctagattgttttgattggcctttgtggggccttgctgatgtgcccattctcttgggctgttactggtttttggaccaaactcctctggtttccagaataaggccttcaacaagccagtggatgtatacatgtcacaccaacttgttgatgcatgcagaaatgatttctataaatggtatattatctgtagacactgtttattatctggatgtatttacttttcttcgtagaaaagaaaatggcaactcttgttttccagtccaacttttttgcccggagctctgacttctttttttgtcaccactactggcttcttggacttctttttttcctttcattttattttattttatttttccttttttgggtgttctagctgtggtaatttctgaattccaaattattccttctcaatccttagattgttgtaccattgttcaattattccaaatgtgtttcatgttagcatttggtggttagtctgattctgcatctctattaggttgacactaacttgtggtccccaaccccctcttctctctctctctctctctctctctctctctctctctctctctcacagTATACAATAGCAATGATGGGCTATGGTCCTGAAGATAAAAATGCTGTTCTGGAGCTGACTTACAACTATGGGGTCTCTGAATATGACAAAGGAAATGGTTATGCTCAGgtggaaaccttccctctttttagttttccctgaaatccatcttgaaacatttttctgttgtgggcatgcatgacagcatgacattgattctggattggatgcacagtgctagacccattttctgggaaccaaatgaagcaactgatttctcgtgtcttgacagATTGCAATCGGCACAGATGATGTCTATAAAACTGCAGAAGCTATTAAACTCTCTGGTGGGAAGATTACCCGAGAACCTGGACCATTACCTGGCATTAACACCAAGATTACTGCATGCGTGGATCCTGATGGTTGGAAGTCGgtatggcttttaaaaagtttatgcttttgtgttttgtgtctgagaatttttacttttcacaagaaacttcatgaacacattgcactgttaatttgcttacaccacataagagttctagaaggtaagtcagatgttgaagctggagtttgtgtttttttacttaaatttaaatataacttaatttagcttaactttaaatacaacttaatagtattaaatattaagttgtttgtttttttattattttattaagtattgggtattaataggctgaaagttgtgctcaaattgtgttttggttgtttcaaaaagtcatttttcaaaaaaaccaaatattttaactttttctattcaatcaaaaaaatttaaaaagtaatgagtagaaaaaaaaaaattaaaaacaaataacctgaagtctgaaggtacattgcactcaacttttaagtaaaaaaaaaaaccaaacaccaccttagtatttttataatgggtttaggtgcttgaggcaactttgatgaacaggtaaaatgtcctccaaagatgcaccattgcatgtatgctagaaaaccgagttatgggatttaggtggagaaaaaccactgacaattgtaaaaactcaaatgtagttgtattggagacttggggatcaaattctgctttcacaagggtttgggaaattaggagggcttggtcctgtggaggaggactaccccttgatattcagtagggactattgtattattttttccgatggaccccagtaaaagaaaaatgatgagaaaagaaaataattcaaacttggatcgaaaatggattgtttgaccacctgcttgtgagtattgaagtttagatcagtactcaaaagattcagccaaacttcttgtatcgtgatgtactttaaattgatcactatgtctttttctaccctgtctttttcgcactccatggccatgataaggattgcttatgatttttctgtttgtcatattggaagtatagttgagcaatggttttttcttgaagtgttgagatgttggaagtgtgccatttagctatgctaaaacttctattacaacatgcattctgctatcaaatctttatttttaagtcttgtattatggagtccaattatctcgcgagaaatatcaaacccctggtgctatcttttcttttctaataaccatctttaaataattgattagtcatctttatataattgcttcggtttgatatcttgatatctctcctctcatattttctccagGTTTTTGTTGATAATATTGATTTCCTCAAGGAATTGGATTGAgtactttggaatgtcactataccaaagtctgcaaggacggatcgggagctagaatggtttgatcaactcaaaccttacttctggccaggatgattcttccaaagagtccatctcagagtg

>VvGLYII-like1

gaaaaaaaaaataaaaaaaaaaagggaaaaaaaaacccctcaaatatgtgtgcccattaatccttgtttatcaatttatcatactctctctctcctcttctaaatgtgaaatcaaaaagcATGCAAGTCATCTCCAAAGCACCCTCAGCCATGGCCTCCTTCTCTTGCTCCTCTAGGgtttgtgtcctttcccttccaatattctctctatcttgttactttgatctgattcttgatccaactgccttttctttttttggtcattttgagttgatatctgggattttttgttccattttaattttttttttaaagaaaaaaattggaagatctgatttttctcttgggttgggtgggggaggggggagatgttgagtaagtgttgttttggagggtgaattgtttattttgagtttgtatttgagatttttttttatttttttaatatttggtttatctgtgggtggaattgatgatctgggtttccgtttttgttttgttttgtttttaatttctgatcttatttaagtgctctatgaatgtggcaattttgagttggtatctgggactttgtttttattttttaatttggttgccttagtgggtgaaaatgatgatctgggactacttctctgttttttttttagagattttctgcgttcttttgatgagatgtgaatgtggcaattttgtgtgggtatagggattttttttttcctttttaaatttggttgtctttgtgggtggaattggtgatctgggtttccttttgctttgctttgctttggtttttgtttttaaaattttgatattatttagtgttcttctggtgacatgtcaatatgacaattttaagttgctccctgagattttttattttttttttaaaataatttggttgtttttgtgggtgggattggtgatctgggttccctttttattattattattattttctttaatttctgggcctaatttctgatgcaggctgtgtttgctttgtgattctgaatgagaaagggaggtatggtgactagagtatgactatgcaattcagggttttgatggaaatgtggcaaagtgttgcaagatgaatttctaattgttatgattgcttgtgtggtctgtgtttggctgtggagtgtgagacttgtgtttaggggacagatttggtatgtgggtatgattcaggatggggaagggaggtatgatgacttggtctaatcatgcaactcagggttatgatggaagtgtggcaaggtgttggaagatgggaggggttggtttcaaattgttacaattgcacagtataagtttggactctttgggaatgggagattatccttggcagctttcattgatatgttaggttgcatgcccctaaggcactttttgatataatttcatatttacctcagaaaaaaaaaaaaaaaaattctctttggcatattttgaggatgaagtatggatttggatgttcactttttttaaaaaaaatatatatatttttcaaaacttggttttaaatcggcatcatggttttaatgtataactagtagaatttacattggcgtatttgtaattatgcattctaaaccccgatttaaagtgaaagtgggttccaaattgatgtgatttgtatagtttagtaaaaatccattttttatatggcttggaaacttttctcttattttcttgcattttggagtctcttgggttcattgggagtgaggaggacttgaaggggttatcctttgattgtggaaagtgacttctcgttgcattctgtggtgcatttgaaaggagggaataagtgagctcttgatataataaagctctctgttgattaacttaaaaaaagtgtttcacatgtccttatttttgtgggttagggggctcagagactctcaaagggatgactgtttttgaactttttgagtactctttaactttttaatacttgtcttttattgtgcacctcaaataaacttgtacttggggtctgctccttttggtccaatttatttgatttggctatctcgagaggaaaaaaagttaatcacatccttacatcttttcttgatgaaacaaaaacctaagagggcatggtttatcatttcaacatgcatgattctggtattaaaatggttatatagtgaagctcatctgcatatcttccctttttcttttcttttttctttttggttttgattcttcttctggtgaataggaacttaatgttagtgctttctattttttccatactagtcttaaatgtcaaatctttctttggatgcctaaagcctattgttttcatccaaaccctttgttttgaaggtatttgcttcattttttattttttgtttattttttgatgggcaacatattcacttcatattgaaatattcaaaaacctttattttatatttcagGGGGGTAATGGGCTTGGTGTATGGCCAGGCATGAGGCAACTGTGCCTTAGAAAAAGTCTTTTGTATGGACTCATGCGCCTTTTATCAACACCATTCAAAACTTTGCGCACAGCTGGTCGCACACTCAAAGTTGCACAATTTTGCAGTGTTTCCAATACGTCTTCCACATTGCAAATTGAATTGgtgagcatctctctctctctctctctccatacacacacacacacacacacacacacacacgtgtatgtgggtagataatttttatttacgcagttgatgctttggttgctactcttcaatctcgtactccacaaggttaggttttttggtgtacaccatatttctatgtctatgcattgttgttccctcgacattccttatactgcttttccaatcttcaaaatgcttcattgcctgaaaaagtgacttgtttttccatgtgaatctgattgcccttacttaatgtattatcctgtataaaggatagttcaaagatccttagcttacataaaaaggataactgggatttgtctgtcattccccaggcccgctttcttactctcattctcagcaatgtgctaaggaagaactgacaaggcatacaaagagaatgatatgtttttggtaattttaatttgaaagccttctgaaaacaagctttgatagttgaaagagactaaaagcaaagggaaagatgttgattctacagaatgatctgtacagatagtgacaaggttacttttgaattaaagaaacagtaaaggtcagtttgctttcgaattagggatagaagatccaggcccagctgtcaatatagactttttggatggatcaatttatgtaagaaccaaggagctgtgttgatgtgcaattcattttagttgaatacaccctctacggcttggttcctataaaattgcttaaccaaagctatggagctgttgacttccatgctgttttctattgtcactctatgatgggatatggttcacgtgggtactacccacagtggaatcttaaagaaagacctccaacttgtgaagaacccttgaaaggtgagacgatggtaaaggacagtatgtaggcaatttcccttctttcctcatgtgagtgatgtgtcttaaatctggtccttttgtaaaatcaaaatctggtgtgttgcagtgcaaggcactttctcctggcctttcgaagtcttaaactggatatttctcttccttcttctacaatcaacatcacttgcacacacaggtgtctcattagatgagtgctgtctggaatagtgcacttgaatttgtatggttctgcatatactaacaagtactgcatccaagcttgtaacttaattgactcacttggtaacttaattgactcgcttggtaacagttctttttgtgcttcctttcacacataaatctttgtcaactttttccgtcttcagGTACCATGTCTGAAGGACAACTATGCATATCTTTTACATGACGTCGATACAGGCACAGTTGGAGTTGTTGATCCTTCTGAAGCCGTACCTGTTATAGATGCCCTGAGTAGGAAAAACCGAAACCTAACCTATATCCTGAACACTCATCACCATCATGATCACACTGGTGGGAATGCTGAGTTAAAAGCAAGGTACGGTGCAAAGgtatgattatcatcttctggtaaagttgataagccttttcttttttccttttgctttttccccctctttttattcacctttagggaaaatgctcaagtgacaagggaacattgacccataaaagtggtttctctctcctgccttttacgctttcccaacattagtcatccaacagggatcccccttccaaaagttgatgtttgcaaaccatgtgagatgcagatatggaaggaggttgtcttctttcagtccatccaaaggaaactatttcctttgggaatgacattcttcttaagttcttccaaaaaatatcatattatcctgttgccttcttgatggaatagaaccattgcggtttactgaatgagcaatgaagaacaggggaaaattgtgaaagtgctactttttctaaggttacaacaaaggttggaaagcaggtttcaactgcatagtttaattccaaagaacaaatggaccaattttaatcctgccattgtcttcatatttttagatttggtaccatgctatgactaacatgaaatccacttggtgccccaagatgacttgatgaacctacctcatgaggacaccccaaattagcctacaccatcttgacctaaatttttaatagcctataaaaaagaatggatacatttgtttgaatacaaagggagtttggtcatcctttgttaaaaattgctattgtttgtgaagcagttcattttgcccatgcttatgattttttttgaaattgtaaacttatgcttttttgtgaagttttaccctttttgtgggtttcttgtagGTAATTGGTTCTGGAATAGACAAGGATCGAATTCCTGGAATTGATATAGTTTTGAAAGATGGAGACAAGTGGATGTTTGCAGGCCATGAGGTGGTTGTAATTGAAACTCCTGGTCATACCCGAGgtcatatttcatcactaatcatattagaaaatatctggatttcgtacttttgaaacatgtgatcatttcaaaatcaactgatgaaaacagtacatttctttctaaactggcttctatagttactcagattttctgtggggagggggaaacatggagagaaaagtgcatttggttaaatgggaggtggtttgtacagacaaagagaaaggtgggctaggccttagaaagctagctttgttgagcaaagctttacttggcaagtggatatggaggtatgcatgtgaaaaaaatattttgtggagacaagtgattaaggtgaagtatgggcaggagggtcttgattggaggcccaaaaagggaaatggggcggttggtgtaggggtttggaaggagatttggaaagaatcagattggtgttgggataacataacattccgtgtaggaaagggcaacttgatttgtttttggacagatgtgtggtgctcagaatctccattggctcaatgtttccctcatctctttggtatggctgcgcatcagagtttgacggttaaggaaatgtgggatcaaaattcgggtcaaggaaactggaacctacacttcttgagggacttcaatgattgggagattgagctggttggggagttccttcacattttgaggggttttaaaccttctttggaggatgattcagttttatggagaaaaggaagaagtggtcagtttagggttaaggaagcttacagtttgttgacgaattctgaggttatgggatttccccataaaagcatttgggtggcaagagtgccaactaaagttgctttttttgcgtgggaggcgtcatgggggaaggtgctaactttggatagtcttcaaagaagaggatttcaacttccaaatcggtgtttcttgtgtgaatgtgaagaagagagtgtaaatcatatccttatatattgtacagtggttagagctttatgggatattgtctttggtttagttgatgtaaaatgggtttttccgggaactgtaaaggaggtcttagctagttggaggggttcgtttgtgggaaagagaaggaaaaagatatgggatgccattccgttgtgcattttttggacggtatggaaggagaggaatagattagcttttaggggggggggggggggnnnnnnnnnnnnnnnnnnnnnnnnnnnnnnnnnnnnnnnnnnnnnnnnnnnnnnnnnnnnnnnnnnnnnnnnnnnnnnnnnnnnnngagaatacagtttgcactttccactcatggttcggcatctttgctcgtgaaattaaattaatacatgacatagttgatcactgctagagataagggaagtgtttcttgtgtatcataataaagttacagtgccttatagtactttctatatatttttcttacctattaaaaataaattgggatgtttctaccatgtaagcatgatgcttttgtcattttctttttaatcttttaagtgcaaatattgttaatgacaggccttcaatcaattattgttagtgacaggtcttctatcaatcttactgtgcaataaggctaatttgcccagctctagaaaatgttgttcatagatttttttttttttttttttatctaataatataggttccatgaataggaggcagatgtaatgtttagacgatgggtgcagtcagtgtgtggttgggggggggggggggtttgaatgtccaaaagttaaagaattcttttgtttgtaacttgtggagttgggccaaattatatgtaggtgaggaggcgttctcccttataggctttttagagtggatagcctccacttaatggaggtgatcctttgttctgttttttgaggcctatgctgttttgtatacttcctgtatgttgtgcggcgttttgcctttttttaatgcatatcttttacttatcaaaaaaaaaaaaaaatagttactcagattttcactttcttctaaatgggcattgttgcaattcagttgatgttcagaatgatggtttggtttaacatgctcagcacataattatattctctaatctcgtatttttcactgtgatcttgagtatgtttttctttaaatgacttaaacatgtgatgtcatgctgtgttgatttggaatcattggaaaccatatgactaaagcatataattaaatctgaaatataggactgagaaaatgaggttttaatttgccgttgttatttatttatttttactatagttcttgtcctgtgattattaaacaaagaggtgtttcaaaccttgttgaattattggtagttgtgcaatatggtccctgactctatgcttagatatataggtcttgcaaagtagtagaacttttatattgtttgttgagaatctttatactatcaggttagagagaatttctcctaaaatgaacaaatgcaaacaaaagaaaaaaaaattacaaaagtcaaatcaatacaacagtatcaaaggagtataggactaaggtggcacaaagtctgcaacaaaatcctcaagagaagtaacagccactttggctagttcactggactcctgattgaccatcctaggaaatccaaaacattgcattgagttggtaatttgaaatgtcaagggccttttttttttatcattctggaataaatccatggatctttccttcttttttcaacagagtcaatcatcaagtgactatagccttgtagttgtaagtctgatgaacctcttatgttggcttcttcttggagggtctctcaaattttctttttagtccatgtgcatcttttagatgagactaatccagtttcttcacattgaggctctttatagggttttataagccttagaaacaagtaaaagccttctaaaattcatcctacgtaacatggtaacatattaaaattcaagttcgatttggaaatcattcaaggtcacaagagaaaaggctgtatcaaataaaagactggtcaattggcaggatccacaaacttgttgatggattcaccactttgtcctcaaattccattatatttggcttctagattcacatagttccccctttttcttttctttattaataatttaacctaatttaattttgtgtattgattttaattgcaagcagGCCACATTAGCTTCTACTTTCCGGGATCAGGGGCAATTTTTACAGGGGACACTTTGTTCAGTCTATCATGTGGCAAACTTTTCGAAGGAACTCCTGAACAGgtaaattttgactggagttacatttcttgttctttttctgtccagggctttaaaccatctaaattatacactcaaacatgccatgtatccttttttggaatatattcaacatttgtagaagggaattacattttcaaggacaaacaaaagatccaaaaggctgctccatgaaaggtcaaaaccctcttgaagaatcttcattgaaaggggattcatgtgccttagctcatatagggctgccaaccatttgtcctgttagattgtacatatgcatgtaagcatgtgcatgctgcacatgttagatatcatgaatttcttttactaatatatatatatatatatatatatatctttattatcaattattttactgctgcacatgcttgcaggactgccacacacatgcatggatggatatgcggttgtactcaaattgttaatttgactagatgccatgttagatatcatgaatttcttttactaatatatatatctctagtatcaattattttacctaaaaaatatatttctttaattactgtctgcagctatgaggtttatttaggctcgacatatgctgggagttctctgtgaaagatgatgatttgaaaattttccactcaattttctgctggaacgagaatgatgagaatattttttaactgcaatcttaaggtattagaaaaataacattttcataaaaaaatttcctagggattgtgtggggggggaggagattttccaagttttggagtttctaagctttgtatatcaatatggatatatctttccctaaaagtggcttttagttccaaaggaaacccggaagtttcacaaatttattggagatccaaaactggttgtcacttaggtaaatatggaagagccttcccattttcatatgaagaaaatgggccatggttgtgaattgctaaaatctctctattgacaatatggagcatgaagatcggttatttgtaatctgccattaactcttttgtgattgacatgattcacatattatagcactgacctcctctgtgttccagATGCATTCTTCCCTTAGTAAGATTATGTCTTTGCCAGACGAAACAAATATCTATTGTGGTCATGAATATACATTGgtaagtcattttcttcttcagcttttgctcaggtttgttaagctcacaacttttgaagaagcttaactatgtgaaacccattttgatagAGCAATTCAAAGTTTGCATTATCTATCGAACCCAAGAATGAGGTCCTCCAGTCCTATGCCACCCATGTTGCCCATCTCCGTAGTAAGGGCTTGCCAACGgtaaatttctcttgttttccttccttcagtaacaaatttctaaaacttatgtggggaactgaattttccagtcttttgcttagatgaatgcttgagtctgcatttgttgtggaagcctctagagcgcctcaatgagtatactaaattcttaggacatcaggaaaaggttgataagcaatctcttaaactatgagcatatgcaagccatatggcctttttaattgggttaattagttatgggtttactcattgtggaggctttatggtctttctcacttgatgtgcattactaattttctcataaacatagcatttacagttctcaaagcattttcagtttcttaatcctcactgttgtacacgaacccgttaggtgatgtatgtgctgcatgccctagtgtattttgatttgctcaatctctttggtgatacatcactgaagtggaactgttccaacagtccctagggtataactatgaacaattggatgagggcagggctcagcttgattgtaggttgttttagtgggaccaactggattttacaggcaaggatgtagtgggctgaggtgccctgctaagccggactctctcatttcaaacacaccagtccatgcaaatccaagacaaaacccgaaaaatgggccaatgctcatactagtagtgtagcttcttattgagttggacaagcagtggacaagcttctccaaggggctgccctgggtttcaaaacctctaggatgatctcaggctttttccagaaaagccaagcttgcaagctggtttgctgttgtgatccttcccctccacactgcatttttgcatttttctggtttttctcaggcaacccatgtttctttcaccttcacagaatgcttattgctgcatttaccttaaccaaagctggccatgttcttgatgtctcagATCCCAACTACACTGAAGATGGAAAAGATGTGCAACCCATTCCTTCGCACTTCAAGCCCAGAAATACGGAAATCGTTAAAGATTACAGCCACAGCAGATGACTCAGAAGCGCTGGGGATCATCCGTGAAGCAAAGGACAACTTTTAAgatcatcatcatcataccttgttcttgattttgtataggagggggtgatctctttcgcatgtgaaataaagaccaccaatattttgatgatatcaattttttgttttttcctctgttttg

>VvGLYII-like2

ggcccaactaggattcagcttgctgggaggacacgtctcgacggcggtcgttgtgtggtggatggaaatggaacgagggtgatcgatcagtcggacaactgtaaagggtttgctggcgatATGAAGATCGTCCCAGTTCCTTGCTTGGAAGACAATTACTCTTACCTgtacggatttaatttctcgggccataagttaaagacaggtgaattagggttagtgtttgtggctaaatgtgggattgactgatttacagGATCATCGATGAGAGTTCTAAAGAAGCAGCAGTTGTTGATCCAGTAGAGCCCCAGAAGGTTCTTCAAGCTGCTTATGAATATGGCGTCCATCTCAAGCTCGTCCTCACCACTCACCATCACTGgttcttgaatttcttcctccattttttttttggggggaggttggatttttttttctttgttttaattgctgatatgtggtagGGATCACGCCGGCGGAAACGAAAAGATAAAGCAGCTGGTGCCCGGAATCGAAGTGTACGGTGGGTCTGTCGACAACGTCAAGGGTTGCACCCATCCACTCCAAAATGGTGATAAGCTGTCGCTTGGCTCTGATCTCGCCGTTTTGGCTCTTCACACTCCTTGgtaattttctcttgcttccccatttggattttctgattctcaacgtttagcactactttttctaatttccctttggaaaaatgaacttttctttgtgttgtttcaattttttccttcccaatgtctctatgatacactctgtgacctgttttataacattttgcaaagtcagggccttaatcctctgtatctttggagaatagcattactttttgtttggttttttgatggcctaaattctcatattcatgtgtctcctgggcttctgcaaatgcaaaccatagatactttgtgcttgctttaatatgaaatttaccttgggtagagaactgattcctgttatttgcatatgcatctccagtaaacttatttctgttccattgtttgaactgaattacttcggtctcacttttttctctctggcttgctttcatcagTCATACAAGGGGTCACATAAGTTACTATGTGACAGGCAAAGAGGAAGATGTCCCGGCTGTTTTCACTGGAGACACATTGgtaagggagaaaaagatctcatagtgatttccgcagtcaattttcatcaagataaaccagagaactgagctatgtaagatatacatggtctcggaagggattgcattttcctctttgggagagtgaatcattatgatagaactctggaaaaaattgctttaggtgggatggtctgagttagtcatccttagaacatcttggtaccatccttgcactgcattctgagtgcaacacctttggctatgaatattattaaaccacataagtttaacatacattggactatattattttttaagaaggcttttagttggtgaatgactggatccctttactttgtaataaaatcttgttcttttttgacttgctagttacgatgttaaacagttaatagtggttacttcatgcatgtgcagTTTGTTGCTGGTTGTGGGAAATTTTTTGAAGGCACAGCAGAACAGATGTATCAATCGCTATGTGTAACATTGGCCTCATTGCCAAAGCCAACTAGAGTATACTGTGGCCATGAGgtaaaatgattcttaatacattcattttggagattcctggaattcgacaataccctctagttttctcattattgttgtttgacgaagaaaagtattgtggcattacttgtctgagattattccatggaagatgtctctagtaacaggtattcatgatgttatttatttttccatgagtcattggttcacgtagtggaattttcccagttaacctagtactgttgttcatcttttcctcacagtggtaaaatgatgcaagtaatataatttcatttttaatcaaatcacttaatatgtaaacgagattgtggtgtacagcaagagccttgaggaacatgtgctatacacctcaggcttatgctagacaacttgagggctttagctcaactatacttgaagagggtcgtaaatagagctcaggttcgaattccacagagaatatggatgggactgaactcgtaccaacccctcttcataagctctgactgcacttccactgctttagattgagtcaaattttggggctatgactgtttgcattggaaatcaaaaaattcctctaccctcactatcaacttaaacacctcaccggatattgatatagtgaggtaagaggctgggagactaagggtggtagttggagcaagcgtccctcgcctcgataagggcaaaaggtacttatagtgaacagtttggaatgaactgatgctggttatttatttttccatcagtcactggtttacatcgtggaattttcccagttaactaagtactgttattcagtttttcctcccagtagtagtaaaatgaatgcaagtaatataatttcatctttaatcaaatcactcgaagtatgtggagatgggaaaaatcttaacttgatcgcacatgtgggattgacagattttgttggcctgtattacttggatttgttttggtgccaacagataaaatgactctgtaaaccctgtgaattgaccatagcaaatacattggttttaatagtatgccactcagggttacaattgggactttttttctagcacaaattcctgtgtaatacgagctcttctttgccccaaaatcaaatcaagtggatctttttgcattgtgacacttgatattaaattggaaagtagaacttctgaaatgggaagacattttaacttcagtcaccccaatctaatttcaatagtcatcataagagacttgcagttctcctgttatatctttttccttcattttcaaggaatgtaatgcttttctttatgtttctccagTACACAGTTAAGAACTTGCAGTTTGCTCTGACTGTTGAACCAGATAATGTGAGGGTGGGGCAAAAGCTGTCATGGGCACAACATCAACGACAAGCTGGCCTCCCCACCATTCCCTCAACAATTGATGAAGAGATGGAGACTAACCCATTCATGCGGGTTGACCTACCAGAGCTTCAGgtaggcttttttgtccctttgaagcaacctttgtatggtgtttgaagagaacataatggggatgaaatgcatcaagtctgcctaggcattcatgaaatgtggactcagaattgcaatggattccttatacttgtgcataataatgtagccaagtgtagtgtatcatgacaatatgtctaatatgtcatatggctttcttgactagaactttgcctcttttacagGAGAGGGTTGGTTGCCAGTCGGCCATAGATGCTCTACAAGAGATAAGGCGGCAGAAGGACAACTGGAGAGGCTGAactaaggacctgcttggcgtgttattttgtttcttcttgaatatacatgaacggggtgtgtgataatgtgaaagtagcattacattgctgctttggcttgggttgtgcaattcgtgtttt

>VvGLYIII-like1

ggggatattcaatttttttttttgtttattagaaaaaaaatggggctcaaaccggagaagcttcaaaaaatctggatatagtgagaaaccagagctgaaatttgcagttgatgaaacgcaATGAAGTGTCTGAGTCTATCGCCGCTTCTCTCACCACCTTCTTTGAGCTTCTCTTCTTCCATCAAGACCCCATTTCTGGTGGCTCTCACCTCCACGCCCTCAAAAACACATACTCCCAAACGCTCCTCGAAATCCGCTAAAACTCTCTTTCCAACCACCACCACCTCTCTTCCTCCCAAAAAGgttgtcgcactctctatttttggtttcggacaaaacccagaagggaaaacgtagccgaacgaaatttttttggccctttttgcattttcccgggaatcaaacagttttttgaggggataaagttttagggttgtgaaggtcttaattttgtgagtttgtgttatggacgcagGTTCTGGTGCCTATTGGATATGGAACGGAGGAAATGGAAGCGGTTATTCTAGTCGACGTTCTGCGGCGAGCTGGTGCGAACGTGGTTGTGGCGTCGGTGGAACCCCAGCTCGAGATTGAGGCTTCTAGCGGCACCAGACTGGTTGCTGATACCTCCATCTCAACATGCTCTGATGAAATTTTTGATCTCATAGCTTTGCCGgttagttttcaagaacgtagatttcattattgctgattttatacatcgtatgattcatgaattggagaatggggttgttgtgatagtggttgcagcaatgctgcctgataatggtaactagaatttattagattgacaattggagattttggcaaagaataaggttagagaagttcctatcaactgtttgaagcaaagagttttaggggcttgtggtgttagatgaggatcctgcattaattggtagggccattgaagagaagcaactagtactcaaaattggaggaaaaattctagttctttaggccatgtttggttatgcaaatttgaggtaaaatgcaagggaaaaaaataaagaggaaaatagaaggaaagaaaaagtaaagaaaaattttaaaaacagatttagaatcaataaattatttttatatggtatgtcaaactcattaacatatttaagtttttttacataaaaattaaataatttgaaaatgcataagtttccaagtagttttaatcatatttgcttcttttccgtatttttcatagtaaaacctatcatgagaaaatggttttccttaacattttttttcctttcattagtgctttcctagaaccagacatagctttagatttcttgttctttttatgtttgcatttcccaattgatgaaatcaattgttgttatttgttttcccttttgcgatgtttattcttctgatctggtttgatattttataaatggcttatatcatgtgcattgtttaagGGAGGAATGCCGGGCTCTGCACGATTGAGGGATAGTGAAATTCTCCGGAAAATTACTAGCAAACACGCTGAGGAAAAAAGGCTGTATGGTGCTATATGTGCTGCTCCAGCCATTACACTTCAACCATGGGGCCTTCTGAGAAGAAAACAGgtaagttaggtgctttgtcttttgatcaagttctaatgaatttgaatgtaggaatgtatttggttggctgatttggatgtagtttcttgtgcaagATGACTTGTCACCCAGCATTCATGGACAAGCTTCCAACCTTCAGGGCTGTTAAATCAAATCTACAAGTTTCTGGGGAGCTCACAACAAGCAGAGGCCCCGGAACTGCTTTTGAGTTTGCTTTAGCATTAGTTGATCAACTTTTTGGAGAGTCTGTAGCCAAGGAGGTTGGAGAATTGTTGgtgagacctactactgtactgtgtaactcattgtactattgttttgtgccattgctatcaatattctctatttaaatattgtgtgtgaagtgtcaactattttctgatcaacagatggactttgatctctctcttttccccttatgtgtcttttccttttctttaaatgtattatgtggctaatcagcttgagctaactctattttgtgtctttcatgtcagCTGATGCGTACTGCTGAGGACAATCATAAAAAGGAAGAGTTTAATGAAGTTGAATGGTCAGTTGATCATAGTCCTCATgtaagtattccttttattctcaactattttgtcaagggttcagtggatatgattgttggttttggggtattttttgaggttacttgcttttgtggtttcatgtcttggatccgacttttgtggcttgaactttggttattaacgtgactaaattggattttgtattagttactaagtaacattaagttcacatattctagcatttgttgtagtggtaggtggtcatcaaggatttacagtacaaagttcctttagatccattggaatatattgatccagataacttggcatatgatatgttttgtccgttagaacatgtttgactaaacatatgatcactattgctcaaccttctaattgaggtccaggattttatctgtttttgtttccagtgttaccctgttccacaccaaaagtgaaataacagacaccagattgcggtttgttcatcccctttcatagttttcactagaaatcttgaagcaagtatgtgcttacactatattattggaagaagttgcttctacaatgttgcatctatagttgaaccagactggcattttcagggttagggttaatgtcttaagagctttcaccatattaggagtgttaatctttctgcttcttgtataacatgtgatgggaatcatgggatgtttaacatcaaagtggctgttttcatttgtctacatttgtagccattcaatagtcttgtttgcagagattcttcttttatggtgaactttcttatcagtagagggtgtgaatctgaaggctataatgatttccagcattgaatttgttttcccaattatcatgatctgtgtgcaaatattgtagtgtgttcagttctatttgcacatttttccaaattgttcatccaaatgttctgtttatgttttgctgattcccccaattcctgtaaactgaaacacttcccataatgtttgtttagattcatcgggctccaatggtcacagtgctttttcttttcttctgtctaattttagccctctaaactcgatattttctcatcattcaagtatgtcacagGTTCTTGTCCCAGTTGCAAATGGTTCTGAAGAAATTGAAGTGGTTACAGTTGTAGATATTCTACGGCGAGCAAAGGTGGATGTTGTGGTTGCTTCAGTTGAGAAATCTTTGCAGATTTTAGCATCTCGAGGCATTAAACTTATAGCTGACAAGTCAATTGATAATGCTGCTGAATCAATTTATGACTTGATCATTCTTCCGgtgagacaaacagaaagccttgaaaatctaattgttctctttgatttctagttttctcagtagaattatcagaagtcaaagattcttcgtaagattttgattttgtcctttggcatttcatggcactgctggttttggttatatatatatgtgtgtgtgtgtgtgttgcttggagtcagaatgaaatctcaaaattctcttaatatttttctatattgcttgggaccattgggtcagtaaggacatgctgctaatttccagttatgctgaatatctggtaaatgcagttgtctgctactgattttgttgtagattctaggaataatcacttcttggttaaatacctgtttactctctcagatattgatagtctggtacatttccaatcctttcaacttcattcacatgtgcattctagttattttgtattttggtacacttatccattatttggaatgatgtgtctggacacacaatttgttttttcaagaatgcattgtgttccccactgttgtaattcaaaatctggacataataaaccttaaagtgactgtcttttctactcaatccacattgctgacaggttggttaattccaaatttcagGGGGGGATTGCTGGTGCTGAGCGGCTGCACAAATCCAAGGTTCTGAAGAAGATGCTCAAAGAACAAGGATCAGCAGGAAGAATATATGGAGCAATCTGCTCCTCGCCAACAGTCTTACATAGACAAGGGTTACTCAAGgtaagtcgagacaagttgatgtatgcatgcttctaaacagtggtattatacacttatgcttccttggccaaaaacagGGTAAAAGAGCCACCGCTCATCCCTCTGTAGCTAGCAAGCTCACCAATGAAGTAGTGGAGGGAGCTAGGGTAGTTATTGACGGCAAGCTGATTACAAGCAGGGGGCTTGCAACTGCAATAGAATTTGCATTGGCAATTGTTAGCAAGCTTTTTAGTCATGCAAGAGCAAGGAGTGTAGCTGAAGGTCTTGTTTTTGAGTACCCTAAGAGCTAGagagacagtgtctggcacttatggttggagttaccctgcaccaattcaagtcagtggttgaaatgagtcaatttcttgccagctgcttgggcttgaaatgagaattgggtttttgtaatc

>VvGLYIII-like2

ctcacagcactcagggcatcttctctctcagtaacgctcctcataagccataccactccaaaactcctccgaagcccacgcgcccacagtcactctcttcctcactctgtttctcacaagATGGCCAAGAGCGTTCTCATCCTCTGCGGCGATTACATGGAAGACTATGAGgtctttgtccactctaggcggcatgaacagttttggggttgattgttatctcactatttcatgtcgttgcagGTGATGGTTCCCTTTCAAGCGTTGCTGGCCTACGGAGTCTCCGTCCACGCCGTTTGCCCTGGAAAGAAAGCCGGCGACGTCTGCCGGACTGCTGTTCATCAGGGCCTTGGTCATCAGgtcagcttctcggctttcatcactcaaagtagctcaatttttttggtgcgaaatggaatcaaagattcttttggattttgtagtctgcaaaagatttatgctacgaaattttctaggaaatagaaaagattcgttttggtcgattcaaatttgcatctgcttagtcatccacagtcagagatgtttcatttgattatgggtatcgcagattatacacttccatgattagattcagttttattttttgtgggtgctgtgtaaatttcaaattcgagggttttcgcacttgttgcttagtgaaatcagtttatttttgtgtgggtgtttggtatatttcgaatttaagggcttacccaattgttgtttatttaaattaggggtactgctgatcattggctctattgcatcacatttagcttgtttctttaggatcggtgttgacctatgatgtccagggagcaaagaaactgtttccaagggagaatataatttttatggaaaagaaaagggagactactggattagttagcagaaattttaaacttttttctgctaacaggaatagaaagaacgggaattacacagaaaaacgtgtgttggtatcagtttttgaaatcagatcatttttaagaaattctagagctatcactttccagattttttttagaaaagcaatcaatttgatgattgaagcaatccacaaataaacatattcactgtttgcataatcaaaattgaaaattataattgttaaagcgtgatatataccttgtgggctcaaatcctaataagtttttacaaacattggtaactcaacaatggtgtttcttaaaaaaattcatttttgtgtcattcaccaaaccacacccacattcttgtgctggtggaggagggctggatgcgttaccgattttttcaggtgtatggcttatataggagatagagacatgattggataacctttttgttccctattgcttggtcataattgtggctctggcagagtcatctcgattgtgggggcaatgctatgacactaggtatgataggctcaagggatactggatgggtaccttcagaatcctataagtcttttgaagaaataaaacattggtgaaaatagactcatgttggcactttttatggttcttccagttaatgacattttaagaggactaaacttcgtgacattagattgttgtattcctttttttcgcatctctttttatcattttttcttatttttggtggttgcagACTTATTCTGAGTCACGGGGTCACAACTTCACAGTCAATGCAACCTTTGATGAGGTTGACGCAAGCAAATATGATGGACTTGTTATTCCAGGAGGGCGGGCTCCAGAATATCTTGCAATGAATGAATCTGTTTTGGACTTGGTACGCAAATTTTTTAGCTCTGGAAAACCAATTGCCTCTATTTGCCATGGGCAGTTAATCTTGGCAGCTTCAGGCTCAGTCAGAGGACGGAAGTGCACAGCTTACCCTGCTGTGGGACCTGCACTCATTGCTGCAGGAGCTCATTGGGTAGAACCTGAGACCATGTCAGCATGTGTTATTGATGGTAACCTTATTACTGCAGCAACTTACATAGGTCATCCTGGGTTCATCCAGCTTTTTGTAAAAGCACTAGGAGGCACCATAACTGGTTCAGATAAACGGATTCTATTTTTGTGTGGGgtaagttttgacttcctatgataactttgagctggaaaaaccataccttgtttataatggaacctttgttctttttttcattgcatttttctgtgacctatatgtaactagttcaatattggtagtgcaccaaaggcataaacaaaggaaagggaaaaacaaatgcttgtcctctaatgatcacttcccgtgtgcatcatcaaagcacacaggcatagatttgaatttatgaatttggtgctttaattagtgtaagagtcacaagacttaagactgcaacttcactggttgtgtcctttggatgaggaatgagaaaccttcaactaaagttgactgaaaatccttactaattagtatctttcacccttcctacaaaatggagttagttttcaattgtttttggcatgaatgttagctttcatagaatgctaagtctttgatgatgatacttgttgagctaccaatttgatccaaaaggaaatgttgttagataatgtttggccaacaaaatatttaacaccctatgtgatatcctgcattggtcaagaagaaaagttcctattatagatgtgattttcttttaaatatttagatttgttttaaagttgtacaaattttagtcccaaaacggatattatccacatgggagggaataggtctttataattggtatcataatcgatccttgacctgatgtgatagtttgtttatttgattttacaaggtgtgtctatccatttgatcccataatccccataggcaagacaaggatgttttgcctgcgaggtgaatgtaatatcccacatcagtgtgttggaaaagttcctagtattatatatgtaatgagttcattcattttaactatgtagattgttttaaagttgtgcaagctttgatctaaaacccatccaccttgtaatgtccatcgaacatgatgaagatgttttgtttgcagggtgattgtggtatttcacattggctagaaagaaaagttactaacactatatatgtagtgagtttctcttaattgtgtatatatgttttaaagccatgagagctttagtccacaatgggcaatattctttttgaaaaattctggagagaggcaaaggcttatccagttggataagatatagggaatttagtttgttgtgtttgctagaaggagtggagttttgttgccaaaaataagggaagcggtttagcaagatatgaaaggaatgacaaagggggtaaagttggagcaacacaaaaattgggcaaaagattcgtcaaatgcttagctttgttgaggcgtagaagtttgttttggttttcctaaaaggcagaggtgtcctttagggatgggctattcttgcaaggaaacttcttgtcttagagtggtttctttctttcaagatactaggagtgttgcttttatcaaaggacctatagtagaaaggagcaagatagtgtctttgttaatagtcttccagggacaaggtgaagaatgtttagggtgcaataggagatgcggttgggatttagtaagggagcaaggaggttttaaggaagatgactctagaattgtagactttgaacaggtgggttggtcataattggtagctgaaaggtgggatgaatttatccttgctagggggagctctcattttgttcgagtttgatgaaggtgcaaatgtggagatggtgcttcactatgatgtaaggaggtataaagataaaagagttattcttggatagataggtgcttgaggtacctatcagaaaaaaaaaaaaaaaaaaagtaggtgagtgcttgaggtggggtgcttttggaacaacatccacataaaggaagtgtgggtgatagtaatgggacttccattgcacttgtatgatagtatgttgtttaaaaaattaagggattggtatggaggattcgttggagtcgatggaggcataactgtcacaccctagattttagacttattaatactcaaatacaaaattagtgctcccaaaaccggggagacaaaatttaaatgttgagtttctggttgattttgtgctaaatgaagacatggaaacctgttataaaaaagtatacatcctatgataaactttgtaaccatttattaacaaaccgtttcacaaaagaaaacctgtaccatcttactttttacataccaaaacacaatgattcactaagggtagcctatactacaggtgtaaaaacaagtatacattttatcattccttcaaaaatgatttagtctttgaatgatacaaaggcaaaagcctccaaacactttcaacacgaacaaaacaacatccaatcaatacaaaagcaatactcaagcattaaccccaactgatgtcttctgacttgtatctaaagggggaaggaatagggtgagttcataactcagtaggaaagcttataaccatcctgtgcgtattcttaaaaaccttgaattaaacatataatagcatgcatgataaacaatttataaccattagcaaatatgcaataatgccaaacatgcatgcatgttattgatggttttatttctgtttagcctcatccatcatctcacacctgataagctactaacccccattaatgtacatatcaaatatcaatgctccaaaagttattcggtcataataatgactattctggggactatcgtccaagggtaagtcataccccatgtcttctgggactcatacccaatggcaggaccaaccccatgccttttgggactacaacccaagggcaggaccaaccctatatactcacattggagtgtcttcctcgagggctttagggactttcacccaaggacccacgatcctacgtaaacaatatatccaaagatagtgcttatagcttcacataaacacttcccacatatcaatctattttctggatattatctgccattattccatattgtttttgcaatgcaaccatatcatgaaccattttctcaatattgaacccatggatcctcataccaatacatatccaaatatcattgcaacattttaaacaataaactgagaacaccatgacacattaaagtacttcatgaaatcatagtaatgacatgaaaattccaataaatgcttcaagaaattacaccaagatatacaatggaatggtatgaacttccctaccttacgccaacttctcttttgcaatccttctatgtggacacccttagctattacaaggaactgaaatgaaacaatatagtttaggttttctagccataaaaatttcataattaaagcttacgacattcttcccttattgcgaatttttacaatttgatgttgatgacttaactttccatattccctaatcatcatgcctgcaggtttaaaatctaatttaaggtggattgaacaatctttgctatgcatactgatgtctgccaattaattagtatacactacttactttgattttgctccaagtattcaaataaacctaattaatcacaggattcattaatttatttttttcaattaaagcaaactaaacaaacaagcatgctgaatcttccatcaagagaaatcaaaatatttaaaaccctaattaaccttgatttataattaaaaaatgtttatacctaattaaactcagcttttaaacaaactttatcgttaatttgcttcaaaagatcatttggaacctaaatctaccaaaatcactaaatttattttcttctttaccttgattcttcactttctctatctagattctctctctaaacccaccagcttgttgcagagacaaggctggaaatgagggttggtagcccttataaaggggtcctgaagctggcatgcatgagatggcaagccctaaggctgccactaacctcaagtttcctatatttccgattaagtccctctagttataagtttttatgtctatagctgcacattagtcccttaagttttcataattctaattaacccctaagaacctaataagcatacttaagtcgttaactaaccatacttaaccttaatcaaagtttaattcacaattaaacaagattagcgttaaactagtcttagcctctaattaagcacgtttaaagttaagtactaagataatcattattactaggtattacaataactatgttccaaaaccttcaatgggcaagaatccttgtgatgttagatgggaggaaggtaatctaggtctcctcaagtggtggtgggttcaaggtgtttcgtgatttagctgtgttgggtagttcctccatggttgtctttggtggtccctaaggaggatttcaaggactaagagatttgggaggaagatagggacaattcatgtgctagagaaagagtgatcaataataatgaggatggtatgaaaggtgtaggagatgtgtcattctttggtttaaagagggctagaaatggtggtaaacgagtggagatttaaatacaaggatggttaataggttaaaggatgaagtcttggaaggtgacaatgggctctatgttgaaaacaagcttggatgtttgcgtattgtgggtttgagagcataggattgtttctaggctacatcaagcctaggattgctaggttaggtttagtctatttaagggttggttttagagcattccattatggtatggataggtgttagtgggcttaggtgggtctggtgcccgtggaagagatgaagaggggatgttgtctaagagggtgtggcttgatttttcaaaatcaataaagagtagtatctcttcggtcaaaaagggaggacactcctactttcgaagggttaaggatgatgcatgaggatctagtggttggttccatttgtactcaaatggcctcccttgtttcgaccgctttgatgttgtcagaccataatacctcatgatttgatgtggtgcttttaaaggaagcttctaggtttacaagcaatcctttatactttgtgaactcggttgggggtagggcttcgagttctctttctatctctaaggggcttaacattaagtggaggattgtgagcctaaaggggtgcaaagatggttttgagcgtaaggacaagatgcctttgagaatattttggcaaatggaagcctggtggagttttcatttgttagggagaaggtagatggcaatttgaagggtgtgaggacaacggacaaggatgtggtagtacaagaggggtttgagcatggtggctttcaacaatttctctgtcttgttgagagaggggcttgaattggagattttgagtctcgttaaagagatgaaaaagagaggaattaaaagcccagattggtgagaagaggaggaagaaaaactgttcttcctcatttgaagggggactaaggaggttggaatgttctatgaatcatataggaaaacttaaagggagtgagaatggaaaggatggctaggagcttgctaaagtttgccaatgaagttaagaattcgttcatggaatgttagaagggaaaatgacaagcagagaaggaaggttgtaaagtccctaattcaatcattgacgatagacttggtgtgtttacaagaaataaaaatttagaatatgtcggtggagatagttaggagcttaggaatgggacgatttttagattggggagttatgggagctaggggtgcagttggaggagtgatagtcttttgggacaagagagtgctatagcttgattgcatgggagggaggagtttccaattttatgttgttttaggtgaatcaaagatggcttcatttacgttactttgaggtttacaaaaccaactttaggggaattaaagggtttgtggactgagttaggggctataaaagggttgtgggaagatccctgtgtgtgtcgaaagagatttcaacatagctaggttccattcagtttttggtgtttgaagagtgagaaattcattatagagaggtggtgcagtgtactttactaaggcctgtatcatatcattcttcaattcttttagatggtaaagggggtaggtagtattccattcagtttgaatatatgcagttgaagatagagggtttctaagactttctttaggagtggcagatggtatataatttcaaaggttcatacaacttcattttggctttctaattgaaagtgctggaaatagaatgggtggaaggtttttggtaatgtgttagagggtgtttggtaaatcaacttaatgacttaaagtgactaaataacttaatttaagttattaagcaagttaagtatgtttgataaaataacttaatatcacaacttaaagttaacaatgactgtaagtattaagtcaaaatagtcaatttagtcttaatccctttttttttctttgttttatttgcccacaactaacaaaaatatatttaacaactatagcttcagattcattactcatcttttatgaaaataaatattaattaaaagttttaataataaacattaattacaactaatgaggacaagtatgtcaatttaataacttaaaataaattttaagttaactataccaataaccttaatacttaaagtaaaaaataagtaataagttttaagttaacaatttaagagcaattttacttaaaatcaacttaagttattaagtaatatgtattaagttttactaaacacccacttagtcaagaaggagatgatttttagtcaagtgtgctagtgggattctaaggaaaaggaaggagctttttctcttaaggaagccaaagctagaagtgtagcaagggaagaggattgcatgtggatgtttttggaagaagtctcatacagacttaaatcaagagagatttggctaaaggagggtgacaaaaacacaattttttttatttttttatttttattttattttttattttatttttataaaatggataatgcacataagagaaggatttttttttgccaaaatcaaaattattgggacttagccatatgaggactaggaggtaaaggaagtagtttaggctttgcaattgtttgatttaggggagtggagacctagcactatgggatgtttttcaaagccttaaacgatcagagattgaaggattagaggtgcctttcactaaggaggaggtgttcaaggttttgtcaggcttgaatggggataagactttaggcttgggtggttttcctatgctttctagcaatttagtagagactttatgaaagacaaagtgttggggctttttagggagttctaagacttaggaaagtttgagaaaaaatggttgaatgctgtgttaatttttttttttctttttttaaaaattgtgttaatgatctaatagacttttagaccaattagcttggtggattgtttgtacagctcctagctaaggtgttggcaaataagttgaaggtggtgggaaaggtggtttttgatttccaaaatgttgttgtagagagaaggtagattcttgatgcaacacttaccgttaatgaggtaattgatcctatgatgaagagtggctccaatagtatagtttgtaagcttgacattgagaaggcatacgaccatgttaattgaggtttgttgctaactattcttggaaaaatggggtttgatcagaaatggactgaatgattaaatgatacatctcgacaataaagttttccttgttggtcaatgatgctcctttgggtttttttgaaagctttagacgcttgagacaaggggaccttctctcttcttatgtatttgttttggtgatgaagacgctcagttgtctattggggagggctagggagggtggtttttttgttggcttttaagcttgtgagggaagggaagtgagggggtggaagtgtctgatctgttgtttgccaacaccaccttagttttttgcgaggctatgcaaattcaaatgatacacttaagttgcttgttcatgtggtttaaaggtatctcaaggctgaaaattatccttgagaaaagtgaactgatcctaatatagagggtggctaatgtggaggatttggctttataattgggatacagtgttaaggagcttccaactacttacttggagcttcctttaagggctccattcatagcaaagtcagtttaggacaatgtgaaagaaagatttcacagaagattattgtttcaaagtactttgtgcagtttgtctatttattttatgtccttgtttaacatgcttagaaaggtcagattgagggttgagaaaatttagaaggtactttctttggtgaggtgggacttaagagagaaagatgcattagtgaaatgaccaattgtttgcagagagagaagaaatagagctttggaagagaggatgtcattggcaaatagaagtctcctttgttaaggcaaagaggaatcaatagatcgcattcttctacaccatgccaaagtaagggaattgttgcaaattttattctctttgttaggcatcgcataggtccttccttctatggccaaggagattcttttgagttgccatgattcttttgttggaaaaaaacgcaagaagcaagaaggtttagaaagttagtgtgctattcattttttcgacatatggaaggaaagaaattgaagagtgttcaaaagtggagagcagccagttcaagcactaaagtgctcctttccttagaactctagtgctatgggtttgcatgtacctggaagaagtctcattgcctttaattgattttatagatagtgggggatgtaattgtggggagggggtagttatttttatgcgctctctttttctggcaatgtcttttggtacctattgtatactccatgtgtagcttggtgtatagcttcttttttatatttatgatatttgcttgcagacttatctagaaaatggaattattatatataaaaaatggaaatataagaacaaagatagctcagagaatcctattgtgtctctgttttttattttcttattgttttataatgacgaaacaatagaactctacccttgcattcggattacaaaatttaatcaaattacttacagttgccaatcaccttgaattattaaaattatctggttcatgtatctcacttaacatttaccaacctattaaataatatgcttgcaaaaaattgaaaaaatgtaaaggtgaaattgtttgctaaatatccctatgcctcctgccaatcttattgttgaagaactgcatgtctccattgcaatgctagatcactgcttcgttcacctcataacttcaaaatcaattgattacagtaggcttcccagcaaaagaaacttgtaaaatcttactttggaagatagttaaatccaaacacaaaataagttctctcttatattttggcattcattaacaaattttgtacgccaactctatcaattagattaccagctggtatggacagctgattagttggtttttcacccagcagctcttgtttaggtggattgaatacaaggtttgtattattttttcttaaagcatggaagacacaattgagtgcctttaatttagtacctgggatattctttcttgattattaaatccaaaacttgcaaggactttatttgggaactcccaataaattattcagtcagtgggttggcaatgggtggtttggatccaaattttcttgaaacaccccaaaacatagaatgcttctagtctgcagctacctctcaatcgaatttgtgaaccttccctctgaaaaatttatattggtttgaaagtgcccttacagttaactaggcatcattgcttttgctcttctgtttttttagGATTACATGGAAGACTATGAGGTAATGGTCCCTTTTCAATCCTTTCAAGCTCTTGAATGCCATGTTGATGCGGTTTGCCCGAAGAAGAAGGCCGGTGAGACCTGTCCAACTGCTATTCATGATTTTGAAGGTGATCAAACTTACAGTGAGAAGCCAGGCCATGATTTCACTCTAACAGCCACTTTTGAAGATCTAAACATCCCAAGCTATGATGCTCTTGTTATACCTGGAGGCCGAGCCCCAGAATATTTGGCATTAAATGAGAAAGTAATTGCCTTAGTGAAGGAATTCATGGAGGCAGGAAAGCCAGTTGCATCCATTTGCCACGGACAACAGATTTTAGCTGCTGCTGGAGTTCTCAAGgtagctttattgacattgttctgaaaataattaaatccttcaggaagtttgtaactggtaaaacctttatgctgatttttatgctggttttaagcaatagttatgatgcatttccaaattgactgggttctgattgctcctttatgttagagggtcaaggggggtgaagtaatatgataattagaatgctgtgaatattgttttttgtttgggtttgtacaaggacttcttgtcttcctatgggttcttttcttgcttaaaattattaattaaccatagataagggagagagagaaaaggagagaaccaaatttgtggaatgttgcatgtatctggtccaacagatttagtttcctagtgagatatttcccaaattgctgcatagatgtatctgttaagttttgatgctttactgatttattgatattcatgtttgtctattttattagttgtgttttgaaaatctgtggaattaggagtttcagtaagcatgattgccatattctgattgtcatttatgaactgttttatagGGAAAGAAATGCACTGCATATCCTGCTGTGAAACTCAATGTAGTCTTGTCTGGGGCTACATGGTTAGAACCTGAGCCAATAGATCGCTGTTTCACAGATGGAAATTTGGTAACTGGAGCTGCTTGGCCGGGCCATCCAGAGTTCATCTCTCAGTTGATGACGTTGCTCGGTATCCAGGTCTTGTTCTAGttatttatctatgtgctggcctgtatgccttaattgtctattgaataaatagattcggtttacgtatcagaatgaggaaactttttaaattttggtgttgtgatatcaatgcagtctata

>VvGLYIII-like3

ttgattttttttttgagaattattttaaatttatttatttttgagagaccatttccattttcaagcatatccacaagcaagctgccactgtaatgcttccagcagcacgcgcgctataaaATGGCTTTACGCCACCTCACTCCACTGAGTCCACTCTCTCCCTTCACACGCATACCTCCACGACGTTGTTTCACTCAGAAGCCCTTCTCCCTCTCCGTCTCGGCATCCATGGGTTCTTCTTCTCGTAAGgtactgatttcttctttggagaagaagcgcttctgcccgtcagattcgtaatttggataaattttttttggcttttttttttctttttctggtttccttattatagGTTTTGGTTCCGATCGCCCACGGCTCGGAGCCGATGGAGGCGGTGATCATCATCGATGTGCTCCGGCGAGCTGGCGCCGATGTGACCGTCGCTTCTGTGGAGAAGCGGCTTCAAGTTGATGCGTGCCATGGCGTGAAGATTGTTGCGGATGCCCTAATTTCTGACTGCGCTGATACTGGTTTCGATCTCATTTCGCTGCCGgtattatctctatgtctatctcccgcattttgttcggttggtgggaaaaaggaattgggattttgaaagtcgagtattgagtttgtggatatttgggatccgagaagatatgatatttctactggattgggtctgttataaagtagccgaatcggtaaaaactaaaaagtcgtggttttgaattttcatttccagaaagggagagttttagttaatacatacattttcatattattattattgttgaaaacagaaaaaattgttaactacgcagaagatcagttggtaggatgttttggcctgaaatgcggtgttattggaaatgttaggtctttatttgtttttcttctacctttttatttatttatttatttttggtgttctgaaagggcagacaattttaagaaaagaaaatgatgagtttctgggacgttacttttttccaaatttggaaaatgggatgagattgattttgttattgagtttggtggagcgaaactctttccctttcatcaatgcttattcttgttgttgataacttgagtttacctctcaattgaaagttttggtattttttggtgtgtttggatggtacttttatagaagagcttctggaggcaactttatagtagaatttctagttaaatttttatgataactatttgtgtcaaccattctgcaagtttatggatgatgtttgtgttagaaaaacgtcatgtaatttatgtctggggagggaacatgtaccatctcctttttgtgtgaggctgaatgggaacatttggagttgaaaggaaattgttcattcataattgtacaatatttatactattcaggtagatatcttaactatgacacctgaaataggctattgaaattattttaactgctatcagaattcagaacatttggatgaatgttctctctctgtgtatgtgtgcttgttgtgagtttggtctgttaaaatgttttagattgaaattggggctttacatcacagGGAGGAATGCCAGGTGCTGCCACTCTCAGAGATTGTGGCATGCTGGAAAGCATGGTAAAGAAGCATGCTGCTGATGGGCAGCTCTATGCTGGAATCTGTGCGGCACCTGCTGTGGCACTTGGATCATGGGGCTTGATGAAAGGTTTGAAGgtaagaagcacttgattggtcacatatcaatattttgtttctgggtttttgctgttataattgttgtcattgttgattggatgttaatgacggactagctttgatgtagaaccactttgggggatggttgtctaaaatgaaattggtgggctagggattcacttttttaggatttaaggagagagagggaaaagaaaataaaagacaggaaaggaaaaaagatagaaaagttaaagtttcactaaggtcttctaagaatctcacccagaagagaaacaatggagcatgactattggaaattgcacaatatgctgaaaaattggtattatttatcaatcatcaattaatctcattttaaagcaattagctatatttatagactttagaaattcataaaaattgaatatgaccatgaaacagaaacttcatttatctgcaacttattaggaatctcattttttccctcaaatcatgacactttccacaaaatagaaactttttagaaattagaatttttatgaaatagaaacttttagaaataaaaattcttttttttttttccataaaaaaatagcttttggaaaataggaaatttgaagtattgaaacttgttccaatggcgaatttttaaagaaagttctataaatttaagtgaagactcaaataaccttttttttttttataagtaaagaaaagtatatactagaaagaggcaagactcaaataactattaaacttgactttaaataacttagatcagaaaatttttcttattcttcgttttcttccttgtatagcacttggagattcacctcatcgagtttcttagttactgcaatgtttgttttcaatagGCAACCTGTTATCCATCATTCATGGAGCAATTGTCTTCCACTGCAACTACAGTTGAATCAAGAGTCCAACAGGATGGCAAAGTTGTGACGAGTCGTGGACCAGGCACTACTATGGAGTTCTCAGTCTCTTTGGTTGAGCAATTATATGGAAAAGAGAAAGCTAATGAAGTTTCTGGGCCACTGgtatttagtttctgcttttgtcctttattttcattgattatattttacatgtgcatcagaatatgtggacttttctgggaaagtttggtccgataaaaattcttaagtttgggtgataaaaattattgatgaatctaaggaataaatctgtctctcaaaatgacttggtgggagtgttaggaagggtattaatgataataactgatacagtgctttacttggtgctaatgaagattttaggaagtgaaaaattggtaaaataaagttcctgaggatataatctctccagctatcaagcttttgttcattgagtgcaaaatggtaagaagacatttatgaacttgctagagtgtggcaaaggaccatttgatatataaattacgttatccccttcttggcacttttaaatattaattttatgtacttatcaaaaaaaaaaatctaaattatgttagatcatagaaatggatgttagtttttatattggttacaaacacttctaggatggcgctgaaatatttgttatcttcctgttgaaagagcatataataatgtaatcgcatgtagatcctatccaatatttaacaccttagtcacccaaaagcttgatagcttgacaagatattgctagtggttgcttgtgtaaaagtgggataaccaggcatgataattctgtttgaattttttattttattttttgtatgactggatgtatagttcatgccattgttaaaatatgaaatgtatttgtgatttttcactattttttgcatgttaaagcatacaaaactgctagtatctagttgaattggggtcaactgatacttaattgaacctatacttgaaaacatggtccctggactggtgcacatgaagagcacatctcagatctaatgcctcataatatctctcagatttgtgctgatgtgaaagaatcattctttctattaaactggtagtctatttaccattgatgttgtatttgggctccagaacctttaaaagatgtggatgaggggatttctggaatccatttcattcccccctcttcttaaccaaataggagagtccccaaaactgcatttctgtccaagtatgtttctaccaaattgattcactgctgtctgtattgcagtccaaatctgtggttattaatttgatataatgttgcattgactatattcaggtaattttgtcttatgggaacagtttctggtgtttacctgagtccagatgatagtttacagtaccttgcatattatcaaattttgtaataatccttgaaagagttttgatagattttctggttggacaagtgaaaaacttacaatgtgtacatgtttaggttattcttgctcattgttgcatgttgagttgttaccttaagttgatgttcaggactcctgtttcagcccttggaccctcatcttgaatgaagtagtgctttagcttctgtgattgataagtgagaggttgtagctgtatatggtgaggttatggtgtttgaacttgtggttgactaaaacatacatggttgaggaagttactctattgagttggcagcaatagcaatattgtgttgtgctcatatgtacttgtatgctcctgtcctaatttaatgtgatatgtcattaccggaactctctgatgctgtctgctgtcataagcctcgagtgataatcttaacattcaccataatgggatatttgatgctgaatagttggaaactgtagctgctgccttaacactcaatttataagctaaacttttgctattgttgaactgcaatgcatgatttctggaaacatctatcaacaaatagttttgacttacatatctgtcgagttagttgcacttctgcatatgcatttcatgttattcaagctcttgctgttctgtagattctgttgtaacagacatcttcccccctggtatattcagGTGATGTGTTCCAATCTTGGGGATAAATTCATCATGGCTGAGCTAAATCCGATTGATTGGAAGTGTGACAATCCTCAGgtgagatttcgatagtttctgttataattttttagtgcttctgttttttctccttttcttctgttttttctcctattctctggcccgttgactacccgcctcacagctacaaccccaagcacacccataaccgacacacttgcccaaaacccaccttctgtgccccctttttctctctcaagttgccatacaatgtgcagattttggatggtgcagttatgaatgttttgagctctgcatctcttcctttctttgtttttctttttcttatccttcttctccttcatcaggttatgcttgttatatcttattgctgcatgatttatccaagcatttggttttagtgatgtctatttaaaactgcatgcatctctgctatatgcatggtcatgtttcactgattgccatgatatctgcctgtatatttttggccttttttttcactttattctccactgttcctcaccatatctcccttctttacttcacttttccagggctttgtattctgcttctttctatgactgggcttcaacttggcctctaaaatttaaatatggttttctgttgtggacactatctcctgctttttatgtttttattccatttgtctcataggaacccgctagttccttcacattctagttttctagttgcacaaaagtagaataaaatgaaatggaaaaaaataaaaataaaaaggagaagaagaggtaaatgaatggggatgttgaagtcttttaggatgccttttattttataaatttctggatccttccgagcacaagattatcatgcttaatgtctctaaaatgaaacagcattataaaattcaatatttagagcctacatttcgggttcgatgatatcaccacaaatttaatatattttattgttcatccacccaaatttacattttttatatctcccaactccacaggcaagtgtggtttgactaagcagaattaaagctgttgcgttcatactgtacttatatattgcttacatgcaaatacaagttgcctaagttctgtatttccttttcttaggctcatgtgacttttgcattatttctaaaatgaaacttgcatactaatactgcagatattcttaaataatgagatggtatggaatctgatcttccgcttcttgttgtccactgtataattatgatatgtctcataactccagatattttcacctaattggaagcaaccgttctttgagttgtggataattaactttagtttcctttcagATTCTTGTGCCTATTGCTAATGGCACAGAAGAAATGGAAGCTGTTATAATCATTGATTTTCTGCGTCGAGCAAAGGCAAATGTTGTGGTGGCCTCTGTTGAAGACAAATTAGAAATTGTGGCTTCACGAAAAGTGAAACTAGTGGCAGATGTGCTTCTTGATGAAGCTGTTAAACTTTCATATGACCTAATCGTCTTGCCAgtaagttgggtttttcatctcccatttgtcattaccaaccaattaagcttaatcttatgctaaatatttgcagGGTGGACTAGGTGGTGCCCAAGCATTTGCCAGCTCAGAAAAACTGGTGAATTTGCTAAAGAATCAGAGGGAGTCAAATAAACCATATGGAGCAATATGTGCATCCCCAGCTCTCGTCCTGGAGCCCCATGGCTTACTCAAGgtagcatcgtttactgatttttttattcattattctcataattgggaccaaattctccaaattattattgttgttattattattattattattattatttaaaaaaaaaaactatgcatgaattcatcaatgttatagaaccggattttataacatattcatcattcttgaattaggcattatttatgctaatgggttcctcatatatgtcaccaaatttattccccatttttttattaaattcttgtcaatattctgctttaccatgtcccatatattttctgaattccaaattgttccttccactgaataattcttgaacttcagctttagattccttatattcagtgcttgtacttgagaaatataatctgctcttatccagGGCAAGAAGGCCACAGCTTTTCCTGCACTATGTAGCAAGCTGTCCGATCAGAGTGAAATCGAGAACAGGGTTCTGGTTGATGGCAACCTCATTACCAGCAGAGGCCCCGGAACTTCCATGGAGTTTGCACTGGCAATCATTGAGAAGTTCTTTGGCCACGGTAAAGCACTAGAGCTTGCAAAGGTAATGCTTTTCTCAAGCCAGTAAagagtatttcccccctactactgctgaagttagtagaaagactgaaacgaaaatctgaatgcatatttgaatatgtcgttcagttcatggccatgcgcagaagtcttgtttataaataaa

**3. Other protein sequences used in this paper.**

>AtGLYI-1 (Accession No. AT1G07645)

MAANMMRPAFAYTVVYVKDVAKSVEFYSRAFGHNVRRLDESHRWGELESGQTTIAFTPLHQHETDDLTGKVQATQSARERAPIEVCFCYPDVDAAFKRAVENGAEAVSKPEDKEWGQKVGYVRDIDGIVVRIGSHVK

>AtGLYI-4 (Accession No. AT1G15380)

MKEDAGNPLHLTSLNHVSVLCRSVDESMNFYQKVLGFIPIRRPESLNFEGAWLFGHGIGIHLLCAPEPEKLPKKTAINPKDNHISFQCESMGVVEKKLEEMGIDYVRALVEEGGIQVDQLFFHDPDGFMIEICNCDSLPVVPLVGEMARSCSRVKLHQMVQPQPQTQIHQVVYP

>AtGLYI-5 (Accession No. AT1G64185)

MATASFRWILQLHRDVPKAARFYEKGLDFSVNVVTLRWAELQSGPLKLALMQAPSEHVMSEKGYSSLLSFTVADINTTISKLMELGAELDGSIKYEVHGKVASVRCLDGHVLGLYEPS

>AtGLYI-7 (Accession No. AT1G80160)

MKDETGNPLHIKSLNHISLLCRSVEESISFYQNVLGFLPIRRPDSFDFDGAWLFGHGIGIHLLQSPEPEKLLKKTEINPKDNHISFQCESMEAVEKKLKEMEIEYVRAVVEEGGIQVDQLFFHDPDAFMIEICNCDSLPVIPLAGEMARSCSRLNIRQLVQPTQIHP

>AtGLYI-8 (Accession No. AT2G28420)

MEEKKKKGDDELNSKPPLMALNHVSRLCKDVKKSLEFYTKVLGFVEIERPASFDFDGAWLFNYGVGIHLVQAKDQDKLPSDTDHLDPMDNHISFQCEDMEALEKRLKEVKVKYIKRTVGDEKDAAIDQLFFNDPDGFMVEICNCENLELVPCHSADAIRLPEDRHAPPVALPDSSNRRMPQPNS

>AtGLYI-9 (Accession No. AT2G32090)

MASLGHIARESSDITRLAQFYKEVFGFEEIESPDFGDLQVVWLNLPGAFAMHIIQRNPSTNLPEGPYSATSAVKDPSHLPMGHHICFSVPNFDSFLHSLKEKGIETFQKSLPDGKVKQVFFFDPDGNGLEVASRS

>AtGLYI-10 (Accession No. AT5G41650)

MATASFRWILQLHRDVPKAARFYAQGLDFSVNVVTLRWAELHSGPIKLALMQSPSNHVAEKGYSSLLSFTVTDINTTVTKLMALGAELDGTIKYEIHGKVAAMKCPDGYMLGLYEAA

>AtGLYI-11 (Accession No. AT5G57040)

MASIFRPSSASLDLRPKVICTNLSTKERFEFQKKSVRKERINVRFYSLKAKAQGSSIEGISVVQEKELNNKTDYGVVGVHHVGLLCENLERSLEFYQNILGLEINEARPHDKLPYRGAWLWVGSEMIHLMELPNPDPLTGRPEHGGRDRHACIAIRDVSNLKEILDKAGIAYTMSKSGRPAIFTRDPDANALEFTQV

>OsGLYI-1 (Accession No. LOC_Os01g07850)

MVNTTAGVKCGGGGAALPLSTLNHVSLVCRSLSTSLTFYRDFLGFVSVRRPGSFDFDGAWLFNYGIGIHLLQAEDPESMPPNKEINPKDNHISFTCESMEAVQRRLKEMGVRYVQRRVEEGGVYVDQIFFHDPDGFMIEICTCDKLPVVPLDAAAAHSIFAGRSPPPPVACKIRPVKQPSATKLGSVAAGGCVGEVIVVDAINGAAAAGGGGAMS

>OsGLYI-3 (Accession No. LOC_Os03g16940)

MTMTMSNEQGKPEANVRGGRRSGHRVHARHLPGLPTLAAAPARTNGSSWAPGKTEHCTAQHKRRLQAVRDKPQQASVMASEGAVSPAFAYTVVYVKDVAKSAAFYSAAFGYTVRRLDQSHKWAELESGTTTIAFTPLHQRETDALTGAVQLPDSAGERGPVEICFDYADVDAAYRRAVDSGAVPVSPPEQKSWGQKVGYVRDIDGIIVRMGSHVRA

>OsGLYI-4 (Accession No. LOC_Os03g45720)

MATLQLNHVARETDDVRRLAAFYEEVLGFERVASPNYPAFQVAWLRLPGTPGVALHIIERDPAAAPAAVAPGAAGAPPAQLPRRHHLAFSVADYDGFLTGLKARGTDVFEKTQPDGRTRQVFFFDPDGNGLEVTSSGTGDM

>OsGLYI-5 (Accession No. LOC_Os04g45590)

MGSEAPDPAVAASVPLVRLNHVSFQCTSVEKSVDFYRRVLGFELIKRPESLNFNGAWLYKYGMGIHLLQRGDDADGCSIPTRPLPAINPMGNHVSFQCSDMAVMKARLRAMDREFVVRKVWDGETVVDQLFFHDPDGNMIEVCNCENLPVIPLIVASTPGLPELLPPAMQTNVHG

>OsGLYI-6 (Accession No. LOC_Os05g07940)

MVNTAAVAAAKGSRGSGLPLASLNHISIVCRSLQESLTFYTDVLGFFPVRRPGSFDFDGAWLFNYGIGIHLLQAEDPDSLPGKTEINPKDNHISFQCESMVAVERRLKELGIPYIQRCVEEGGIYVDQIFFHDPDGFMIEICNCDNLPVVPLGADQPLVMAACKRAAVIKQQQQASSSPATAAAAAQCAVPSSTKAIHVGEEAHISCA

>OsGLYI-9 (Accession No. LOC_Os07g06660)

MAARCLSSLALLSPSPSSSGKVSAMASPPVPSSAAPRRRPGTRLSVATGGEQLVTAQEASQEPAYGVVSIHHVGILCENLERSMAFYKDLLGLKVNPARPTDKLPYRGAWLWVGSEMIHLMELPNPDPLTGRPEHGGRDRHTCMAIKDVLKLKEIFDKAGIKYTLSKSGRPAIFARDPDGNALEFTQV

>OsGLYI-10 (Accession No. LOC_Os07g46360)

MAGCRRPTTEMGEVCKRVAPSVREEEEEEENGDGGGVDPAAESSSAKLYEDVPEMPLMALNHISRLCKSIDASVRFYVKALGFVLIHRPPALDFNGAWLFNYGVGIHLVQRDDARRAPDVNPGDLDPMDNHISFQCEDMGMMEKRLNEMGIEYMKRTINEEEGSPIDQLFFKDPDGFMIEICNCENLELVPAGALGRLRLPRDRHNPPLRMAAAGNDEA

>GmGLYI-2 (Accession No. Glyma.01g168400)

MKMEIEEVGNCEALPLLSLNHVSLLCRSVWVSMRFYEDVLGFVPIKRPSSFKFTGAWFYNYGIGIHLIENPNIDEFDTCVNEERPINPKDNHISFQCTDVELVKKRLEERGMRYVTAVVEEGGIQVDQVFFHDPDGYMIELCNCENIPIIPISSCSFKPRGHSFKKAAPNKCGFMENVMMESLSTDMINFSF

>GmGLYI-5 (Accession No. Glyma.06g084500)

LPQAQLFGAEKIAQPEKNLFDWVKNDNRRFLHVVYRVGDLEKTIKYALLRKRDIPEDRYSNAFLGYGPEESNFTVELTYNYGVDNYDIGSGFGHFGVAGRLITREPGPVKDGSAVIALIEDPDGYKFELLERRPTSEPLCQVMLRVGDIDRAAGMKLLRKRDNPEQKYTVAFMGYGPEYMNSVLELTYNYGVTNYDKGNGYAQIAIGTNDVYKTAEAIKLCGRKIIREPGPLPGINTKIVACLDPDGWKLAFVDNVDFLKELE

>GmGLYI-6 (Accession No. Glyma.07g031700)

MKESVGNPLRLQSVNHISLICRSVEQSMDFYQNVLGFYPIRRPGSLDFDGAWLFGYGIGIHLLEAENPEKLPKKKEINPKDNHISFQCESMVAVEKKLKEMEIDYVRATVEEGGIQVDQLFFHDPDGFMIEICNCDSLPVIPLVGEVARSCSLVNLEKMQNQQQIQKMLQQL

>GmGLYI-7 (Accession No. Glyma.07g261400)

MADLLEWSKQDKKRMLHVVYRVGDLDRTIKFYTECLGMKLLRQRDIPEEKYANAFLGFGPEESHFVVELTYNYGVTSYDIGDGFGHFAIATQDIYKLVEHIRAKGGNITREPGPVQGGTTVIAFVKDPDGYTFGLIQRPTVHDPFCQVMLRVGDLERSIKFYEKALGMKVVRKVDKPEYKYTIAMLGYGEEHETTVLELTYNYGVTEYSKGNAYAQIAIGTDDVYKSAEVVNQVIKEVGGKITRQPGPIPGLNTKTTSFLDPDGWKTVLVDNVDFLEELK

>GmGLYI-9 (Accession No. Glyma.08g211100)

MKESTMGNPLRLQSVNHISLICRSVEQSMDFYQNVLGFYPIRRPGSLDFDGAWLFGYGIGIHLLEAENPENLPKKKEINPKDNHISFQCESMEPVEKKLKEMEIDYVRATVEEGRIQVDQLFFHDPDDFMIEICNCDSLSR

>GmGLYI-12 (Accession No. Glyma.09g226500)

KEGKGKEENPPPLLAMNHVSRLCRNVKESIDFYTKVLGFVLIERPQALDFEGAWLFNYGVGIHLCEDLEAMEKKLKEKNVKYMKRTLEREDGTTMDQIFFNDPDGFMVEI

>GmGLYI-13 (Accession No. Glyma.11g075000)

MKMEIEEVGNCEALPLLSLNHVSLLCRSVWESMRFYEDVLGFVPIKRPSSFKFTGAWFYNYGIGIHLIENPNIDEFDTCVVEERPINPKDNHISFQCTDVELVKKRLEERGMRYVTAVVEEGGIQVDQVFFHDPDGYMIELCNCENIPIIPISSCSFKPRGHSFKKAAPNKCGFMENVMMESLSTDMINFSF

>GmGLYI-17 (Accession No. Glyma.12g167400)

MEKMELAETPLPLLSLNHVSFVCKSVSESVKFYEDVLGFLLIKRPSSFKFEGAWLFNYGIGIHLLESEKVPVKKREINPKENHISFQCSDMKVIMQKLDAMKIEYVTAVVEEGGIKVDQLFFHDPDGYMIEICNCQNLPVLPISSCPLKQLGGEATFKINCFAEESMSMLMMDNFVMDMLKISI

>GmGLYI-18 (Accession No. Glyma.13g106600)

MSCCSCSAMAFLLKAPSFLPPLNQKLNYTHKSFSPINLQSKFYHASVRNGRWNVPSMTIKAQAAVEGDVLLDEESICVNEESDYGVVCMHHVGILCENLERSLDFYQNVLGLKINEARPHNKLPYRGAWLWVGSEMIHLMELPNPDPLTGRPQHGGRDRHTCIAIRDVSKLKAIFDKAGIAYTLSHSGRPAIFTRDPDANALEFTQVDD

>GmGLYI-19 (Accession No. Glyma.13g168200)

MANPLQLKSLNHISIVCASVEKSVDFYVNVLGFSPIKRPSSLDFNGAWLFNYGIGIHLLQSENPEGMPKTAPINPKDNHISFQCESIAAVEKRLQQMKIEYVKNRVEESGTYVDQLFFHDPDGMMIEICNCDNIPVVPLTEDKVWSCSRFNCNIQNHQQQIQQMIPM

>GmGLYI-20 (Accession No. Glyma.15g009500)

MKENVGNPLHLKSVNHISLICTSVKESINFYQNLLGFFPIRRPGSFDFDGAWLFGYGIGIHLLQAEDPDNVPRKTKINPKDNHISFQCESMGAVEKKLGEMEIEYVHATVEEGGIKVDQLFFHDPDGFMIEICNCDSLPVIPLAASGNNNGMVRSCSRLNLQILQQIHQFLNQ

>GmGLYI-22 (Accession No. Glyma.16g003500)

MGKMEPLPLLSLNHVSFVCKSVSESVKFYQDVLGFVLIKRPSSFKFEGAWLFNYGIGIHLLESEKVPVEKREINPKENHISFQCSDMKVIMQKLDAMKIEYVRAVVEEGGIKVDQLFFHDPDGYMIEICNCQNLPVLPISSCPLKQLAAGEATTLNINCFADESVSMLMMDNLVMDMLKISI

>GmGLYI-23 (Accession No. Glyma.17g052700)

MNCCSAMASLLKSPSFLSPLNQKLNYVSFSPMTTNLQSKFCRASVRNGRWHVPSLTIKSQAAVEGDVLEKESVSINEESDYGVVCMHHVGILCENLERSLEFYQNVLGLKINEARPHDKLPYRGAWLWVGSEMIHLMELPNPDPLTGRAQHGGRDRHTCIAIRDVSKLKAIFDKAGIPYTLSHSGRPAIFARDPDANALEFTQVDG

>GmGLYI-24 (Accession No. Glyma.17g115900)

MANPLQLKSLNHISIVCASVEKSVDFYVNVLGFSPIKRPSSLDFNGAWLFNYGIGIHLLQSEDPEGMPKLVPINPKDNHISFQHSSGGKRLQQMKIEYVKNRVEENGMMIEICNCDNIPVVPLPEDKVWSCSRFNCNIQNRQQQI

>MtGLYI-1 (Accession No. Medtr1g022325)

MGNETQTKTGFKLVGFKKFIRTNPKTDRFKVKRFHHVEFWCTDATNTALRFSQGLGMPIVAKSDLSTGNLIHASYLLRSGDLNFLFSAPYSPSISLSSPSSTASIPTFSASTCFSFCASHGLAVRAIAIEVDDAELAFTVSVNHGALPSSPPIVLENGVKLAEVHLFGVDVVLRYVSYNNPNLLFLPGFESLLNESSNSSLDFGIRRLDHANANVPELASAVKYIKQFTGFHEFAEFTTEDVGTSESGLNNVVLASNDETVLLPICEPIYGTKRKSPIETYLEHNEGAGFQHLALASEDIFRTLREMRKKSGVGGFEFMAPPPVTYYRNLKNRVVDVLSDEQIKECEELGILVDRDDQGTILQIFTKPVGDRPTVLIEIIQRVGCMLKDEEEKEYQRGGCGGFGKGNFSELFKSIEEYEKTLETRRTA

>MtGLYI-2 (Accession No. Medtr1g115170)

MKETVAFYEKVLEFISIVRPGSFDFGGAWLFGHGIGIHLLLAEDPEKIPRKNEINTKDKHISFQCDESMDAVEKYLKDMKIGLKRAMVEENGIQVDQLFFHDPDGFMIEICNCDSLPVIPLAGGMVTLCPRLNFESMPQQIDQVAKQI

>MtGLYI-3 (Accession No. Medtr2g005880)

MPLPLLSLNHVSFVCRSLQESVKFYENVLGFVLIKRPSSFKFQGAWLFNYGIGIHLLETESDKVPVKRGEINTKENHISFQCSDMKLIMKNLDEMNIEYKTAVVEDGGIKVDQLFFHDPDGYMIEMCNCQNLPVLPISTCPLKQPTNQAPVPFYGEGKNCHAEEALLMMEILVIDLLRISI

>MtGLYI-5 (Accession No. Medtr2g103460)

MDAIVGNPLRLKSVNHISLICRSVDVTVAFYENVLGFVSIVRPGSFNFEGAWLFGHGIGIHLLKAEDPEKIPRKKEINTKDNHISFQCDGSIDAVEKYLNDKKIVCKRALVEENGIQVDQLFFHDPDGFMIEICNCDSLPVIPLAGEIVNSCSRINLETMPQKIHQPVEKI

>MtGLYI-6 (Accession No. Medtr2g103490)

MKEIVGNPLRLKSVNHISLICKSVNESVSFYEKVLGFISIVRPGSFDFEGAWLFGYGIGIHLLQAEDPENIPRKNEINPKDNHISFQCDESMDTVEKYLNDKKIGCKRAMVEENGIQVDQLFFHDPDGFMIEICNCDSLPVIPLAGEMVRSCSRLNLEIMPQQIHQVVKQI

>MtGLYI-8 (Accession No. Medtr4g011020)

MKESVGNPLHLKSVNHISLICRSVEESIDFYQNVLGFFPIRRPGSFDFDGAWLFGYGIGIHLLEAENPETLPRKKEINPKDNHISFQCESMGAVEKKLKEMEINYVRARVEEGGIEVDQLFFHDPDGFMIEICNCDSLPVIPLVGEVARSCSRLNLHIMQNQNQNQQNQIHKIVK

>MtGLYI-9 (Accession No. Medtr4g039890)

MCVKFDRFYTECLGMKLLRKRDIPEDKYSNAFLGYGPEDSSFTVELTYNYGMDNYDIGTGFGHFGIIAEDVSKTVDIVKAKGGKVTREPGSVIGGSIVTASVEDPSGYRFKLLERRTTREPLCKVMLRVGDLDRVIAFYEKAVGMKLLHKIDNPEEKYTVAKLGYGPVLELTYNYGVTNYDKGNGYAQIAIGTDDVYKTAEAIKSCGGKVIREPGPLPGINTKIVVCLDPDGWKLVWHFSIFSLYLM

>MtGLYI-11 (Accession No. Medtr4g063500)

MASKLSPEFAYTVLYVKDVAESVAFYSKAFGYSVRRLDESHRWGELESGHTTIAFTPIHQHETDDLTGVVHTTRSNKERPPVEVCFVYTDVDAAYKRAVENGAVPVSEPEMKEWGQKVGYVRDIDGIVIRMGNHVKPAKLD

>MtGLYI-12 (Accession No. Medtr4g114080)

MGNPLQLKSLNHISLVCRSLDKSVDFYVNVLGFFPIKRPTSLAFNGAWLFNYGIGIHLLQSDDPESMTKNVHINPKDNHISFQCESMAAVENKLQQMKIEYVKNLVEENGIYVDQLFFHDPDGTMIEICNCDNIPIVPLSENSTIWSCSRFNCNIQNQQQQIQQMISM

>MtGLYI-13 (Accession No. Medtr4g125860)

MASLLKVSSFISPLHHKLNYVSFSPKFNHVSVRNERWNAPSITVKAQTAVEGDVINNESLSSNEQSDYGVVSVHHVGILCENLERSLDFYQNVLGLKINEARPHDKLPYRGTWLWVGSEMIHLMELPNPDPLTGRPQHGGRDRHTCIAIRDVSKLKAILDKAGVPYTLSRSGRPAIFTRDPDANALEFTQIDD

>MtGLYI-14 (Accession No. Medtr4g132260)

MAEIDLEWPKKDNRRLLHVVYRVGDLERTIKFYTEALGMKLLRQRDVPEEKYANAFVGFGDEHSHFAVELTYNYGVTSYDVGDGFGHFAIATQDVYKLVEHIRAKGGNITREAGPVQGGTTVIAFVKDPDGYTFALVQRPIVHDPFCQISLRVGDLERAIKFYEKALGLKVVRKVDNPENKYTIAILGYKEEDDATVLELTYNYGVTEYSKGTAYAQIAIGTDDVYKSADVVNLVTQELGGEITLQPGPIPGLNTKVTSFLDPDGWKTALVDNEDFLKELE

>MtGLYI-15 (Accession No. Medtr4g132270)

MSGFCVGLSLPTDIPHSLSHLVVMAEIDLEWPKKDNRRLLHVVYRVGDLERTIKFYTEALGMKLLRQRDVPEEKYANAFLGFGDEQSHFVVELTYNYGVTSYDVGDGFGHFAIATQDVYKLVEHIRAKGGNITREAGPVQGGTTVIAFVKDPDGYTFALVQRPIVHDPFCQISLRVGDLERAIKFYEKALGLKVVRKVDNPENKYTIAILGYKEEDDATVLELTYNYGVTEYSKGTAYAQIAVGTDDVYKSADVVNLVTQELGGKITRQPGPIPGLNTKVVSFLDPDGWKTVLVDNEDFLKELE

>MtGLYI-16 (Accession No. Medtr5g006360)

MAGVCLNHISRESNDINRLAKFYQEIFGFEEVESPKFGEFKVVWLRVPSSSLYLHLIERNPSNNLPEGPWSATSPVKDPSHLPRGHHLCFSVSNFQSFLQTLKDKGIETFEKSLPNGKIKQVFFFDPDGNGLEVASKEDS

>MtGLYI-17 (Accession No. Medtr5g006370)

MAAAEGVSLNHIARESTDVKRLSKFYQEMFGFEEVETPDFGELKIIWLRLPSSSLLIHLIQHSNGELAPSSSIPVKDPSHIRLGHHLCFSISNLHSFHNTLKDKGIETFETTNGNIKRVFFYDPDGNELEVFASIEDSS

>MtGLYI-18 (Accession No. Medtr5g009740)

MAASFRWLLQLHKDVPKAARFYSEGLDFTVNVCTLRWAELQSGPLKLALMHSPIDQSTQKGYSSLLSFTVTDINSTVTKLMALGAELDGPIKYEVHGKVAAMRCIDGHLLGLYEPV

>MtGLYI-19 (Accession No. Medtr5g021610)

MQLLDSLREQELKMEIEEVCEAQALPLLSLNHVSLLCRSVLESMQFYEDVLGFVPIKRPSSFKFTGAWFYNYGIGIHLIQNPDIDEFDTYMNESRPINPKDNHISFQCTDVELVKKRLEEKGMRYVTALVEDEGIKVDQVFFHDPDGYMIELCNCENIPIIPISSCTASFKPRSHSFKRSTSNFKCGGFMQNVMMQSLSMDMMNFAF

>MtGLYI-20 (Accession No. Medtr5g090990)

MANETHNQTGFKLVGCKNFIRTNPKTDRFKVKHFHHVEFWCTDATNTAHRFSHGLGMPIVAKSDLSTGNLTHASYLLRSGDLNFLFTAAYSPSISLSSPSSTASIPTFSPSTCFSFSNSHGLNVRALAVEVEDAELAYTVSVSYGALPSSPPVVLENGVKLAEVRLFGDVVLRYVSYNNPNQNQNLLFLPGFETLSGESSNSSLDFGIRQLDHANGNVPELSSALKYIKQFTGFHDFAEFTAEDVESGLNAVALANNDETVLLPLCEPVYGTKRKSTIETYLEHNEGAGFQHLALASEDIFKTLREMRKRSGVGGFEFMPSPPVTYYRNLKNRVGDVLSDEQIKECEELGILVDRDDQGTLLQIFTKPIGDRPTIFLEIIQRVGCMLKDEEGKEYQKGGCGGFGKGNFSELFKSIEEYEKTLETRRTA

>MtGLYI-21 (Accession No. Medtr5g091060)

MAIETETQTQTQTGFKLVGFKNFVRANPKSDRFNVKRFHHVEFWCTDATNTARRFSHGLGMPIVAKSDLSTGNLTHASYLLRSGDLNFLFSAAYSPSISLSSPSSTAAIPTFSASTCFSFSASHGLAVRAVAVEVEDAEVAFTTSVNLGAIPSSPPVILENNVKLAEVHLYGDVVLRYVSYNDLNPNQNPNLFFLPGFERVSDESSNSSLDFGIRRLDHAVGNVPELSSAVKYVKQFTGFHEFAEFTAEDVGTSESGLNSVVLANNEETVLLPMNEPVYGTKRKSQIETYLEHNEGAGLQHLALMSADIFRTLREMRKRSGVGGFEFMPSPPVTYYRNLKNRVGDVLSDEQIKECEELGILVDRDDQGTLLQIFTKPIGDRPTIFIEIIQRVGCMLKDEEGKEYQKGGCGGFGKGNFSELFKSIEEYEKTLETRRTA

>MtGLYI-23 (Accession No. Medtr8g076160)

MGIQEIGSYEAPLPLLSLNHVSILCRSVLDSMRFYEEILGFGLIKRPSSFKFNGAWLYNYGFGIHLLENPNYDEFDTPMSESRPINPKDNHISFQCTDVGLVKMRLEDMGMKYVTALVEDEGIKVEQVFFHDPDGYMIELCNCENIPIVPISSASGSFKARGQSFKKTVSNKCGFMENVMMRSLTKDMMNFAF

>MtGLYI-25 (Accession No. Medtr0003s0630)

MQKQEVKEEERNSNKKEEKNEKEGDEGTKESNQTPLMALNHISRLCRDVKESIDFYTKVLGFVLIERPQVLDFEGAWLFNYGVGIHLVQSKEEQKLPSPDAQHDLDPQDNHISFQCEDVKGMEKKLKEMKVKYKKRNLEAEDGTTMDQIFFNDPDGFMVEICNCENLKLTPADSQGKIKIPMDRHTPPVETNQNEHDNVK

>MtGLYI-26 (Accession No. Medtr0430s0010)

VAKTVNIVKEKWGKVMRELEPVKDGSTVTAFIEDPSGDRFELLGRRLTREPLCKVMLQADNLDCVIAFYEKAVGMKLLHKIVNPK

>MtGLYI-27 (Accession No. Medtr1239s0010)

MGTIRALGYVGFTATNIDRWKDFAPGVLGLQLSETWPDGTLVLRADAYQRRIFIHPGRVDEIRYIGWEVYDADSLEKLKSQLTGKRVPFVDLNEDETAHRAVIDGLKFLDTDGLHIEAFYGASQSKHEPFVSPVGQGPFVTGEQGLGHIVVHPENYSAAVAFYKDVLDFKISDYCTINTLGARDGHATFMHVNPRHHSLALANFPIGQRLNHIMLELETIDDVGCAYERALAAGAHILLDLGRHTNDDVFSFYVMTPSGWSVEIGCGGRRIDDTTWHVSHHTRPSSWGHNLDSTSRMVKVGNLDIHYHDSGSGDQVVVLLHGGGPGASSWSNFQRNIGPLSEHFRVLAIDMPHFGKSTKPEGRYLDRPWYAEVVGATLDTLNISKAHFVGNSLGGSVSMVLSVERPEIVDRLVLMGTMGSLPVFAPLPPEGAKHIIEYYHGEGPTREKLEAFLRSMIYDQNLITTDFIEERFIASTTPELLFVAQQPKLNTHFTQWQTADQVKHKSLLLYGRDDRVVPWDTSLLLLRLMPNADLHIFSRCGHWAQWERADEFNSVVANFL

>MtGLYI-28 (Accession No. Medtr1275s0010)

MLLGSRTDIRIAGESAEFGFTEIRHGLGGPAAIISRLRDQIPYTSLMWLAFGQHINAHEAHRIGLVNELVPDDQVLDRAMEVARAIAEVPPLAIRAEKQSLLRTQHQPFKEAVQYGTALFSMIQMSADAREGVQAFVEKRRPNFRMDRRREPAGRWRAGRFNAGSFMSRVVVVTGAASGNGLAIASRFLDHGDRVVAVDVSTDGLGARFQKEWRPYEERVIALTKDVSCQTDIDASRASQDLSFRVRPSTRGEGARGIPCMPEQCEGVVVNIASIATLVANPGRTSYATSKGALLQRTRSIAADYAHAGIRCNALCPGLIETAMTQWRRGGSEQRNQVLAKIPQNEVGTVDDVASAVMFISDPQSRYFNGAALVIKADHAAYVVSSLKEALTFWVDGMGATLEGRFKAGGPMLANVTGAVGADVSIALIEIAGQRLELLEYQGVTPNPGATLRPYDAGAMHLALNVDDVHAALRHVAQYGYRAQGVPQKAPTGSTAMYVVGPDGATIEFRQPEVA

>MtGLYI-29 (Accession No. Medtr1759s0010)

MKKIATVLFSALMLASTCASAQALCKAGKIDKIETDTTGNLLVTINDGVYSFSAKEVYSIIYQAYSENRNLFIYGNNCANGSPATRFAIPPPRAGVPILRDMAPACPRPVHPSAIPVPVPMKPAGLPLRISRRPAFGASTASSRRSRRAGCPASLRRRPSGPGHEQTPGCRISPCADRTHPSRRRPMIDHVYISVTDIEKSLAFYAEALKPLGWRIFGNYDSASGPESVPDLYGIGDDVYGKGAGVGSSIWLRKRHPGETGLYVGIVCDTNELVDAAYAAAIKAGGIDEGKPADRTYFAPGYYAANVADFDGNRLEFVHKAWNPKRHA

>AtGLYII-1 (Accession No. AT1G06130)

MPVISKASSTTTNSSIPSCSRIGGQLCVWPGLRQLCLRKSLLYGVMWLLSMPLKTLRGARKTLKITHFCSISNMPSSLKIELVPCSKDNYAYLLHDEDTGTVGVVDPSEAAPVIEALSRKNWNLTYILNTHHHDDHIGGNAELKERYGAKVIGSAVDKDRIPGIDILLKDSDKWMFAGHEVRILDTPGHTQGHISFYFPGSATIFTGDLIYSLSCGTLSEGTPEQMLSSLQKIVSLPDDTNIYCGRENTAGNLKFALSVEPKNETLQSYATRVAHLRSQGLPSIPTTVKVEKACNPFLRISSKDIRKSLSIPDSATEAEALRRIQRARDRF

>AtGLYII-3 (Accession No. AT2G31350)

MVMTHFSRLRQLLLLQPKFLSSQPRPLRSPPPTFLRSVMGSSSSFSSSSSKLLFRQLFENESSTFTYLLADVSHPDKPALLIDPVDKTVDRDLKLIDELGLKLIYAMNTHVHADHVTGTGLLKTKLPGVKSVISKASGSKADLFLEPGDKVSIGDIYLEVRATPGHTAGCVTYVTGEGADQPQPRMAFTGDAVLIRGCGRTDFQEGSSDQLYESVHSQIFTLPKDTLIYPAHDYKGFEVSTVGEEMQHNPRLTKDKETFKTIMSNLNLSYPKMIDVAVPANMVCGLQDVPSQAN

>OsGLYII-1 (Accession No. LOC_Os01g47690)

MVALLRSCRRLIPHLSACAAAAPSSSSSCAPRARPISRGLRLLPVVLAMAGYSSGSAAEGRRLLFRQLFEKESSTYTYLLADVGDPEKPAVLIDPVDRTVDRDLNLIKELGLKLVYAMNTHVHADHVTGTGLIKTKLPGVKSVIAKVSKAKADHFIEHGDKIYFGNLFLEVRSTPGHTAGCVTYVTGEGDDQPSPRMAFTGDALLIRACGRTDFQGGSSDELYESVHSQIFTLPKDTLLYPGHDYKGFTVSTVEEEVAYNARLTKDKETFKKIMDNLNLAYPKMIDVAVPANLLCGIQDPPPSKV

>GmGLYII-1 (Accession No. Glyma.02g220100)

RKDENPSHTNYSYLYVVVFVTNLQDNVKGCTDKVENGDKVSLGPDVTVLALLTPCHTQGHISYYVTGKEDEQPAVFTGDTLFIASCGKFFEETAEQMYQSLNVTLASLPKSTRVYRGHEYSVNNLQFAVTLEPDNLRIQKKLAWARNQWQAGQATIPSTIEDELETNPFMRVDLPEIQERVGCKSPVKALGEIRKQKDNWRG

>GmGLYII-2 (Accession No. Glyma.04g224100)

MKIYHVPCLRDNYSYLIVDKSTKEGAVVDPVEPQKVLEAANSHWVNLKLVLTTHHHGDHAGGNEKIKQLVPGIKVYGSLIDNVIGCTDKVENGDKESLGADIYILCLHTPCHTKGHISYYVTGKEEEQPAVFTGDTLFIADCGKFFKGTAEQMYQSLCVTLGSLPKPTRVYCGHGEKVGCKSPVEALRELRKLKDNWKG

>GmGLYII-3 (Accession No. Glyma.06g140800)

IVDESTKEGAVVDPVEPQKVLEAANSHGVNNLKLVLTTHHHGDHAGGNEKIKQLVLGMKVYGGSMDNIKGCTDKVENGDKMSLGADINILCLHTPCHTKGHISYCVTGKEEEVLRKEYNKLILKAMPKFIAGCGKFFEGTAEQIYQSLCVTLGSLPKPTRVYCGHEYAVRNLLFALTIEPDNLRIQQKLTWAKNQQQAGQSTIPSTIEEEMETNPFMRVNLPEIQGASLPVEALRELRKLKDKWKGVMELTNYCILHV

>GmGLYII-4 (Accession No. Glyma.11g126200)

MGDTKERSGFCVWPDARQLCLGKGLLYGFMRLFSIPLKTLRGASRSLRVNQFCSVVNLSSSLQIELVPCLRDNYAYLLHDVDTGTVGVVDPSEAAPIIDALSKKDLNLTYIMNTNHHPDHTGGNAELKERYGAKVIGSEIDKERIPGIDIYLSDGDNWMFAGHEVHILATPGHTEGHVSFYFPGSGAIFTGDTLFSLSCGKLLEGTPKQMLSSLKRIMSLPDDTSIYCGHEYTSSNSKFALSIEPENKELQSYAAHVANLRNKGLPTIPTTVKVEKACNPFLRTWSMEIRQKLNIATTADDAEALGVIQQAKDNF

>GmGLYII-5 (Accession No. Glyma.12g050800)

MNVLVFERSGFCVWPDARQLCLRKGLLYGFMRLFSIPLKTLRGASRSLRVDQFCSVVNLSSSLQIELVPCLRDNYAYLLHDVDTGTVGVVDPSEAAPIIDALSKKDLNLTYILNTNHHPDHTGGNAELKERYGAKVIGSEIDKERIPGIDIYLSDGDNWMFAGHEVHILATPGHTEGHVSFYFPGSGAIFTGDTLFSLSCGKLLEGTPEQMLSSLKRIMSLPDDTSIYCGHEYTLNNSKFALSIEPENKELQSYATHVSNLRNKGLPTIPTTLKVEKACNPFLRTWSIEIRQKLNIAATADDAEALGVIRQAKDNF

>GmGLYII-6 (Accession No. Glyma.13g261400)

MPIATKLYASNVTSTLNSKTNSNNGDLIYVILIVIPVIKLFLYSTIHNQNPTTSTLQSLSSIQPFWWQLLFMLQKMLRLHFTTALSHFASKASPFPLTPVSVTVSRAIVCNNPTRFRSQMGSFSTSSSSSSKLLFRQLFEKESSTYTYLLADASHPEKPALLIDPVDRTVDRDLSIIEQLGLKLVYAMNTHVHADHVTGTGLIKSKVPSVKSVISKASGATADLYVEPGDKVQIGDLFLEVRATPGHTKGCVTYVTGDAPDQPQPRMAFTGDTLLIRGCGRTDFQGGSSEQLYKSIHSQILTLPKSTLIYPAHDYKGFTVSTVGEELQNNPRITKDEETFKNIMGNLNLSYPKMIDIAVPANMVCGIQSNPKQAEAS

>GmGLYII-10 (Accession No. Glyma.15g245500)

LRSQMCSFSTTSFSSSSSKLLFHQLFEKKSSTYTYLLADASHPEKPTLLIDPVDRTVDRDLSLIEQLGLKIVYTMNTHVHADHVTGTGLIKGKVPSVKSVISKASGATVDLYVEPGDKVHIGDLFLEVRATPGHTKGCVTYVTGDAPDQPQPRMAFTGDTLLIRGCGRTGFQIYTCSKLLEQRRKWVKV

>GmGLYII-11 (Accession No. Glyma.18g163500)

TGKKRIPAIDIHLNDGDKWMCAGHEVRVMDTPGHTQGHISFYFPGSGVIFTGDTFFNLSCGKLFEGTPQQVVLNCTCPFFLFFFF

>GmGLYII-12 (Accession No. Glyma.20g118000)

MATHRLALIIQNPSNDDEFLLVKQSRPPKFHDEEYDSFVDSDLWDLPSAQLNPLLAESEPPVELELAVSHSESQDVDLRKFDIRSALNEVFGQLGFGAVDGGGWKFHKYVKEAAFGPDLPVNTVFIVGKLVAAEDKDFRDSYRWKSVRSCLNWILEVKPHGDRVGPLVVIGLINESSISTKWKVPPAINYQEYPPGNIIIPMGSRTLRPFHTTNLVVFAPENVSNDSGENNFIVRGDALIVDPGCLSEFYGELEKIVTALPRRLVVFVTHHHPDHVDGLSVIQKCNPDATLLAHEKTMHRISRDVWSLGYTPVTGDEDIDIGGQRLRVIFAPGHTDGHMALLHANTHSLIVGDHCVGQGSATLDIKAGGNMSEYFQTTYKFLELSPHALIPMHGRVNVWPKQMLCGYLKNRRSREANIVKAIEGGAKSLFDIIVYVYSDVDRRAWIAASSNVRLHVDHLAQQHKLPKDFSIQKFKNTCGLHFLSRWIWAYGSGSLSHQIGKSPFLVAGVLAGIAGIAVLYCQRKFTK

>MtGLYII-1 (Accession No. Medtr1g032500)

MTIEVLVLGAGQEVGKSCVIVKINGKRIMFDCGMHMRHTDHSRYPDFKKISDSGNFNDALDCIIITHFHLDHVGALAYFTEVCGYSGPVYMTYPTKALSPLMLEDYRKVMVDRRGEEEQFTSDHIAECMKKVIAVDLKQTVQVDEDLQIRAYYAGHVIGAAMFYVKVGDAEMVYTGDYNMTPDRHLGAAQIDRLRLDLLITESTYATTIRDSKYAREREFLKAVHKCVSGGGKVLIPTFALGRAQELRILLDDYWERMNLKVPIYFSSGLTIQANTYHKMLIGWTSQKIKDTYSTHNAFDFKNVHKFERSMLDAPGPCVLFATPGMLIGGFSLEVFKHWAPSEKNLVALPGYCMAGTVGHRLTSGKPTKVDTDPDTQIDVRCQIHQLAFSAHTDSKGIMDLVKFLSPKHVMLVHGDKPKMVSLKERIDSELGIPCSHPANNEIVTISSTQYVNAEASDTFTKNCLNPNFKFQKCSSMDTCNSTLIDRNLTPELQVEDERVADGVLVMENNNNKKAKIVHEDEILLMLDEKKHEV

>MtGLYII-2 (Accession No. Medtr1g050492)

MGTSVQVTPLCGVYNENPLSYLVSIDSFNILIDCGWNDHFDPSLLQPLSRVASTIDAVLLSHPDTLHLAALPYAIKHLGLSAPVYSTEPVYRLGLLTMYDHFLSRKQVSDFDLFTLDDIDSAFQTVTRLTYSQNHHLSGKGEGIVIAPHTAGHLLGGTIWKITKDGEDVIYAVDFNHRKERHLNGTVLGSFVRPAVLITDAYNALNNQPYRRQKDKEFGDILKKTLRAGGNVLLPVDTAGRILELILMLESYWADENLNYPIYFLTYVASSTIDYVKSFLEWMSDSIAKSFEQTRENIFLLKNITLLVSKADLDNAPDGPKVVLASMASLEAGFSHDIFVEWGNDVKNLVLFTERGQFGTLARMLQADPPPKAVKVTVSKRVPLVGEELIAYEEEQNRIKKEEALKASLMKEEEFKASQGADNNAIDPMIIDTGNSQPSPEVAVPKNGGYRDVFIDGFVPPSSSVAPMFPCYENITEWDDFGEVINPDDYVIKEEDMDQAANNVGGDLNGKLDESAASLIFDTKPSKVISDERTVQVRCSLVYMDFEGRSDGRSIKNILSHVAPLKLVLVHGSAEATDHLKQHCLKNVCPHVYAPQIEETIDVTSDLCAYKVQLSEKLMSSVLFKKLGEYEVAWVDAEAGKTENDMLSLLPVSGAPHPHKSVLVGDLKLADFKQFLSTKGVPVEFAGGALRCGEYVTVRKVGDATQKGAGSGTQQIIIEGPLCEDYYKIRDYLYSQFYLL

>MtGLYII-3 (Accession No. Medtr1g110300)

MATHKLALIIQNPSNQNEFLLIKQSRPPKFNDEEYDSFLDSDLWDLPSVQLNPLQPQSDPPVEVQISVSHSDEFNFSEFDIHSALKEVFGELGFGIVERGEWKFHKYVKEPAFGPGLPVNTVFIAGKLVDDEIKDFSDSYKWMSIQSCLNWLLEVIPHGDRVGPLVVVGLVNDSSVSANWEAPPAINYQEYPTGVILIPMGSRTAKPFHTTNLVVFAPENVPNASKDNQLIVYGDALIVDPGCLSKFHGELKNIVTALPRRLVVFVTHHHRDHVDGLSVIQKCNPDAILLAHENTMRRISRDDWSLGYTSVTGDEDIYIGGQKLKVIFAPGHTDGHMALLHVNTHSLIVGDHCVGQGSALLDINSGGNMSEYFETTYKFLELSPHALIPMHGRVNVWPKQMLCEYLKNRRSREAAILKAIEGGAKTLFEIVAYVYSNVDRRAWIPASSNVRLHVDHLAEQHKLPKEFSIRNFKNTCGLHFLSRWIWGYTSCSIHPRKSSFLIAGVLVGIAVLVHCSAKTKFRK

>MtGLYII-4 (Accession No. Medtr2g006180)

MSSVKKRESNGGTINRETEDQLIVTPLGAGNEVGRSCVYMTYKGKTVLFDCGIHPGYSGMAALPYFDEIDPSTVDVLLITHFHLDHAASLPYFLEKTTFKGRVFMTYATKAIYKLLLSDYVKVSKVSVDDMLYDEQDINRSMDKIEVIDFHQTVEVNGIRFWCYTAGHVLGAAMFMVDIAGVRVLYTGDYSREEDRHLRAAETPQFSPDVCIIESTYGVQHHQPRHTREKRFTDVIHSTISQGGRVLIPAYALGRAQELLLILDEYWANHPELQNIPIYYASPLAKKCLTVYETYTLSMNDRIQNAKSNPFAFKHISALSSIDIFKDVGPSVVMASPGGLQSGLSRQLFDMWCSDKKNSCVIPGYVVEGTLAKTILNEPKEVTLMNGLSAPLHMQVHYISFSAHADSAQTSAFLEELNPPNIILVHGAANEMGRLKQKLMTQFADRNTKILTPKNCQSVEMYFNSQKMAKTIGKLAEKTPEVGETVSGLLVKKGFTYQIMAPDDLHVFSQLSTANVTQRITIPYSGAFCVIQSRLKQIYESVEPSVDEESGVPMLLVHDRVTVKHESEKHVSLHWASDPINDMVSDSVVALVLNINRDLPKIVAESDATKIEEENEKKTEKVMQALLNSLFGNVKVGENGKLIINIDGNVAELNKESGEVESENEGLKERVRTAFRRIQSSVKPIPLSAP*

>MtGLYII-5 (Accession No. Medtr2g018660)

MATSNGTDDGTPPSESALIFLGTGCSSMVPNVLCLINPSDPPCSVCAQSLSIPPEKNPNYRCNTSMLIDYCGSGSNHNYILIDVGKTFRETVLRWFVHHRIPKIDSIILTHEHADAVLGLDDVRAVQPFSPTNDIDPTPIYLSQHSMDSIEEKFPYLVQKQRKEGQEIRRVAQMAWNIITDDCNQPFFASGLKFTPLPVMHGEDYICLGFLFGEKSRVAYISDVSRIPASTEYVISKSGAGQLDLLILDSLYRTGSHNVHLCFPQTLEIVKRLCPKQTLLIGMTHEFDHHKDNEFLKEWSRREGIPVQLSHDGLRVPINL

>MtGLYII-6 (Accession No. Medtr2g072190)

MLKSQFIKFTPFFPYKPSFSSLSISTTIKLKSQMASYSTSSSSSKLLFRQLFEKESSTYTYLLADASHAEKPAVLIDPVDRTVDRDLSLIQELGLKLVYAMNTHVHADHVTGTGLIKSKVPDVKSVISKASGATADLYVEQGDKIRFGDLFLEVRATPGHTLGCLTYVTGDGPDQPQPRMAFTGDTLLIRGCGRTDFQGGSAEKLYKSIHSQIFTLPKDTLLYPAHDYKGFSVSTVGEEMQYNPRLTKDEETFKNIMANLNLSYPKMIDVAVPANMVCGVQSKTS

>MtGLYII-8 (Accession No. Medtr2g101390)

MAQILNFRNFLFLPSYKPTTHFRLRFLSTLVSSSSRRSNINAPPLHLRRRSTTTSTTPMEVEENSSVGFNKRRAEGTENSGLPKKNLQLKVRKLNPINTISYVQVLGTGMDTQDTSPAVMLFFDKQRFIFNAGEGLQRFCTEHGIKLSKIDHIFLSRVCSETAGGLPGLLLTLAGMGDEGMTVNVWGPSDLKYLVDAMRSFIPNAAMVHTKSFGPTFGTESTVKSQSDPIVLVDDEVVKISAIILQPCQIPSQKTDHSIDIADSLNGKKLLAAKPGDMSVVYVCELPEIQGKFDPEKAKALGLRPGPKYRELQLGNSVESDRQKNVMVHPSDVMDPSIPGPVVLVVDCPTESHLEALLSAKSLDTYGDQVGNLPKAGKSVSCVIHLTPESVVCCSNYQNWMKTFSSAQHIMAGHEKKNIEVPILKASARIATRLNYLCPRFFPAPGFWSLPNQNCSKPVSLASSEDSFSAPSNVIYAENLLKFTLRPYVNLGLDRSCIPPKASSSEIIDELLLEIPEVVEAAQHVRQLWEDSSQAKEDSIPLADHSEVIEEPWLSEDGITPACLENIRRDDLEIVLLGTGSSQPSKYRNVTSIYINLFSKGGLLLDCGEGTLGQLKRRYGVSGADDVVRSLSCIWISHIHADHHTGLTRILALRRDLLKGVPHEPVLVVGPRMLKRYLDAYHRLEDLDMLFLDCKHTFEASLADFENDLQETVNSLDLNNNNAEINASKVDSTLFARGSPMQSLWKRPGSPVDKDTVYPLLRKLKGVIQEAGLNTLISFPVVHCSQSYGVVLEAEKRINSVGKVIPGWKIVYSGDTRPCPELIKASRDATVLIHEATFEEGMVLEAIARNHSTTNEAIETGEAANVYRIILTHFSQRYPKIPVINKEHMDITCIAFDLMSINIADLPVLPKVLPYLKLLFRNDMTVDESNDVVVTVDESDDVVDVATSAS

>MtGLYII-9 (Accession No. Medtr3g089020)

MATLTSLPPLPHSLLSLRSKPTRLSVSASALSASGNDGSTSRVPQKRRRRIEGPRKSMEDSVQRRMEQFYEGNDGPPLRVLPIGGLGEIGMNCMLVGNHDRYILIDAGIMFPDYDDLGVQKIIPDTTFIRKWSHKIEALVITHGHEDHIGALPWVIPALDSNTPIFASSFTMELIKKRLKEHGIFLPSRLKIFRTKNKFVAGPFEIEPIRVTHSIPDCCGLVLRCSDGTILHTGDWKIDETPLDGKVFDREGLEELSKEGVTLMMSDSTNVLSPGRTTSESVVADSLLRHISASKGRVITTQFASNLHRIGSVKAAADLTGRKLVFVGMSLRTYLEAAWKDGKAPFDPSTLVKAEDIDAYAPKDLLIVTTGSQAEPRAALNLASFGSSHAFKLTKEDIVLYSAKVIPGNESRVMEMMNRISEIGSTIVMGRNENLHTSGHAYRGELEEVLRIVKPQHFLPVHGEYLFLKEHESLGKSTGIRHTAVIKNGEMLGVSHLRNRRVLSNGFISLGKENLQLKYSDGDKAFGTSGELFLDERMRIALDGIIVVSMEIFRPKNLESLAGNTLKGKIRITTRCLWLDKGKLLDALYKAAHAALSSCPVKSPLPHMERTVSEVLRKMVRKYSGKRPEVIAIAIENPGAVFADEINTKLSGKSQVGPGISTFRRSVDEHRKENQSTALQIRDDGIDIEGLLVEIETITTAAEGDLSDSGESDEFWKPFIASSVEKSIKANNGYVSRKEHKSNTKQDDSEDIDEAKSEEMSDSEPESSKSEKKNKWKTEEVKKLIDLRSDLRDRFKVVKGRMALWEEISQSLLADGISRSPGQCKSLWTSLALKYEEIKNGKDSRKNWQYLEDMERILSSDETPATN

>MtGLYII-10 (Accession No. Medtr4g068100)

MTDIKERSVLCALPDVRQICFRKGLLYGFSRVFSIPLKTLRGASRSLRVDQFCSVVNISSSLMIELVPCLRDNYAYILYDVDTGTVGVVDPSEAAPVIDALTKKNLNLTYILNTHHHHDHTNGNTELKERYGAKVIGSDVDKERIPGIDIYLSDGDKWMFAGHEVQIMATPGVTQGHISFYFPGSAAIFTGDTLFSLSCGKIYEGTPEQMLSSLKKITSLSDDTSIYCGHEYTLDNSKFALSIDPQNKELQSYASHVAQLRNKGLPTVPTTLKMEKACNPFLRTWSMEIRRKLKVAASADDAEALGVIRQAEDNF

>MtGLYII-11 (Accession No. Medtr4g103770)

MVTCNSISLSHNLHFYTRFHRLHPTRRSHYCRFRSNALPRDTDGAKVVHKRPRRIEGPRKSMEDSVQRKMEQFYEGSDGPPLRVLPIGGLGEIGMNCMLVGNHDRYILVDAGVMFPGDDELGVQKIIPDTTFIKKWSHKIEAVVITHGHEDHIGALPWVIPMLDSQTPVFASSFTMELIRKRLKDHGIFVPSRLKVFRTRKKFVAGPFEIEPITVSHSIPDCCGLVLRCSDGTILHTGDWKIDETPLDGKVFDREALEELSKEGVTLMMSDSTNVLSPGRTMSESVVADALLRHISAAKGRVITTQFSSNIHRLGSLKAAADLTGRKLVFAGMSLRTYLDAAWKDGKVPIDSSTLVKVEDMHAYAPKDLLIVTTGSQAEPRAALNLASYGSSHAFELTKEDTVLYSAKVIPGNESRVMEMLNRISEIGPTIVMGKNECLHTSGHAYRGELEEVLRIVKPQHFLPIHGELLFLKEHELLGKSTGIRHTAVIKNGEMLGVSHLRNRKVLSNGFISLGKENLELKYSDGDKAFGTSSELFIDERLRIALDGIIVVSMEVCRAQSLDSSVENTLKGKIRITTRCLWLDKGKLLDALHKAAHASLSSCPVNCPLAHMEKTVSEMLRKMVRKYSGKRPEVIAVAIENPGAVLATEINTKLSGKSYVGGISTFRNVVHKENQSTKMQMRGMIGMLEFWRRSRMRRTERRHGYIWRTWKAFDNEALAKK

>MtGLYII-13 (Accession No. Medtr8g017270)

MVVTSSIRLLPSSLTSLVHHRSPYSRRLLRPSSISFPLSPIHSLSSNGIGEVDSHVDQSQVIFIGTGTSEGIPRVSCLTNPSTKCPVCTKAAKPGDKNRRLNTSILVRHSNGTGTHNILIDAGKFFYHSALQWFPKFGIRTLDAVIITHSHADAIGGLDDLRDWTNNVQPSIPIYVAKRDFEVMKKTHYYLVDTSVIIPGAAVSALQFNSISEEPFFVHGLKFTPLPVWHGQGYRSLGFRFGNICYISDVSEIPEETYPLLKDCELLIMDALRPDRSSATHFGLPRALEEVRKIQPKRTLFTGMMHLMDHEEVNDYLTKLLESEGLDAQLSYDGLCIAVRL

>MtGLYII-14 (Accession No. Medtr2166s0010)

MKNLNWQLGGLRIVALSDGTHPFPVDTVFRDISKDDIRRDLDRAFLEPPVQGSINAFLVDTGTKRILVDSGAGVLYGDCCGKLLANLRAAGYAPEQIDEVLLTHLHKDHVGGIVTNGRMTFPNAVVRVNEIEANYWLDPDNKAQAPAFLASFFDAAAASVAPYIAAGRFRTFRGEATLAPGIRAVPMPGHTPGHTAYLIESGDAGLLAWGDIVHVAAIQLQDPDATVQYDSDADAARRTRRDTLKRVANKRYLVGAAHIAFPGLGHLRRDGEQYDWVPVNYDATPLR

>AtDJ1-c_C

ANGSEAVELVSIADVLRRAKVDVTVSSVERSLRITAFQGTKIITDKLIGEAAESSYDLIILPGGHTGSERLQKSKILKKLLREQHESGRIYGATNSSSTVLHKHGLLKEKRTTVYPSESDEPMNQQMIEGAEVVIDGNVITSLGLATVTKFSLAIVSKLFGHARARSVSEGLVHEYPRQ

>AtDJ1-e_C

GDYVEDYGINVPFRALQALGCKVDAVTPNKKKGEVCATAVYDLEDGRQIPAEKRGHNFFVTASWDDICVDDYDCVVVPGGRSPELLVMNEKAVALVKSFAEKDKVFAAIGQGKLLLAATGVLKGKRCASGKGMKVMVKVAGGEAVMEKGCVTDGKVVTAASATDLPAFLFDLSTALGLTVMF

>AtDJ1-f_C

GDCVEDYSINVPFKAFQALGCKVDAVTPTKKRGEKCATIVHDLEDGRQLPTEKFGHNFYVTVAWDDVSVDDYDCIVVPGGRSPELLVMNPKAVELVRKFVEKGKFVAAIGMGNWLLAATGALKKKRCASSYGTKVAVKVAGGEIVESERCVTDDKLVTAASTSDLPAFLYALSTALGLSVVF

>OsDJ-1A_C

ANGTEEMELITIIDVLRRADADVVVASAENAGVEIVARHGMRIVADTTLDEAAADDQTSSFDLIILPGGTPGAKTMSSNEKLVTLLKKQAAASKPYGAIGAATAHVLEPHGLLEGKKAADQDGGDECESRVVVDGNVITSGGTGTAMEFAVAAVEKLLGRDVAQRVAEGLLFA

>OsDJ-1F_C

ADYVDDYEANVPFRALAGVGCRVEAACPTKRKGEACVTAIYDATPAAASDERRGHNFAVTADWGDVDADRYACVVVPGGRAPELLATRGEAVALVREFAGKGKVVASIDQGHLLLAAVGLLDGRSCASGVATRVVAGLAGAASVRHGGAVADGKLVTAASWPDLAEFIAHIISLLGITVSF

>MtDJ-1E_C

AHGSEEIEVVTLIDILRRAKANVVVASVEKTLGVMASQGTKIVADKLISDIQESAHDLIILPGGTAGAERLSKSRILKKLLKEQNSAGRIYGAVCSSPAILHKQGLLKDKKATAHPSALNKLKDGAVNDAVVVIDGKVITSEGLATVTDFALAIVSKLFGNGRARSVAEGLVFEYPRK

>AtDJ1-c_N

MGSLGYSISMIASLSPTLMESRLISSMGCVSMTVAPSFSSVSVVSSSLGTTRRDRTLKLRSSMSPGMVTTLDSDVGVGSSATTKKVLVPIGYGTEEIEAVVLVDVLRRAGADVTVASVEQKLEVEGSSGTRLLADVLISKCADQVYDLVALPGGMPGAVRLRDCEILEKIMKRQAEDKRLYGAISMAPAITLLPWGLLTRKRTTGHPAFFGKLPTFWAVKTNIQISGELTTSRGPGTSFQFALSLAEQLFGETTAKSIEEFLLLRDG

>AtDJ1-e_N

MASAVQKSALLLCGDYMEAYETIVPLYVLQSFGVSVHCVSPNRNAGDRCVMSAHDFLGLELYTELVVDQLTLNANFDDVTPENYDVIIIPGGRFTELLSADEKCVDLVARFAESKKLIFTSCHSQVMLMAAGILAGGVKCTAFESIKPLIELSGGEWWQQPGIQSMFEITDCVKDGNFMSTVGWPTLGHGIKLLLESLGGKVCSLEKKQASVLFLIGD

>AtDJ1-f_N

MGSMAQKSVLMLCGEFMEAYETIVPLYVLQAFGVSVHCVSPGRKTGDKCVMAAHDLLGLEIYTELVVDHLTLNANFDGVIPDQYDAIIIPGGRFTELLSADEKCVSLVARFAELKKLIFTSCHSQLFLAAAGLLTGGMKCTAFESMKPFIELSGGAWWQQPGVQTLFEITDCVKDGSFMSTMGWPTLGHSLKVLLESLGSKISSSKENHQTSLLFLIGD

>OsDJ-1A_N

MAAQASPPTKKVLVPIVAGTEPVEAAVPIDVLRRAGADVTVASADDGELVVEVMYGVRIVADALVAGGDCAAAHFDLIVLPGGVPGAANLGGCAALEAMVRRHAATGGLYAAICAAPPLALASWGMLNGLKATAHPLFVDKFPPEVAAVDASVVVDASAVTSRGPATSTEFALALVEQLYSKNKAEQIAKEMLVRYDA

>OsDJ-1F_N

MAPCKKVLMLCGDYMEDYEAAVPFYALAAFGVAVDCVAPGKKPPGDACLTAVHEFLGHDLYTELPGHRFAVTADFAAAAAADASRYDALVVPGGRFVERLSVDPLAVSLVAAFAGEGETATRRRPVVVTCHSQLLLAAAGAMRGVRCTAFFSMRRVVELAGGTWVEPDPLGLCVADGNVLSAIGWPAHGEIIRELLRAMGARVAGGRGQAVLFLCAD

>MtDJ-1E_N

MSFLLLLLPQPSTATRLSPFTISTSTISLKPLSTLSPPRSIPNSTLSISTSPPPTPTNAPPPKKVLLPIGFGTEEMEAVILIHVLRRAGAHVTVASVEPQLQVEAASGTKLVADASISECSDQIFDLIALPGGMPGSARLRDCDALRIITCKQAEENRLFGAINAAPAVTLLPWGLLKRKKITCHPAFFHKLPTFWAVKSNIQVSNGLTTSRGPGTAYMFALTLVEQLFGESIAREVAEFLLMRTD

>AtDJ-1C (Accession No: AT4G34020)

MGSLGYSISMIASLSPTLMESRLISSMGCVSMTVAPSFSSVSVVSSSLGTTRRDRTLKLRSSMSPGMVTTLDSDVGVGSSATTKKVLVPIGYGTEEIEAVVLVDVLRRAGADVTVASVEQKLEVEGSSGTRLLADVLISKCADQVYDLVALPGGMPGAVRLRDCEILEKIMKRQAEDKRLYGAISMAPAITLLPWGLLTRKRTTGHPAFFGKLPTFWAVKTNIQISGELTTSRGPGTSFQFALSLAEQLFGETTAKSIEEFLLLRDGYQNPKNKEFNSIDWSLDHTPRVLIPVANGSEAVELVSIADVLRRAKVDVTVSSVERSLRITAFQGTKIITDKLIGEAAESSYDLIILPGGHTGSERLQKSKILKKLLREQHESGRIYGATNSSSTVLHKHGLLKEKRTTVYPSESDEPMNQQMIEGAEVVIDGNVITSLGLATVTKFSLAIVSKLFGHARARSVSEGLVHEYPRQ

>AtDJ-1E (Accession No: AT2G38860)

MASAVQKSALLLCGDYMEAYETIVPLYVLQSFGVSVHCVSPNRNAGDRCVMSAHDFLGLELYTELVVDQLTLNANFDDVTPENYDVIIIPGGRFTELLSADEKCVDLVARFAESKKLIFTSCHSQVMLMAAGILAGGVKCTAFESIKPLIELSGGEWWQQPGIQSMFEITDCVKDGNFMSTVGWPTLGHGIKLLLESLGGKVCSLEKKQASVLFLIGDYVEDYGINVPFRALQALGCKVDAVTPNKKKGEVCATAVYDLEDGRQIPAEKRGHNFFVTASWDDICVDDYDCVVVPGGRSPELLVMNEKAVALVKSFAEKDKVFAAIGQGKLLLAATGVLKGKRCASGKGMKVMVKVAGGEAVMEKGCVTDGKVVTAASATDLPAFLFDLSTALGLTVMF

>AtDJ-1F (Accession No: AT3G54600)

MGSMAQKSVLMLCGEFMEAYETIVPLYVLQAFGVSVHCVSPGRKTGDKCVMAAHDLLGLEIYTELVVDHLTLNANFDGVIPDQYDAIIIPGGRFTELLSADEKCVSLVARFAELKKLIFTSCHSQLFLAAAGLLTGGMKCTAFESMKPFIELSGGAWWQQPGVQTLFEITDCVKDGSFMSTMGWPTLGHSLKVLLESLGSKISSSKENHQTSLLFLIGDCVEDYSINVPFKAFQALGCKVDAVTPTKKRGEKCATIVHDLEDGRQLPTEKFGHNFYVTVAWDDVSVDDYDCIVVPGGRSPELLVMNPKAVELVRKFVEKGKFVAAIGMGNWLLAATGALKKKRCASSYGTKVAVKVAGGEIVESERCVTDDKLVTAASTSDLPAFLYALSTALGLSVVF

>OsDJ-1A (Accession No: LOC_Os01g11860)

MAAQASPPTKKVLVPIVAGTEPVEAAVPIDVLRRAGADVTVASADDGELVVEVMYGVRIVADALVAGGDCAAAHFDLIVLPGGVPGAANLGGCAALEAMVRRHAATGGLYAAICAAPPLALASWGMLNGLKATAHPLFVDKFPPEVAAVDASVVVDASAVTSRGPATSTEFALALVEQLYSKNKAEQIAKEMLVRYDAGYTIDEVNSVQWKCNGTPKVLVPVANGTEEMELITIIDVLRRADADVVVASAENAGVEIVARHGMRIVADTTLDEAAADDQTSSFDLIILPGGTPGAKTMSSNEKLVTLLKKQAAASKPYGAIGAATAHVLEPHGLLEGKKAADQDGGDECESRVVVDGNVITSGGTGTAMEFAVAAVEKLLGRDVAQRVAEGLLFA

>OsDJ-1F (Accession No: LOC_Os11g37920)

MAPCKKVLMLCGDYMEDYEAAVPFYALAAFGVAVDCVAPGKKPPGDACLTAVHEFLGHDLYTELPGHRFAVTADFAAAAAADASRYDALVVPGGRFVERLSVDPLAVSLVAAFAGEGETATRRRPVVVTCHSQLLLAAAGAMRGVRCTAFFSMRRVVELAGGTWVEPDPLGLCVADGNVLSAIGWPAHGEIIRELLRAMGARVAGGRGQAVLFLCADYVDDYEANVPFRALAGVGCRVEAACPTKRKGEACVTAIYDATPAAASDERRGHNFAVTADWGDVDADRYACVVVPGGRAPELLATRGEAVALVREFAGKGKVVASIDQGHLLLAAVGLLDGRSCASGVATRVVAGLAGAASVRHGGAVADGKLVTAASWPDLAEFIAHIISLLGITVSF

>GmDJ-1A.1 (Accession No: Glyma.02G131600)

MHSINFKRQGGMPGSARLRDCDVLRKITCRQAEENSLYGAICAAPAVSLLPWGLLKKKKVSRGLTTSRGPGTSYQFALSLAEQLFGESVAKEVAELMLMRTDDDNAAKKEFNKVEWSVGHHTPSVLVPIVHGSEEIEVVTVVDILRRAKAKVIVASVEKSLEVLASQGTKIVADILIGDAQESPYDLIILPGGTAGAQRLSKSRILKKLLKEQNSAKRIYGAVYSSLAILQKQGLLKDKRTTAHPSVLVKLKDEEINGAKVDIDGKLITSEVLATVTDFALAIVSKLFGNGRARSVAEGLVFEYPKECM

>GmDJ-1A.2 (Accession No: Glyma.02G131600)

MGAFEEKEDNLPRFWAIKSNLQVSRGLTTSRGPGTSYQFALSLAEQLFGESVAKEVAELMLMRTDDDNAAKKEFNKVEWSVGHHTPSVLVPIVHGSEEIEVVTVVDILRRAKAKVIVASVEKSLEVLASQGTKIVADILIGDAQESPYDLIILPGGTAGAQRLSKSRILKKLLKEQNSAKRIYGAVYSSLAILQKQGLLKDKRTTAHPSVLVKLKDEEINGAKVDIDGKLITSEVLATVTDFALAIVSKLFGNGRARSVAEGLVFEYPKECM

>GmDJ-1A.3 (Accession No: Glyma.02G131600)

MQDNLPRFWAIKSNLQVSRGLTTSRGPGTSYQFALSLAEQLFGESVAKEVAELMLMRTDDDNAAKKEFNKVEWSVGHHTPSVLVPIVHGSEEIEVVTVVDILRRAKAKVIVASVEKSLEVLASQGTKIVADILIGDAQESPYDLIILPGGTAGAQRLSKSRILKKLLKEQNSAKRIYGAVYSSLAILQKQGLLKDKRTTAHPSVLVKLKDEEINGAKVDIDGKLITSEVLATVTDFALAIVSKLFGNGRARSVAEGLVFEYPKECM

>GmDJ-1C.1 (Accession No: Glyma.11G207900)

MEDYEAMVPFQALQAFGLAIYPRKKSDDVCCTAIHVLADTQTYSETVGHNFALNATFDEVDASSYDGLWVPGGRAPEYLAHIPGVVELVTKFVSLGKQIASICHGQLILAAAGVVEGRKCTLFLLLNQCWLLLAFGGKISGFDKKILFICGDYMEDYEVKDHFQSLQALGSHVDAVCPSKKAGDTCPTAKPGHTFALTATFDDVDPSGYDALVIPGGQAPEYLALNESVIALILSAAGVLKGRKCSAYPAVKLNVVLSGAAWLEPESISRCFTDGNLVTGAAWPGHPEFIAQLMALLGIQVSF

>GmDJ-1G.1 (Accession No: Glyma.18G046000)

MASKRILLLCGDFTEDYEAMVPFQALQAFGLTVDTVCPGRKAGDVCRTAIHGIHGDQTYSEMIGHKFVLNATFDEVDASSYDVLWVPGGRSPEYLSRVPGVLELVTKFVSLGKLIASTCHGPLILAASGVLKGRKCTGFPSLKPVLVDAGADWVDPDTMTTTVEDGGFITSTTYEGQPEIISLLVKALGGKISGTKKKILFICGDFVEDFQAKVPFQSLQSLGCHVDAICPSKFAGDFCPTAVHDFEGDQTYSEKHGHHFDLTVAFDDVDPSDYDALVIPGGRSPEYLSLMDPILDLVRHFFLNNKPVGSIGHGQQILAAAGVLKGRKCTAYPDVKLHVVLSGATWLEPDPISRCFTDGNLVTGAAWQGLPEFIAQLMALLGIRVSF

>MtDJ-1B (Accession No: Medtr3g064093)

MKFCWFQTYIETVGHKFTLNRTFDEIDHTNLVDMLVNSGQEIIACICHGHMIQAAANLLEGPKCTAFPPLKLVLIAAGAFWFEHYVNNCSGW

>MtDJ-1C (Accession No: Medtr3g064115)

MELEDMLVNSGQEIIACFCHGHLILAAANLLEGCKCTDFPPLKPVLIAAGAHWVEHLYLALFIALVKPFMENKKPVASICHSQHILAAAGVLKY

>MtDJ-1E (Accession No: Medtr4g085900)

MSFLLLLLPQPSTATRLSPFTISTSTISLKPLSTLSPPRSIPNSTLSISTSPPPTPTNAPPPKKVLLPIGFGTEEMEAVILIHVLRRAGAHVTVASVEPQLQVEAASGTKLVADASISECSDQIFDLIALPGGMPGSARLRDCDALRIITCKQAEENRLFGAINAAPAVTLLPWGLLKRKKITCHPAFFHKLPTFWAVKSNIQVSNGLTTSRGPGTAYMFALTLVEQLFGESIAREVAEFLLMRTDDDNVSKKEFNEIDWSVGHHPPSVLIPIAHGSEEIEVVTLIDILRRAKANVVVASVEKTLGVMASQGTKIVADKLISDIQESAHDLIILPGGTAGAERLSKSRILKKLLKEQNSAGRIYGAVCSSPAILHKQGLLKDKKATAHPSALNKLKDGAVNDAVVVIDGKVITSEGLATVTDFALAIVSKLFGNGRARSVAEGLVFEYPRK
